# Water *vs.* cucurbituril rim: a fierce competition for guest solvation†
†Electronic supplementary information (ESI) available: Preparation and characterization of guests **3a–3k** and their precursors; determination of binding affinities by competitive NMR titrations and ITC; computational procedures and data; Cartesian coordinates of the optimized structure of complexes **3a**·CB[7] and **3e**·CB[7]. See DOI: 10.1039/c5sc04475h


**DOI:** 10.1039/c5sc04475h

**Published:** 2016-02-17

**Authors:** Xiaoxi Ling, Stefan Saretz, Lifeng Xiao, John Francescon, Eric Masson

**Affiliations:** a Department of Chemistry and Biochemistry , Ohio University , Athens , Ohio 45701 , USA . Email: masson@ohio.edu

## Abstract

The impact of remote substituents on the affinity of cucurbit[*n*]urils (CB[*n*]) towards a homologous series of guests, which differ from one another only by a single substituent, and adopt the same geometry within the cavity of the macrocycle, is presented for the first time, and is used to decipher the competition between water and the carbonylated portal of CB[7] for the stabilization of positively charged guests. Binding affinities of CB[7] towards substituted *N*-benzyl-trimethylsilylmethylammonium cations relative to the unsubstituted member (X = H) range from 0.9 (X = CH_3_) to 3.1 (X = SO_2_CF_3_), and correlate very precisely with a linear combination of Swain–Lupton field/inductive (*F*; 67%) and resonance (*R*; 33%) parameters tabulated for each substituent. We show that this subtle sensitivity results exclusively from the balance between two competing mechanisms, on which the substituents exert an approximately 11 times greater impact: (1) the solvation of the ammonium unit and its immediate surroundings by water in the free guests, and (2) the coulombic attraction between the ammonium unit and the rim of CB[7] in the complexes.

## Abstract 2

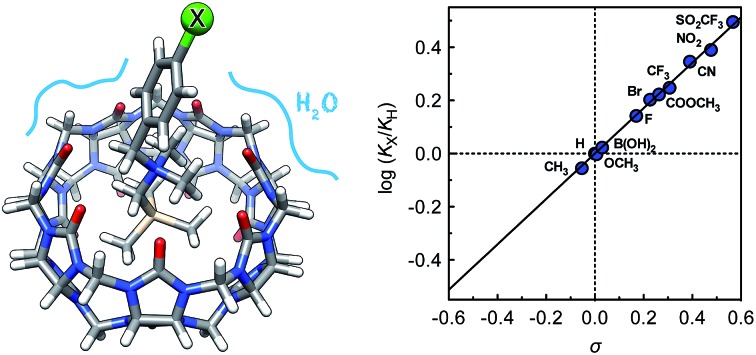
Remote substituents on cucurbit[7]uril-bound guests are used to decipher the fierce competition between water and the carbonylated portal of the macrocycle for guest stabilization.

## Introduction

**Chart 1:**
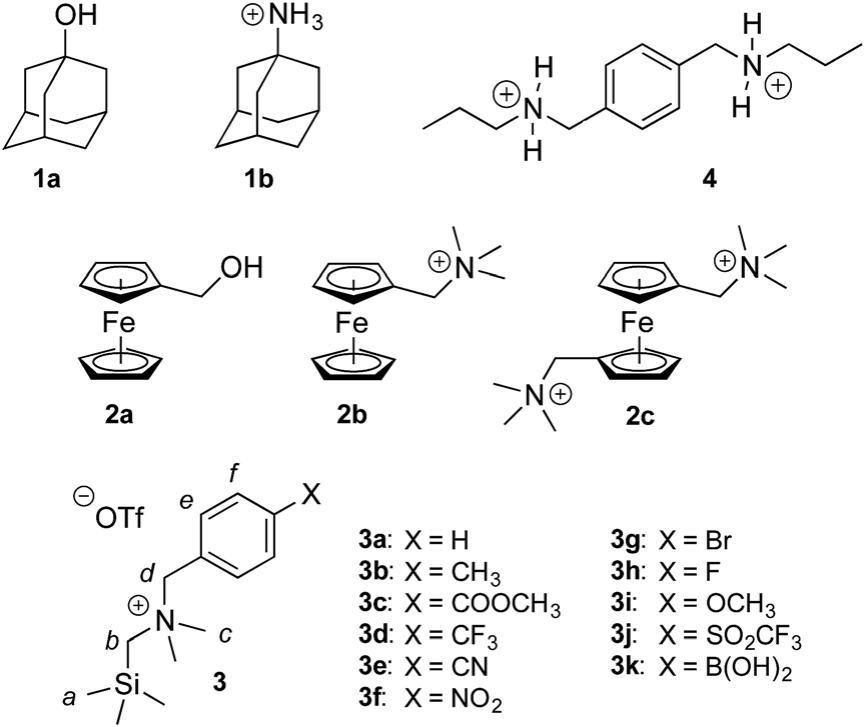
CB[7]-binding guests discussed in this study.

Cucurbiturils^1^–^3^ form notoriously tight complexes with organic guests, especially when the latter fit well within the cavity of the macrocycle. Optimal packing coefficients (*i.e.* the ratios of the volumes of the guest and of the host cavity) range from 50 to 60%,^4^ in agreement with Rebek's “55% solution”.^5^ In that case, nanomolar binding affinities are commonly measured for neutral species interacting with cucurbit[7]uril (CB[7]).^6^–^8^ The coulombic interaction between a positively charged substituent and one of the carbonylated portals of CB[*n*]s generally results in a 10^3^ to 10^4^-fold increase in binding affinity. For example, 1-adamantanol (**1a**) and 1-adamantylammonium (**1b**) display binding affinities of 2.3 × 10^10^ and 1.7 × 10^14^ M^–1^ towards CB[7] in water, respectively.^6^ Similarly, substituted ferrocenes **2a**, **2b** and **2c** bind increasingly tightly to CB[7] as one, then both CB[7] portals interact with a positively charged substituent (see Chart 1; affinities of 3.2 × 10^9^, 4.1 × 10^12^ and 3.0 × 10^15^ M^–1^, respectively).^6^ As a corollary, the p*K*_a_ of ammonium cations generally increases by 2–4 units upon CB[*n*] encapsulation, as the affinity of the corresponding neutral amine towards the macrocycle is 10^2^ to 10^4^-fold weaker than the ammonium cation.^9^–^13^ Yet, this 4–5 kcal mol^–1^ increase in binding affinity per CB/positive substituent interaction measured in aqueous solution pales in comparison to the corresponding gain in free energy in the gas phase,^6^ and the precise quantification of each contribution and penalty to the binding free energy in solution remains difficult. In the **2a–2c** series for example, the orientation of the ferrocene unit inside CB[7] is not steady, the magnitude or mere existence of hydrogen bonding between ferrocene methanol (**2a**) and the rim of the macrocycle is unclear, and the solvation energy of each guest is widely different. In this study, we circumvent these limitations by examining and rationalizing the binding affinities of CB[7] towards a homologous series of substituted *N*-benzyl-trimethylsilylmethylammonium cations (**3a–3k**; see Chart 1). In that case, the position of the trimethylsilyl unit inside the cavity of CB[7] and of the ammonium group at the rim remain steady throughout the series, and binding affinities are solely regulated by the electron-donating or withdrawing substituents at the remote *para*-position of the benzyl group and the accompanying solvation contributions.

## Results and discussion

**Fig. 1:**
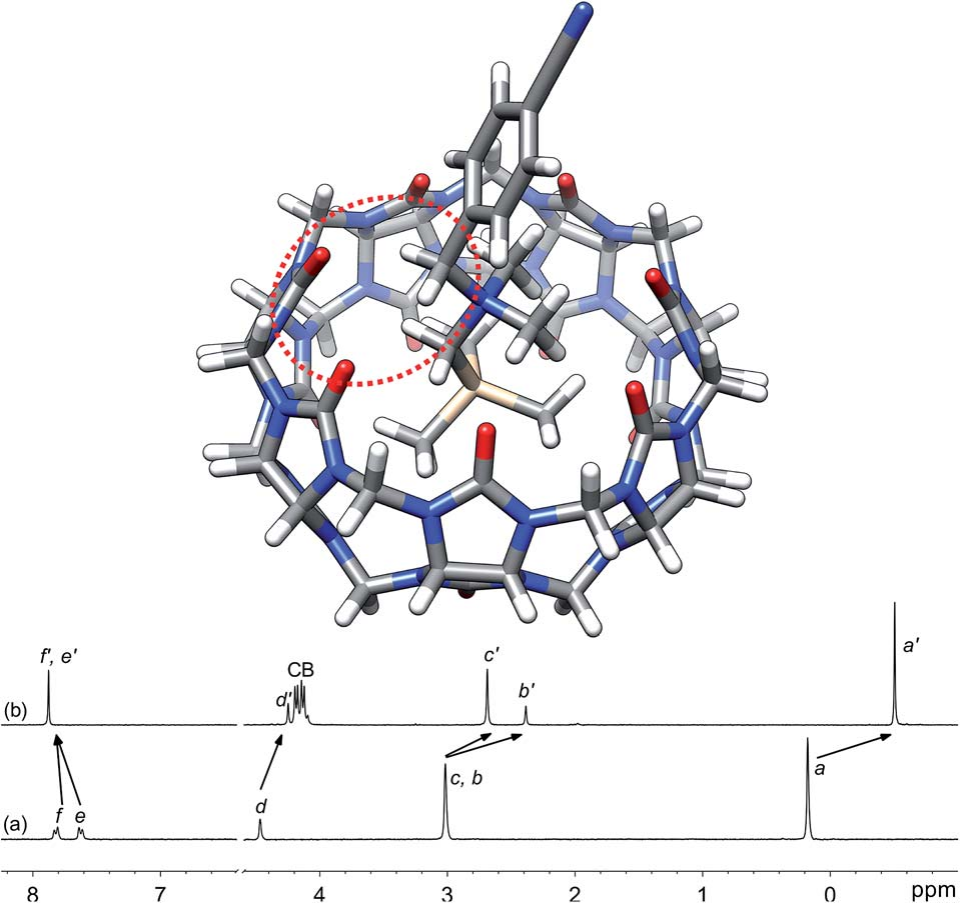
Optimized structure of complex **3e**·CB[7] calculated at the TPSS-D3(BJ)/def2-SVP level with the COSMO solvation model. The interaction between the benzylic hydrogens and the CB[7] rim is highlighted with the dotted red ellipse. ^1^H NMR spectra of (a) silane **3e** (X = CN), (b) complex **3e**·CB[7]. See Chart 1 for numbering.

Silanes **3a–3k** were prepared from *N*,*N*-dimethyl-(trimethylsilyl)methylamine and the corresponding benzyl halides in acetone, followed by anion exchange with silver or barium triflate. Upon interaction with CB[7], the ^1^H nuclear magnetic resonance (NMR) signals of the trimethylsilyl units undergo a large upfield shift (consistently 0.69 ppm throughout the series of guests **3**, thereby confirming their steady arrangement inside the cavity of the macrocycle; see Fig. 1, spectra a and b). They also show that the trimethylsilyl group quantitatively outcompetes the benzyl unit for CB[7] interaction (see Fig. 1 for an optimized structure of complex **3e**·CB[7]).

The relative binding affinities of silanes **3** towards CB[7] were determined by ^1^H NMR spectroscopy in a series of competition experiments using xylylene diammonium **4** as the reference guest; its CB[7] affinity is on par with silanes **3**, and its concentration as free and bound species was monitored using the signals of the two propyl tails (see ESI† section). The binding affinities of silanes **3** towards CB[7] relative to analog **3a** range from 0.9 (in the case of X = CH_3_) to 3.1 (X = SO_2_CF_3_). The absolute binding affinity of silane **3a** reached 1.5 × 10^12^ M^–1^, as determined by isothermal titration calorimetry (see ESI† section for the binding isotherms). The binding affinity was too high to be determined by direct titration, thus l-phenylalanine was used as a relay guest (*i.e.* the titration was carried out using silane **3a** and a 1 : 1 complex of CB[7] and l-phenylalanine; the binding affinity of the latter is 8.8 × 10^5^ M^–1^ in water).

**Fig. 2:**
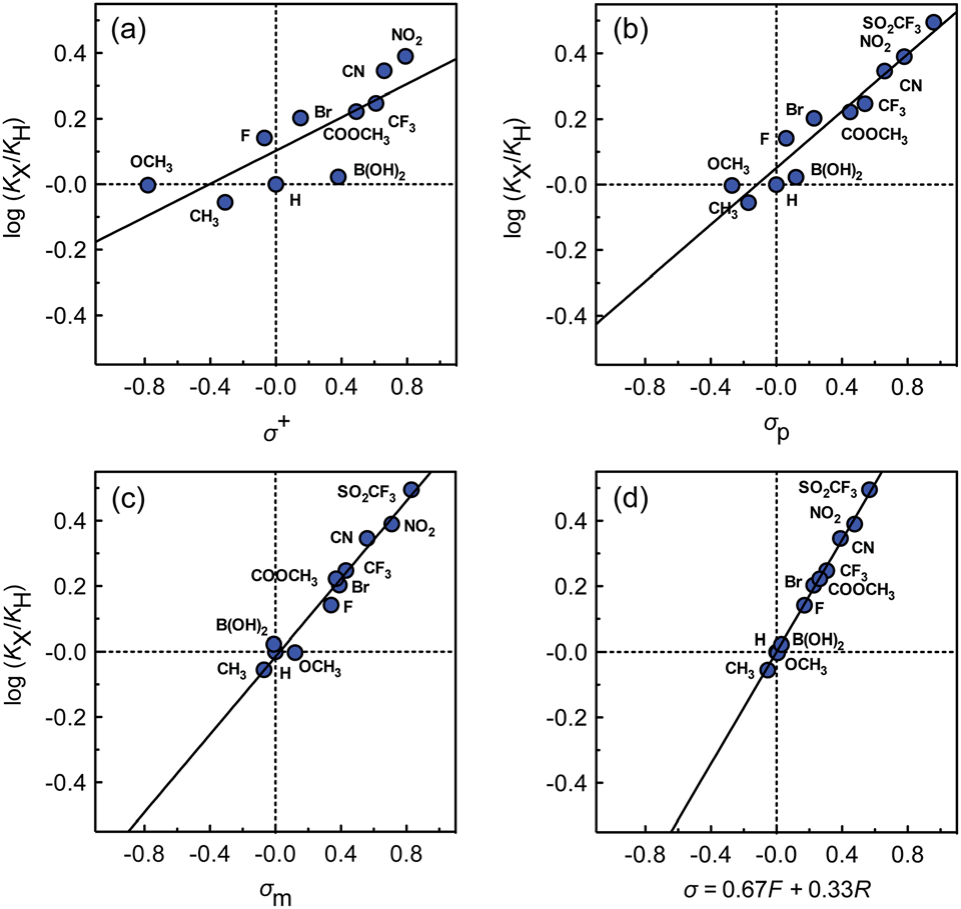
Binding affinities of silanes **3** (*K*_X_) relative to silane **3a** (*K*_H_) as a function of (a) Hammett parameters *σ*^+^, (b) Hammett parameters *σ*_p_, (c) Hammett parameters *σ*_m_, and (d) a linear combination of Swain–Lupton field/inductive (*F*) and resonance (*R*) parameters (*σ* = 0.67*F* + 0.33*R*).

The binding affinities of silanes **3** (*K*_X_) towards CB[7] relative to the unsubstituted member **3a** (*K*_H_) were plotted as a function of Hammett parameters *σ*^+^, *σ*_p_ and *σ*_m_ to assess the impact of the substituents on the affinities (see Fig. 2a–c).^14^ Hammett parameters reflect a combination of field, inductive and resonance substituent effects, with a bias towards field/induction in the case of *σ*_m_ and towards resonance for *σ*^+^, while both effects are evenly balanced in the case of *σ*_p_.^14^ For each of these parameters, coefficients of determination *r*^2^ were 0.646, 0.923 and 0.971, respectively. The fact that outliers are visibly present in each correlation indicates that both field (or induction) and resonance effects affect binding affinities, but not precisely in the ratios built into the *σ*_p_, *σ*_m_ and *σ*^+^ series of parameters. A near flawless linear relationship (*r*^2^ = 0.997; see Fig. 2d) could yet be obtained using a linear combination of Swain–Lupton field/inductive (*F*) and resonance (*R*) parameters that are derived from the Hammett parameters, and aim at treating both effects independently (see eqn (1); the *h* parameter accounts for all other effects);^14^,^15^
1
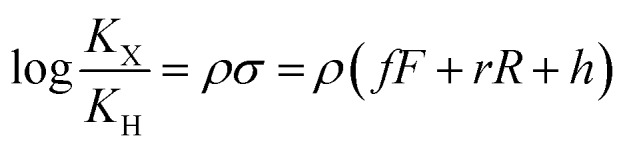

*f* and *r* are sensitivity factors (*f* + *r* = 1) that weigh field/induction and resonance effects, respectively; *ρ* is the overall sensitivity of the binding affinities to these parameters. Partial least squares regression analysis (PLS) afforded *f*, *r*, and *h* parameters equal to 0.67, 0.33 and –0.01, respectively. The residual contribution described by parameter *h* is thus insignificant, and can be neglected. The logarithmic plot of the relative binding affinities as a function of *σ* = 0.67*F* + 0.33*R* (see Fig. 2d) afforded a sensitivity factor *ρ* equal to 0.85 ± 0.01. PLS analysis carried out using Swain–Lupton *F* and *R* parameters as explanatory variables and Hammett parameters as dependent variables showed that the weights of the field/induction term built into the *σ*^+^, *σ*_p_ and *σ*_m_ series are 34%, 50% and 78%, respectively, based on the 11 substituents used in this study. Those contributions are indeed different than the 67% field/induction contribution to the CB[7] binding affinities calculated with the Swain–Lupton parameters, hence the less than optimal quality of the linear regressions obtained with Hammett parameters (Fig. 2a–c).

While we expected the binding affinities to be affected by field and induction effects, the magnitude of the resonance term (33%) is surprising, and indicates a pronounced interaction between the benzylic methylene group, whose electrostatic potential is affected by resonance through the aromatic ring, and the carbonylated rim of CB[7] (see Fig. 1, interaction highlighted in red).

That electron-withdrawing substituents would increase binding affinities by bolstering the density of positive charge at the ammonium center and the interaction with the CB[7] portal seems intuitive. A closer evaluation reveals otherwise: as the only difference between the members of the **3**·CB[7] complexes is a remote aryl substituent, differences in binding affinities are due to the changes in relative stabilization of the ammonium group by water and the CB[7] rim along the homologous series. Had ammonium solvation by water been more sensitive to substituent effects than CB[7] binding, electron-withdrawing groups would have *weakened* CB[7] binding! In order to decipher this competition between water and the CB[7] rim for ammonium interaction, we determined substituent effects (1) on the solvation of the free guests, (2) on the solvation of complexes **3**·CB[7], and (3) on the affinity of silanes **3** towards CB[7] in the gas phase.

The conformations of silanes **3** were screened using density functional theory (DFT) at the TPSS-D3(BJ)/def2-TZVP level.^16^,^17^ The “W-shaped” conformation as depicted in Fig. 1 was consistently the most stable one throughout the series of silanes **3**. Solvation energies Δ*G*_solv_(X) were then calculated with the COSMO^18^,^19^ and IEFPCM^20^–^22^ models. In order to limit the determination of the solvation to the ammonium unit (and the 4 surrounding methyl or methylene groups), we separate the solvation energy into 4 terms:

2 Δ*G*_solv_(X) = Δ*G*Sisolv + Δ*G*Nsolv(X) + Δ*G*Phsolv(X) + Δ*G*Corrsolv

where Δ*G*Sisolv, Δ*G*Nsolv(X) and Δ*G*Phsolv(X) are the free energies of solvation of trimethylsilane, the tetramethylammonium cation and benzene bearing a substituent X, respectively; Δ*G*Corrsolv is a substituent-independent correction factor. The solvation of the ammonium group, relative to the reference silane **3a** (X = H) is thus:

3 ΔΔ*G*Nsolv(X) = ΔΔ*G*_solv_(X) – ΔΔ*G*Phsolv(X)

where ΔΔ*G*_solv_(X) is the solvation energy of substituted silanes **3b–3k** relative to reference **3a**, and ΔΔ*G*Phsolv(X) is the solvation energy of *para*-substituted benzenes relative to benzene.

**Fig. 3:**
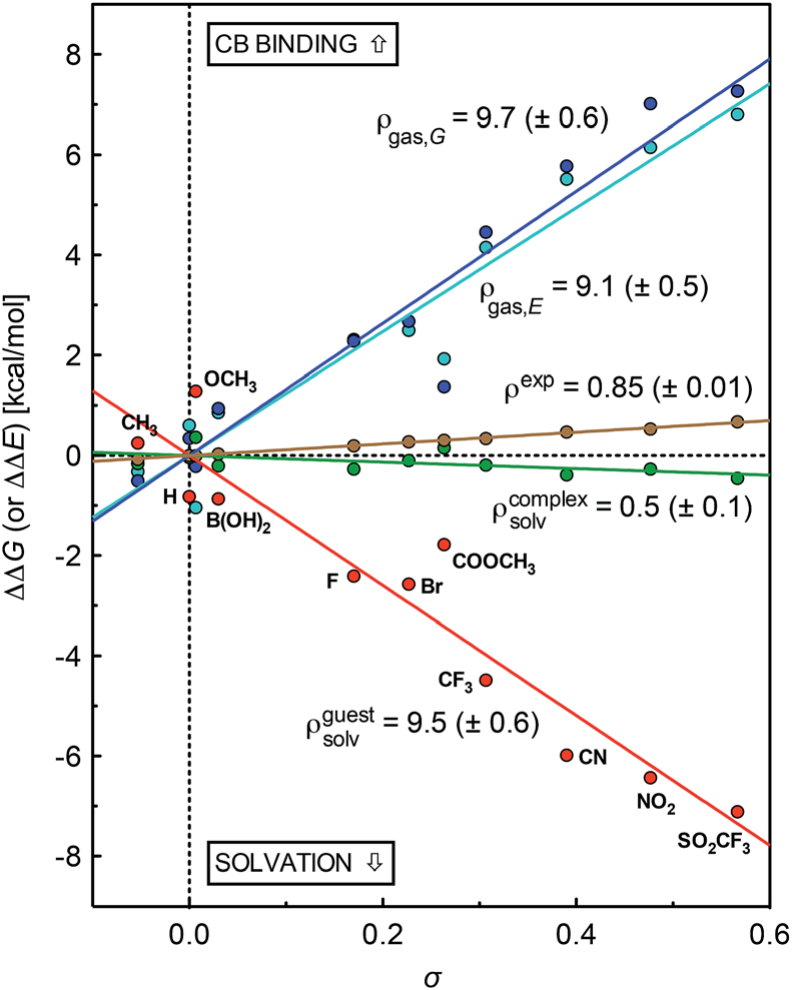
Free energy of binding for silanes **3** relative to silane **3a** (X = H), as determined by competitive NMR titrations (in brown). Relative solvation energies of silanes **3** (in red) and complexes **3**·CB[7] (in green); binding energy of silanes **3** in the gas phase (electronic contribution in cyan, free energy in blue, presented on a positive scale for better readability; –ΔΔ*E* and –ΔΔ*G*, respectively), as determined using DFT and the COSMO solvation model.

A plot of relative solvation energies of the ammonium unit as a function of the linear combination of Swain–Lupton parameters *σ* = 0.67*F* + 0.33*R* displays very good linearity, with a sensitivity factor *ρ*guestsolv of 9.5 ± 0.6 (see Fig. 3, red dots and regression line; the sensitivity factor is obtained from the slope of the regression line after dividing by 1.364 (*RT* ln 10) to convert relative energies into decimal logarithms of equilibrium constants). A very similar sensitivity factor was calculated using the IEFPCM solvation model and single-point energies calculated at the M05-2X/6-31G(d) level (*ρ*guestsolv = 9.2 ± 0.6).

The conformations of complexes **3**·CB[7] were then screened at the TPSS-D3(BJ)/def2-SVP level with the COSMO solvation model, and the total energies and solvation energies of the most stable conformers were obtained with def2-TZVP basis sets in single-point calculations (see ESI† for details; the guest adopts a “W-shaped” conformation throughout the series of silanes **3**, see Fig. 1). Solvation energies of the CB[7]-bound ammonium units relative to the reference complex **3a**·CB[7] were determined as described in eqn (3), and plotted as a function of parameter *σ* (see Fig. 3, green dots and regression line). Excellent linearity was again observed, but this time with a near-zero substituent sensitivity factor (*ρ*complexsolv = 0.5 ± 0.1). This indicates that (1) the carbonylated rim of CB[7] efficiently weakens the density of positive charge around the ammonium unit (and thereby lowers its solvation energy), (2) the field effect of the benzyl substituent does not propagate as far as the periphery of CB[7], and (3) surprisingly, CB[7] shields the ammonium group from virtually any water solvation. The binding affinity of CB[7] towards guests **3** were then calculated in the gas phase using the TPSS-D3(BJ)/def2-SVP-optimized structures discussed above, after single-point calculations with def2-TZVP basis sets. Enthalpic and entropic contributions were obtained after vibrational analysis at the TPSS-D3(BJ)/def2-SVP level, using Grimme's treatment for low vibrational frequencies (see ESI† for details).^23^

The gas phase affinities of guests **3** towards CB[7], relative to reference guest **3a**, were then plotted as a function of the linear combination of Swain–Lupton parameters *σ* = 0.67*F* + 0.33*R*. Very good linearity was obtained for both the electronic component of the binding affinity and the relative binding free energies after enthalpic and entropic corrections, albeit with a slightly larger error in the latter case (see cyan and blue dots with the corresponding regression lines, respectively); sensitivity to the benzyl substituents are *ρ*_gas,*E*_ = 9.1 ± 0.5 and *ρ*_gas,*G*_ = 9.7 ± 0.6.

The sensitivity of binding affinities to substituents calculated in the gas phase is thus approximately 11 times greater than the one measured in aqueous solution. This is reminiscent of the 6.6-fold difference obtained by Taft and coworkers when comparing the gas and aqueous phase acidities of *meta*- and *para*-substituted phenols.^24^

Sensitivity factors pertaining to solvation and CB[7] binding are strikingly similar, and highlight the fierce competition between water and the rim of CB[7] for ammonium binding. The cumulative sensitivity factor *ρ*_calc_ can be calculated using eqn (4), and is equal to 0.6 (±0.9), in excellent agreement with the sensitivity determined experimentally (*ρ* = 0.85 ± 0.01).

4 *ρ*_calc_ = *ρ*_gas,*G*_ – (*ρ*guestsolv – *ρ*complexsolv)

We also note that while DFT calculations accurately predict the trend in binding affinities along the series of silanes **3**, they fail to predict accurate absolute free energies of binding. Whereas a free energy of –16.9 kcal mol^–1^ is determined for CB[7] binding to reference silane **3a** experimentally, calculations greatly underestimate the free energy and return –3.7 kcal mol^–1^ with the COSMO solvation model, and –9.5 kcal mol^–1^ with the IEFPCM model. In fact, we find this negative result rather reassuring: as shown by Nau, Biedermann and coworkers^4^,^25^–^27^ the ejection of high energy water from the cavity of CB[*n*]s is the main driving force of the binding event, and continuum solvation models like COSMO or IEFPCM are expected to overestimate the solvation energy of the empty macrocycle. However, this result contrasts with the more accurately computed binding affinities obtained by Inoue and Gilson^6^ (±4 kcal mol^–1^), as well as Grimme and coworkers^28^ (±2 kcal mol^–1^) using continuum solvation models. Yet, in the latter case, the authors compared affinities calculated in water with affinities determined experimentally in a 0.10 M sodium phosphate buffer adjusted to pH 7.4.^29^ The high concentration of sodium cations (0.30 M) competing for CB[7] binding is expected to lower the affinities of the guests by 200 to 1000-fold compared to those in pure water.^30^ Therefore, calculations underestimate binding affinities by an additional 3–4 kcal mol^–1^ bias, which the authors have not taken into account. In the present study, it is not currently possible for us to assess which portion of the 7–13 kcal mol^–1^ discrepancy between calculated and experimental free energies is due to the ejection of high-energy water from the cavity, and to the error caused by our computational choices.

Finally, we wanted to test whether enthalpy or entropy variations were mainly responsible for the increase in CB[7] binding affinity along the series of silanes **3**. Kaifer, Isaacs, Kim, Inoue and Gilson^6^,^8^ show that an increase in solvation entropy is responsible for the improved binding affinities measured along the series of guests **1** and **2** (see Chart 1). We had already determined the thermodynamic parameters for the interaction between CB[7] and guest **3a**, therefore we carried out another series of titrations with guest **3f** (X = NO_2_). Binding affinities were 1.5 (±0.1) × 10^12^ and 3.2 (±0.2) × 10^12^ M^–1^, respectively. This result is in excellent agreement with the 2.5-fold difference between the two guests measured by competitive NMR titrations. Although the difference in binding affinities is small, the high quality of the ITC titration fitting allows a very accurate evaluation of the enthalpic and free energy parameters (–15.45 (±0.03) and –16.63 (±0.02) kcal mol^–1^ in the case of silane **3a**; –15.45 (±0.03) and –17.07 (±0.03) kcal mol^–1^ for silane **3f**). As the enthalpic terms are identical for both silanes, the difference in binding affinity is again solely due to the entropic term (*T*Δ*S* = 1.43 (±0.04) and 1.87 (±0.04) kcal mol^–1^, respectively), in agreement with the studies mentioned above.

## Conclusions

Exploiting substituent effects in a quantitative manner is a classic method available in the physical organic chemist toolbox to study reaction mechanisms, yet to the best of our knowledge this is the first time it has been used to decipher the various forces at play in CB[*n*]/guests interactions. By varying a remote *para*-substituent in a series of *N*-benzyl-trimethylsilylmethylammonium cations **3**, and thereby leaving the trimethylsilyl CB[*n*]-binding unit in a steady position inside the cavity of the macrocycle throughout the series, the role of the ammonium unit on the binding process, and how water solvation and interactions with the carbonylated CB[7] rim affected it, could be treated separately from the rest of the structure. We showed that the mild impact of the substituent on binding affinities in water is essentially due to a barely tilted balance between two competing mechanisms that are affected by substituent changes to a much greater extent, by approximately 11-fold compared to the combined effect: (1) the solvation of the ammonium unit and its immediate surroundings by water in the free guests, and (2) the coulombic attraction between the ammonium unit and the CB[7] portal in the complexes. The solvation of the complexes is barely affected by substituents, and does not play a major role in the competition, as the CB[7] rim annihilates most of the positive charge around the ammonium unit, and the macrocycle seems to shield the ammonium group from most water solvation. Beyond these fundamental aspects of CB[*n*] recognition, this study is also intended as a guide to fine-tune the binding affinities of guests in CB[*n*]-based self-assembling systems.

## Supplementary Material

Supplementary information
