# Supplementary material for: Water vs. cucurbituril rim: a fierce competition for guest solvation
Source: Chem Sci. 2016 Feb 17;7(6):3569–73. doi: 10.1039/c5sc04475h (PMC6007185; doi:10.1039/c5sc04475h)
Supplement: Supplementary file 1 [file SC-007-C5SC04475H-s001.pdf]

Electronic Supplementary Material (ESI) for Chemical Science.
This journal is © The Royal Society of Chemistry 2016

Water vs Cucurbituril rim: a fierce competition for guest solvation

Xiaoxi Ling, Stefan Saretz, Lifeng Xiao, John Francescon and Eric Masson*

[a] *Department of Chemistry and Biochemistry, Ohio University, Athens, OH 45701, USA*

e-mail: masson@ohio.edu

Supporting Information

1. Generalities 2
2. Preparation of precursors 2
3. Preparation of silanes **3a** – **3k** 3
4. Determination of the binding affinity of CB[7] towards silanes **3** using competitive NMR titrations 8
5. Isothermal Titration Calorimetry experiments 10
6. ^{1}H and ^{13}C NMR spectra of silanes **3a** – **3k** 12
7. Computational details 45
8. Computational data 47
9. Optimized structure and coordinates of complex **3a**·CB[7] 50
10. Optimized structure and coordinates of complex **3e**·CB[7] 51
11. References 52

1. Generalities

Starting materials were purchased from Sigma-Aldrich (St. Louis, MO), TCI America (Portland, OR) and Cambridge Isotope Laboratories (Andover, MA). Cucurbit[7]uril (CB[7]) was prepared using known procedures.[1] Characterization by nuclear magnetic resonance spectroscopy (NMR) was carried out using a Bruker 300 spectrometer (Billerica, MA). Products were also characterized by high-resolution mass spectrometry (HRMS) performed at the COSMIC facility of the Old Dominion University (Norfolk, VA) using a Bruker Daltonics 12 Tesla APEX-Qe FTICR mass spectrometer with an Apollo II Ion Funnel. ITC experiments were performed using an iTC200 calorimeter (Microcal Inc., GE Healthcare, Piscataway, NJ). All computational jobs were run on the Oakley cluster (HP-built, Intel® Xeon® processors; 8,328 cores, 694 nodes) at the Ohio Supercomputer Center (Columbus, OH). Optimized structures were visualized using GaussView 5.0 (SemiChem, Inc.; Wallingford, CT) and Chimera (UCSF).

2. Preparation of precursors

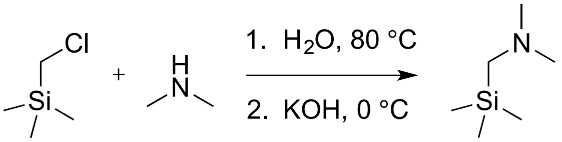

***N*,*N*-dimethyl-1-(trimethylsilyl)methanamine.**[2, 3] A heterogeneous mixture of (chloromethyl)trimethylsilane (1.5 g, 12 mmol) and aqueous dimethylamine solution (40%, 9.3 g, 82 mmol) was sealed in a pressured vessel and heated at 80 ˚C for 20 h. The reaction mixture was cooled to 0 ˚C, and potassium hydroxide pellets were added (2.0 g, 30 mmol) before extraction with a mixture of pentane (20 mL) and diethyl ether (20 mL). The organic layer was washed with brine and carefully evaporated (600 mbar, 30 ˚C) to afford the title compound as a clear volatile oil (1.3 g, 81%). The product was used in the next step without further purification. A small sample of the amine was acidified with hydrochloric acid to yield a white solid used for spectroscopic purposes. ^{1}H NMR (D_2O, chemical shifts δ refer to the signal of dioxane (δ = 3.75 ppm) as external reference): δ 2.86 (s, $N^+(CH_3)_2$, 6 H), 2.75 (s, N^+CH_2, 2 H), 0.16 (s, $Si(CH_3)_3$, 9 H) ppm. ^{13}C NMR: δ 51.0, 47.2, –2.4 ppm.

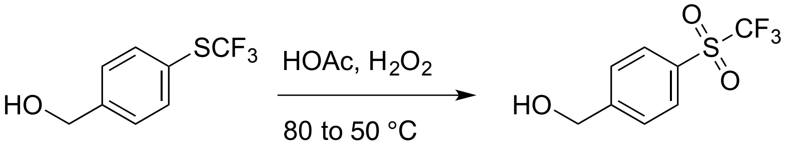

(4-(trifluoromethylsulfonyl)phenyl)methanol.[4] A solution of (4-(trifluoromethylthio)-phenyl)methanol (1.0 g, 4.8 mmol) in acetic acid (12 mL) was mixed with hydrogen peroxide (30%, 5.5 g, 48 mmol) and stirred at 80 ˚C for 10 min and then at 50 ˚C for 48 h. The resulting mixture was poured into water (20 mL) and extracted with diethyl ether

(3 × 30 mL). The combined organic layers were washed with a 10% sodium bicarbonate solution (20 mL), brine (20 mL), dried over sodium sulfate and concentrated *in vacuo*. The residue was subjected to flash chromatography (eluent: ethyl acetate/hexanes = 1 : 2) to afford the title compound as a white solid (0.50 g, 43%); m.p. 52 – 53 ˚C. ^{1}H NMR (DMSO-d^6): δ 8.10 (d, J = 8.3 Hz, 2 × ArH, 2 H), 7.78 (d, J = 8.5 Hz, 2 × ArH, 2 H), 5.63 (br s, OH, 1 H), 4.70 (s, ArCH_2, 2 H) ppm. ^{13}C NMR: δ 153.8, 130.7, 127.9, 127.2, 119.4 (q, J = 326 Hz), 62.0 ppm.

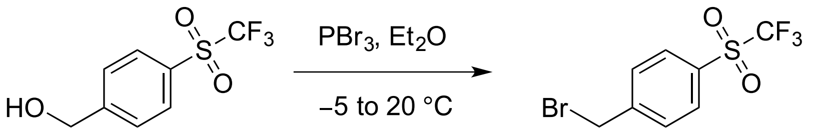

1-(bromomethyl)-4-(trifluoromethylsulfonyl)benzene. Phosphorous tribromide (0.11 g, 0.42 mmol) was added to a solution of benzyl alcohol (4-(trifluoromethylsulfonyl)-phenyl)methanol (0.10 g, 0.42 mmol) in ether (2.5 mL) at –5 ˚C. The resulting mixture was stirred at –5 ˚C for 5 h and then at 20 ˚C for 20 h. The mixture was poured into an ice/water mixture (10 mL) and extracted with diethyl ether (3 × 20 mL). The combined extracts were washed with a saturated sodium bicarbonate solution (20 mL) and brine (20 mL), then dried with sodium sulfate and evaporated to afford the title compound as a clear oil that solidifies over time (85 mg, 67%). The product was used in the next step without further purification. m.p. 46 – 47 ˚C (lit.[5] 46 ˚C) ^{1}H NMR (DMSO-d^6): δ 8.16 (d, J = 8.3 Hz, 2 × ArH, 2 H), 7.91 (d, J = 8.5 Hz, 2 × ArH, 2 H), 4.87 (s, ArCH_2, 2 H) ppm.

3. Preparation of silanes 3a – 3k

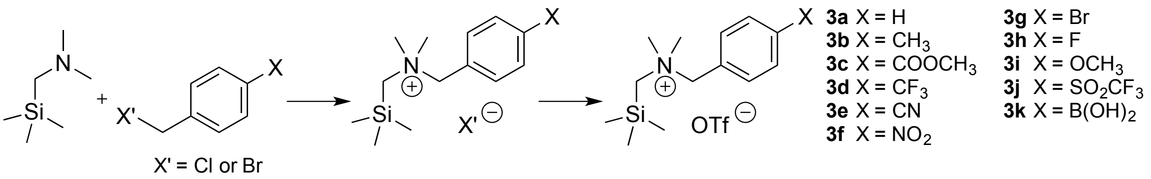

General procedure for benzyl ammonium trifluoromethanesulfonates ***3a** – **3k***

The benzyl halide (1.5 mmol) was added to a solution of *N*,*N*-dimethyl-1-(trimethylsilyl)methanamine (0.20 g, 1.5 mmol) in acetone (5.0 mL). The mixture was heated to reflux at 60 ˚C for 20 h. Upon cooling, the mixture was triturated with diethyl ether to give a white precipitate that was collected by filtration, and washed with diethyl ether. The resulting solid was subsequently dissolved in methanol (1.0 mL) and mixed with silver trifluoromethanesulfonate (1.5 mmol) as a 0.16 M methanolic solution. The precipitate was removed by filtration, and the filtrate evaporated. The residue was triturated with diethyl ether. The precipitate was collected by filtration, washed with diethyl ether, and dried under vacuum to afford an amorphous white powder.

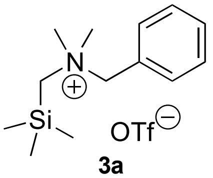

3a

***N*-benzyl-*N*,*N*-dimethyl-1-(trimethylsilyl)methanaminium trifluoromethanesulfonate (3a).** Yield: 46%. m.p. 120 – 121 °C. ^{1}H NMR (D_2O): δ 7.47 (m, 5 × Ar*H*, 5 H), 4.40 (s, ArCH_2, 2 H), 3.00 (s, $(CH_3)_2N^+CH_2Si$, 8 H), 0.19 (s, Si$(CH_3)_3$, 9 H) ppm. ^{1}H NMR ($CDCl_3$): δ 7.39 (m, 5 × Ar*H*, 5 H), 4.54 (s, ArCH_2, 2 H), 3.06 (s, N^+CH_2Si, 2 H), 3.03 (s, $(CH_3)_2N^+$, 6 H), 0.19 (s, Si$(CH_3)_3$, 9 H) ppm. ^{13}C NMR ($CDCl_3$): δ 133.3, 131.0, 129.4, 127.7, 120.9 (q, *J* = 320 Hz), 72.6, 58.4, 52.7, –0.5 ppm. HRMS (ESI) *m/z* calcd for $C_{13}H_{24}NSi$ ($[M–OTf]^+$) 222.167253, found 222.167327.

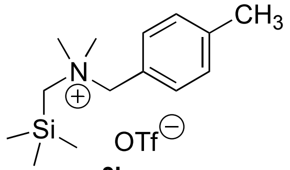

3b

***N*,*N*-dimethyl-*N*-(4-methylbenzyl)-1-(trimethylsilyl)methanaminium trifluoromethanesulfonate (3b).** Yield: 52%. m.p. 88 – 90 °C. ^{1}H NMR (D_2O): δ 7.34 (t, *J* = 8.0 Hz, 2 × Ar*H*, 2 H), 7.31 (t, *J* = 8.0 Hz, 2 × Ar*H*, 2 H), 4.35 (s, ArCH_2, 2 H), 2.97 (s, $(CH_3)_2N^+CH_2Si$, 8 H), 2.31 (s, ArCH_3, 3 H), 0.18 (s, Si$(CH_3)_3$, 9 H) ppm. ^{1}H NMR ($CDCl_3$): δ 7.31 (d, *J* = 8.0 Hz, 2 × Ar*H*, 2 H), 7.14 (d, *J* = 7.8 Hz, 2 × Ar*H*, 2 H), 4.47 (s, ArCH_2, 2 H), 3.03 (s, N^+CH_2Si, 2 H), 3.00 (s, $(CH_3)_2N^+$, 6 H), 2.29 (s, ArCH_3, 3 H), 0.18 (s, Si$(CH_3)_3$, 9 H) ppm. ^{13}C NMR ($CDCl_3$): δ 141.3, 133.1, 130.0, 124.6, 120.8 (q, *J* = 320 Hz), 72.5, 58.1, 52.6, 21.4, –0.5 ppm. HRMS (ESI) *m/z* calcd for $C_{14}H_{26}NSi$ ($[M–OTf]^+$) 236.182903, found 236.182952.

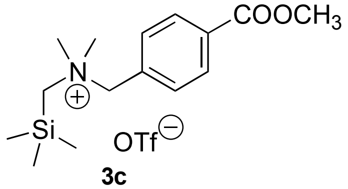

3c

***N*-(4-(methoxycarbonyl)benzyl)-*N*,*N*-dimethyl-1-(trimethylsilyl)methanaminium trifluoromethanesulfonate (3c).** Yield: 48%. m.p. 70 – 72 °C. ^{1}H NMR (D_2O): δ 8.06 (d, *J* = 8.1 Hz, 2 × Ar*H*, 2 H), 7.60 (d, *J* = 8.1 Hz, 2 × Ar*H*, 2 H), 4.47 (s, ArCH_2, 2 H), 3.88 (s, COOCH_3, 3 H), 3.03 (s, $(CH_3)_2N^+CH_2Si$, 8 H), 0.19 (s, Si$(CH_3)_3$, 9 H) ppm. ^{1}H NMR ($CDCl_3$): δ 7.97 (d, *J* = 8.0 Hz, 2 × Ar*H*, 2 H), 7.56 (d, *J* = 8.1 Hz, 2 × Ar*H*, 2 H), 4.65 (s, ArCH_2, 2 H), 3.86 (s, COOCH_3, 3 H), 3.05 (s, $(CH_3)_2N^+CH_2Si$, 8 H), 0.18 (s, Si$(CH_3)_3$, 9 H) ppm. ^{13}C NMR ($CDCl_3$): δ 166.2, 133.4, 132.5, 132.2, 130.3, 120.8 (q, *J* = 320 Hz), 71.8, 58.5, 52.9, 52.6, –0.5 ppm. HRMS (ESI) *m/z* calcd for $C_{15}H_{26}NO_2Si$ ($[M–OTf]^+$) 280.172732, found 280.172760.

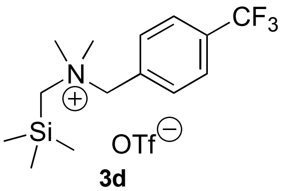

3d

***N*,*N*-dimethyl-*N*-(4-(trifluoromethyl)benzyl)-1-(trimethylsilyl)methanaminium trifluoromethanesulfonate (3d).** Yield: 40%. m.p. 96 – 98 ˚C. ^{1}H NMR (D_2O): δ 7.79 (d, *J* = 8.2 Hz, 2 × Ar*H*, 2 H), 7.65 (d, *J* = 7.9 Hz, 2 × Ar*H*, 2 H), 4.49 (s, ArC*H*$_2$, 2 H), 3.03 (s, $(CH_3)_2N^+CH_2Si$, 8 H), 0.20 (s, $Si(CH_3)_3$, 9 H) ppm. ^{1}H NMR (DMSO-*d*^{6}): δ 7.91 (d, *J* = 8.1 Hz, 2 × Ar*H*, 2 H), 7.81 (d, *J* = 8.1 Hz, 2 × Ar*H*, 2 H), 4.60 (s, ArC*H*$_2$, 2 H), 3.09 (s, N^+CH_2Si, 2 H), 3.04 (s, $(CH_3)_2N^+$, 6 H), 0.24 (s, $Si(CH_3)_3$, 9 H) ppm. ^{13}C NMR (DMSO-*d*^{6}): δ 134.0, 133.0, 130.5 (q, *J* = 32 Hz), 125.7 (m), 122.18, 120.7 (q, *J* = 322 Hz), 69.9, 57.8, 52.8, –0.9 ppm. HRMS (ESI) *m/z* calcd for $C_{14}H_{23}F_3NSi$ ([M–OTf]$^+$) 290.154637, found 290.154650.

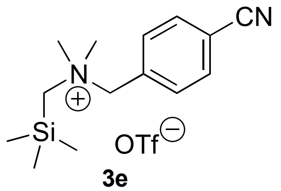

3e

***N*-(4-cyanobenzyl)-*N*,*N*-dimethyl-1-(trimethylsilyl)methanaminium trifluoromethanesulfonate (3e).** Yield: 50%. m.p. 140 – 141 ˚C. ^{1}H NMR (D_2O): δ 7.84 (d, *J* = 8.3 Hz, 2 × Ar*H*, 2 H), 7.64 (d, *J* = 8.2 Hz, 2 × Ar*H*, 2 H), 4.49 (s, ArC*H*$_2$, 2 H), 3.04 (s, $(CH_3)_2N^+CH_2Si$, 8 H), 0.20 (s, $Si(CH_3)_3$, 9 H) ppm. ^{1}H NMR (DMSO-*d*^{6}): δ 8.02 (d, *J* = 8.2 Hz, 2 × Ar*H*, 2 H), 7.78 (d, J = 8.3 Hz, 2 × Ar*H*, 2 H), 4.57 (s, ArC*H*$_2$, 2 H), 3.08 (s, N^+CH_2Si, 2 H), 3.03 (s, $(CH_3)_2N^+$, 6 H), 0.23 (s, $Si(CH_3)_3$, 9 H) ppm. ^{13}C NMR (DMSO-*d*^{6}): δ 134.0, 133.6, 132.7, 118.5, 116.0 (q, *J* = 344 Hz), 113.05, 70.0, 58.0, 52.80, –0.9 ppm. HRMS (ESI) *m/z* calcd for $C_{14}H_{23}N_2Si$ ([M–OTf]$^+$) 247.162502, found 247.162564.

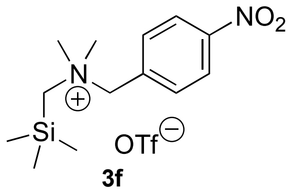

3f

***N*,*N*-dimethyl-*N*-(4-nitrobenzyl)-1-(trimethylsilyl)methanaminium trifluoromethanesulfonate (3f).** Yield: 48%. m.p. 143 – 145 ˚C. ^{1}H NMR (D_2O): δ 8.29 (d, *J* = 8.5 Hz, 2 × Ar*H*, 2 H), 7.72 (d, *J* = 8.7 Hz, 2 × Ar*H*, 2 H), 4.54 (s, ArC*H*$_2$, 2 H), 3.06 (s, $(CH_3)_2N^+CH_2Si$, 8 H), 0.20 (s, $Si(CH_3)_3$, 9 H) ppm. ^{1}H NMR (DMSO-*d*^{6}): δ 8.38 (d, *J* = 8.5 Hz, 2 × Ar*H*, 2 H), 7.88 (d, J = 8.5 Hz, 2 × Ar*H*, 2 H), 4.65 (s, ArC*H*$_2$, 2 H),

3.10 (s, N^+CH_2Si, 2 H), 3.06 (s, $(CH_3)_2N^+$, 6 H), 0.25 (s, $Si(CH_3)_3$, 9 H) ppm. ^{13}C NMR (DMSO-d^6): δ 148.6, 135.4, 134.6, 123.7, 120.7 (q, J = 325 Hz), 69.5, 58.0, 52.8, –0.9 ppm. HRMS (ESI) *m/z* calcd for $C_{13}H_{23}N_2O_2Si$ ($[M–OTf]^+$) 267.152331, found 267.152364.

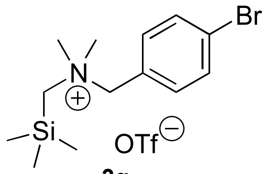

3g

***N*-(4-bromobenzyl)-*N*,*N*-dimethyl-1-(trimethylsilyl)methanaminium trifluoromethanesulfonate (3g).** Yield: 36%. m.p. 116 – 118 ˚C. ^1H NMR (D_2O): δ 7.64 (d, J = 8.3 Hz, 2 × Ar*H*, 2 H), 7.37 (d, J = 8.3 Hz, 2 × Ar*H*, 2 H), 4.37 (s, ArCH_2, 2 H), 3.00 (s, $(CH_3)_2N^+CH_2Si$, 8 H), 0.19 (s, $Si(CH_3)_3$, 9 H) ppm. ^1H NMR ($CDCl_3$): δ 7.48 (d, J = 8.4 Hz, 2 × Ar*H*, 2 H), 7.35 (d, J = 8.4 Hz, 2 × Ar*H*, 2 H), 4.56 (s, ArCH_2, 2 H), 3.02 (s, $(CH_3)_2N^+CH_2Si$, 8 H), 0.18 (s, $Si(CH_3)_3$, 9 H) ppm. ^{13}C NMR ($CDCl_3$): δ 134.9, 132.7, 126.6, 125.9, 120.8 (q, J = 320 Hz), 71.8, 58.2, 52.7, –0.5 ppm. HRMS (ESI) *m/z* calcd for $C_{13}H_{23}BrNSi$ ($[M–OTf]^+$) 300.077765, found 300.077838.

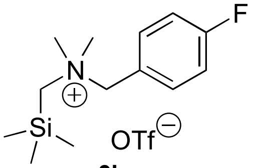

3h

***N*-(4-fluorobenzyl)-*N*,*N*-dimethyl-1-(trimethylsilyl)methanaminium trifluoromethanesulfonate (3h).** Yield: 63%. m.p. 155 – 156 ˚C. ^1H NMR (D_2O): δ 7.49 (dd, J = 8.4, 5.4 Hz, 2 × Ar*H*, 2 H), 7.19 (dd, J = 8.8, 8.8 Hz, 2 × Ar*H*, 2 H), 4.39 (s, ArCH_2, 2 H), 2.99 (s, $(CH_3)_2N^+CH_2Si$, 8 H), 0.19 (s, $Si(CH_3)_3$, 9 H) ppm. ^1H NMR (DMSO-d^6): δ 7.63 (dd, J = 8.6, 5.5 Hz, 2 × Ar*H*, 2 H), 7.37 (dd, J = 8.8, 8.8 Hz, 2 × Ar*H*, 2 H), 4.49 (s, ArCH_2, 2 H), 3.05 (s, N^+CH_2Si, 2 H), 2.99 (s, $(CH_3)_2N^+$, 6 H), 0.24 (s, $Si(CH_3)_3$, 9 H) ppm. ^{13}C NMR (DMSO-d^6): δ 163.3 (d, J = 249 Hz), 135.4 (d, J = 9 Hz), 124.92 (d, J = 3 Hz), 120.7 (q, J = 322 Hz), 115.9 (d, J = 21 Hz), 70.0, 57.5, 52.4, –0.9 ppm. HRMS (ESI) *m/z* calcd for $C_{13}H_{23}FNSi$ ($[M–OTf]^+$) 240.157831, found 240.157896.

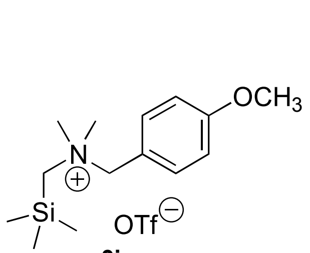

3i

N-(4-methoxybenzyl)-N,N-dimethyl-1-(trimethylsilyl)methanaminium trifluoromethanesulfonate (3i). Yield: 51%. m.p. 104 – 106 °C. ^{1}H NMR (D_2O): δ 7.41 (d, J = 8.6 Hz, 2 × ArH, 2 H), 7.02 (d, J = 8.6 Hz, 2 × ArH, 2 H), 4.33 (s, ArCH_2, 2 H), 3.80 (s, OCH_3, 3 H), 2.96 (s, $(CH_3)_2N^+CH_2Si$, 8 H), 0.18 (s, $Si(CH_3)_3$, 9 H) ppm. ^{1}H NMR ($CDCl_3$): δ 7.38 (d, J = 8.5 Hz, 2 × ArH, 2 H), 6.84 (d, J = 8.4 Hz, 2 × ArH, 2 H), 4.50 (s, ArCH_2, 2 H), 3.74 (s, OCH_3, 3 H), 3.01 (s, $(CH_3)_2N^+CH_2Si$, 8 H), 0.19 (s, $Si(CH_3)_3$, 9 H) ppm. ^{13}C NMR ($CDCl_3$): δ 161.6, 134.7, 120.9 (q, J = 320 Hz), 119.5, 114.7, 72.4, 57.9, 55.5, 52.5, –0.4 ppm. HRMS (ESI) m/z calcd for $C_{14}H_{26}NOSi$ ([M–OTf]$^+$) 252.177817, found 252.177853.

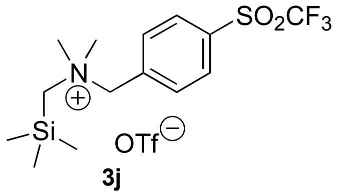

3j

N,N-dimethyl-N-(4-(trifluoromethylsulfonyl)benzyl)-1-(trimethylsilyl)methanaminium trifluoromethanesulfonate (3j). Yield: 50%. m.p. 220 – 221 °C. ^{1}H NMR (D_2O): δ 8.19 (d, J = 8.2 Hz, 2 × ArH, 2 H), 7.88 (d, J = 8.4 Hz, 2 × ArH, 2 H), 4.58 (s, ArCH_2, 2 H), 3.06 (s, $(CH_3)_2N^+CH_2Si$, 8 H), 0.20 (s, $Si(CH_3)_3$, 9 H) ppm. ^{1}H NMR (DMSO-d^6): δ 8.31 (d, J = 8.2 Hz, 2 × ArH, 2 H), 8.04 (d, J = 8.4 Hz, 2 × ArH, 2 H), 4.68 (s, ArCH_2, 2 H), 3.13 (s, N^+CH_2Si, 2 H), 3.07 (s, $(CH_3)_2N^+$, 6 H), 0.24 (s, $Si(CH_3)_3$, 9 H) ppm. ^{13}C NMR (DMSO-d^6): δ 138.1, 135.3, 131.2, 131.1, 120.7 (q, J = 322 Hz), 119.4 (q, J = 326 Hz), 69.4, 58.2, 53.0, –0.9 ppm. HRMS (ESI) m/z calcd for $C_{14}H_{23}F_3NO_2SSi$ ([M–OTf]$^+$) 354.116537, found 354.116617.

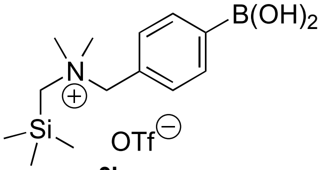

3k

N-(4-boronobenzyl)-N,N-dimethyl-1-(trimethylsilyl)methanaminium trifluoromethanesulfonate (3k). The ion exchange was performed with barium trifluoromethanesulfonate instead of the corresponding silver salt in acetonitrile. The barium bromide precipitate was removed by filtration. The filtrate was evaporated and triturated with diethyl ether, and the precipitate was collected by filtration, washed with chloroform, and dried under vacuum. Yield: 39%. m.p. 114 – 115 °C. ^{1}H NMR (D_2O): δ 7.78 (d, J = 7.8 Hz, *2* × ArH, 2 H), 7.48 (d, J = 7.9 Hz, *2* × ArH, 2 H), 4.41 (s, ArCH_2, 2 H), 3.00 (s, $(CH_3)_2N^+CH_2Si$, 8 H), 0.19 (s, $Si(CH_3)_3$, 9 H) ppm.^{1}H NMR (DMSO-d^6): δ 8.22 (s, B(OH)$_2$, 2 H), 7.91 (d, J = 7.5 Hz, 2 × ArH, 2 H), 7.53 (d, J = 7.6 Hz, 2 × ArH, 2 H), 4.49 (s, ArCH_2, 2 H), 3.07 (s, N^+CH_2Si, 2 H), 3.00 (s, $(CH_3)_2N^+$, 6 H), 0.24 (s, $Si(CH_3)_3$, 9 H). ^{13}C NMR (DMSO-d^6): δ 134.4, 132.1, 130.1, 118.7 (q, J = 317 Hz), 70.8, 57.8, 52.6, –0.8 ppm. HRMS (ESI) m/z calcd for $C_{13}H_{25}BNO_2Si$ ([M–OTf]$^+$) 266.174213, found 266.174232.

4. Determination of the binding affinity of CB[7] towards silanes 3 using competitive NMR titrations

Affinities of CB[7] towards silanes **3** relative to xylylene diammonium **4** were determined using ^{1}H NMR in deuterium oxide, by carrying out competitive equilibrations in the presence of a fixed concentration of guest **4** (2.0 mM) and various amounts of silanes **3** (see equation below), and averaging affinities for each guest. The concentrations of free and CB[7]-bound guests were calculated using the integration of the trimethylsilyl signals of guest **3** (see signals marked with circles in Figure 1) and well-resolved signals of reference guest **4** (see signals marked with squares in Figure 1). The concentration of CB[7] was 2.0 mM. Affinities were then normalized using silane **3a** as reference.

$$\mathbf{4}\bullet\mathrm{CB[7]} + \mathbf{3} \rightleftharpoons \mathbf{3}\bullet\mathrm{CB[7]} + \mathbf{4}$$

$$K_{rel} = \frac{[\mathbf{3}\bullet\mathrm{CB[7]}][\mathbf{4}]}{[\mathbf{4}\bullet\mathrm{CB[7]}][\mathbf{3}]}$$

Table 1. Binding affinities of guests **3** and Swain-Lupton parameters for each substituent.

Guest	X	F [a,b]	R [a,c]	$fF + rR$ [d]	K_X/K_H [e]	Log (K_X/K_H)
**3a**	H	0.00	0.00	0.00	1.00	0.00
**3b**	CH_3	0.01	-0.18	-0.05	0.88	-0.05
**3c**	$COOCH_3$	0.34	0.11	0.26	1.67	0.22
**3d**	CF_3	0.38	0.16	0.31	1.77	0.25
**3e**	CN	0.51	0.15	0.39	2.22	0.35
**3f**	NO_2	0.65	0.13	0.48	2.46	0.39
**3g**	Br	0.45	-0.22	0.23	1.60	0.20
**3h**	F	0.45	-0.39	0.17	1.39	0.14
**3i**	OCH_3	0.29	-0.56	0.01	0.99	0.00
**3j**	SO_2CF_3	0.74	0.22	0.57	3.13	0.50
**3k**	$B(OH)_2$	-0.03	0.15	0.03	1.05	0.02

[a] From reference [6]. [b] Swain-Lupton field/inductive parameters. [c] Swain-Lupton resonance parameters. [d] Linear combination of Swain-Lupton parameters optimized for guests **3**; $f = 0.667$, $r = 0.333$. [e] Binding affinities relative to reference silane **3a**. The error is typically within 3%.

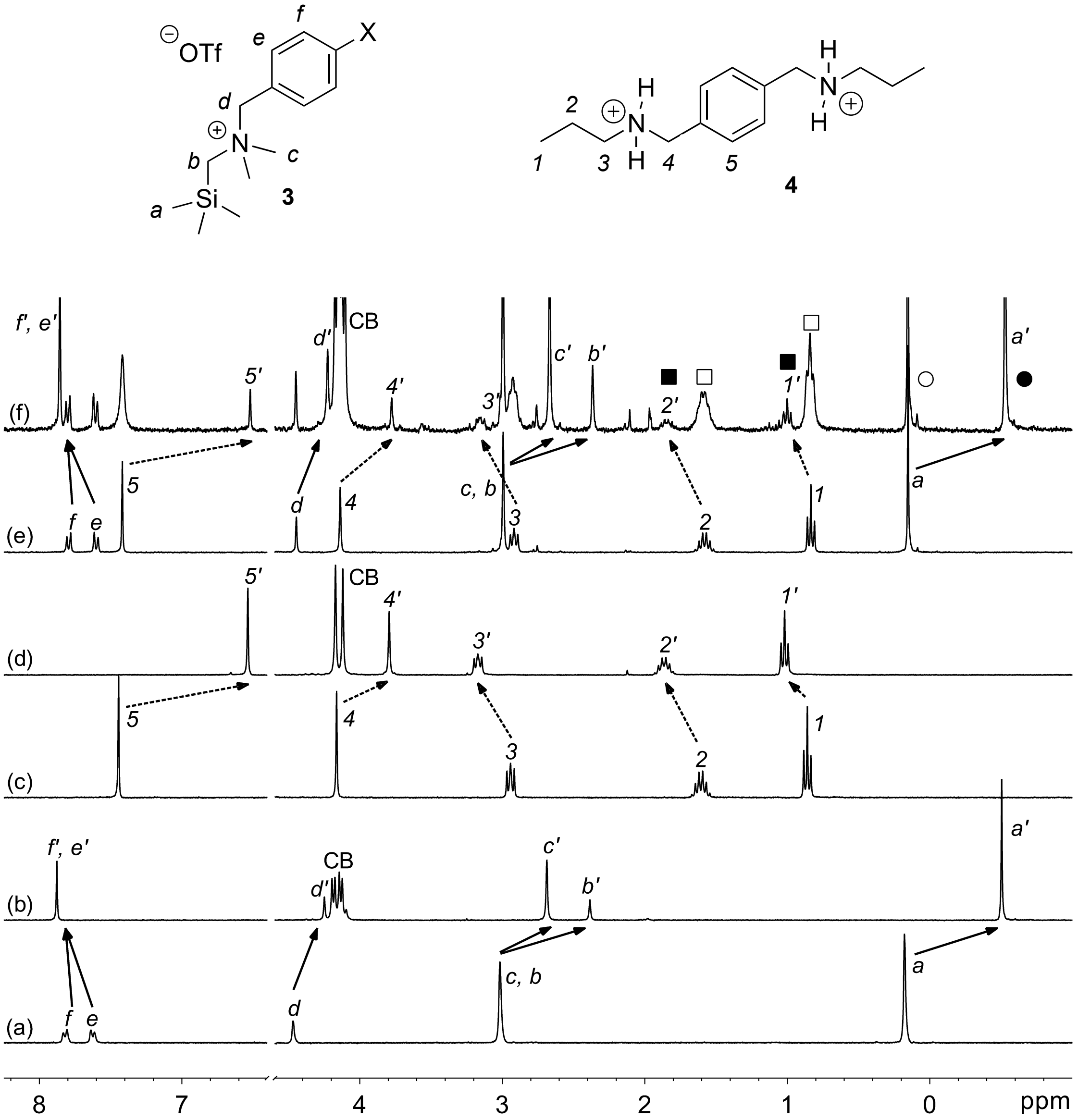

Figure 1. ^{1}H NMR spectra of (a) silane **3e** (X = CN), (b) complex **3e**·CB[7], (c) reference xylylene diammonium **4**, (d) complex **4**·CB[7], (e) a mixture of silane **3e** and reference **4**, and (f) the same mixture after addition of 1.0 equiv. CB[7]. All spectra are recorded in deuterium oxide. Empty and filled circles indicate the signals of free and CB[7]-bound silane **3e** (or silanes **3** in general) used for quantification, and empty and filled squares the signals of free and CB[7]-bound reference **4**. See structures for numbering.

5. Isothermal Titration Calorimetry experiments

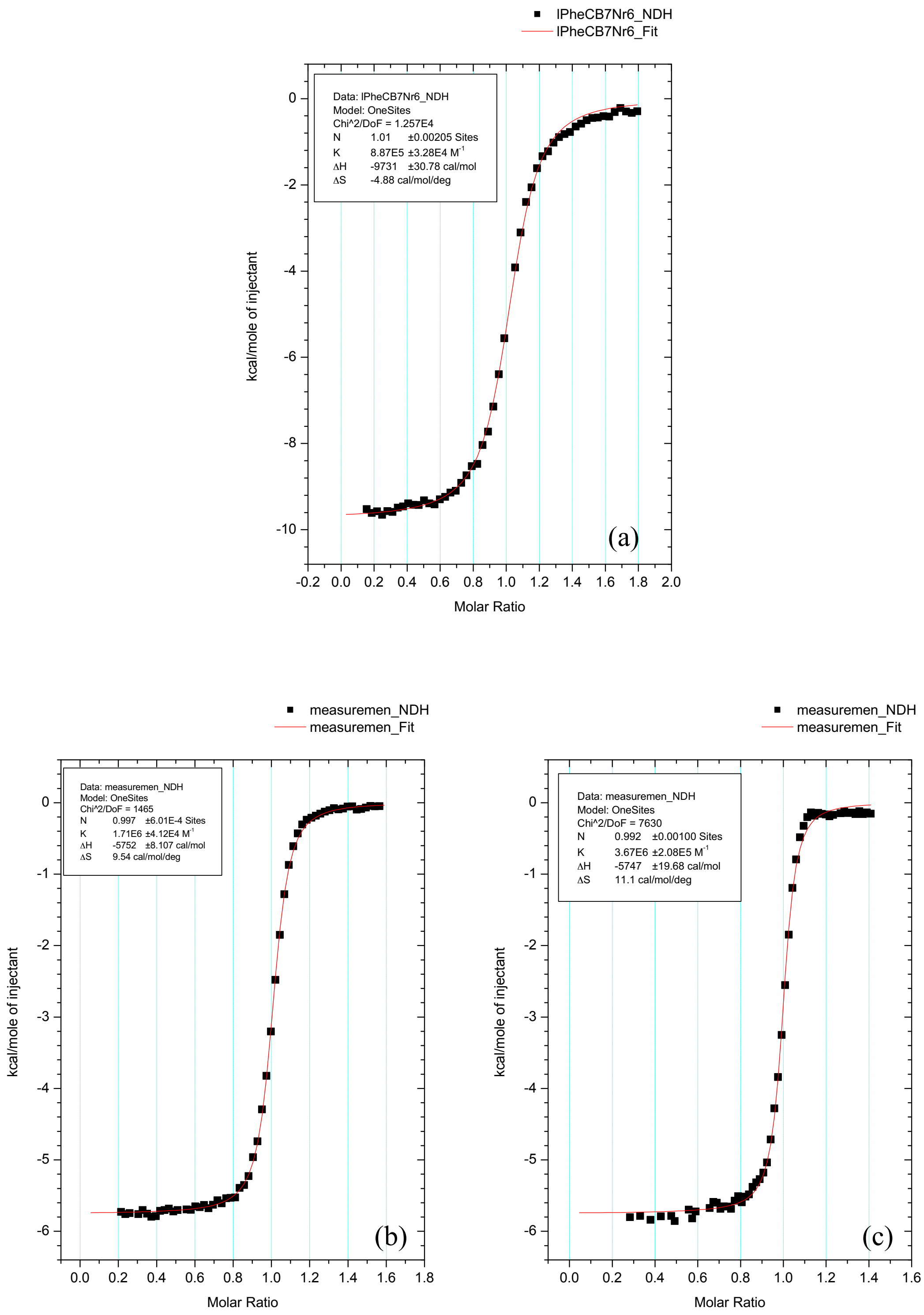

Figure 2. ITC experiment on complexation of (a) L-Phenylalanine (L-Phe; 4.0 mM), (b) silane **3a** with CB[7] in a solution of L-Phe (4.0 mM) and (c) silane **3f** with CB[7] in a solution of L-Phe (4.0 mM).

The thermodynamic parameters for the inclusion of guest **3a** and **3f** into CB[7] were determined by isothermal titration calorimetry (ITC). An aqueous solution of CB[7] (0.40 mM) and L-Phe (4.0 mM) was placed in the sample cell, to which a solution of silane **3a** or **3f** (2.7 – 6.7 mM) was added stepwise in a series of 60 – 70 injections (3 min interval, 0.2 – 1.0 μL each). Enthalpies were corrected by subtracting the heat of dilution, which was measured by repeating the experiment with pure water in the sample cell. The data were analyzed and fitted with the Origin software.

All titrations were carried out in triplicate. Deviations between replicates and errors from fitting are both used to calculate standard deviations. In all titrations, the stoichiometry of binding *N* was found to be within 1% of 1.0. This deviation has strictly no impact on the thermodynamic parameters.

6. ^{1}H and ^{13}C NMR spectra of silanes 3a – 3k

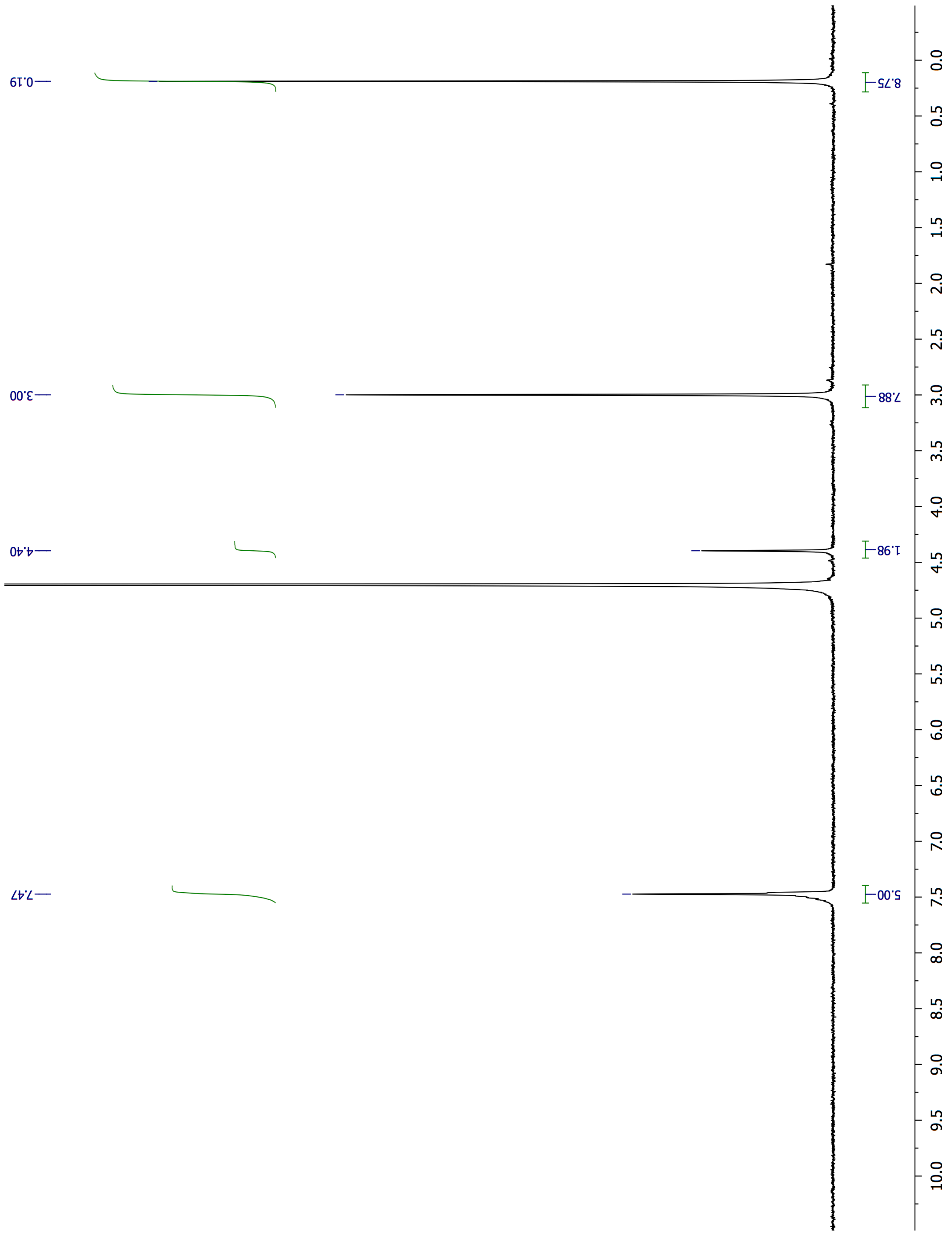

Figure 3. ^{1}H NMR spectrum of TMS derivative **3a** in D_2O.

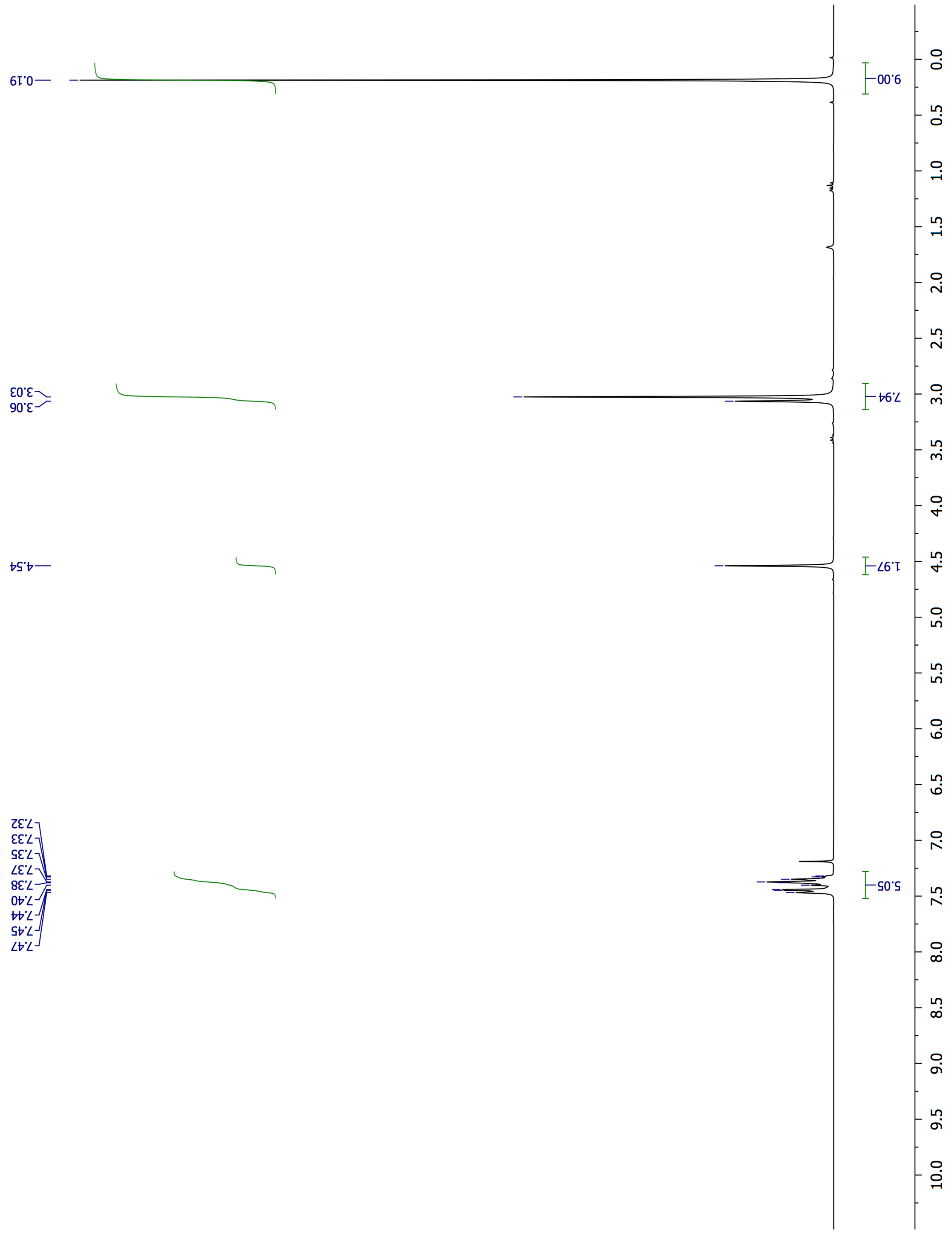

Figure 4. ^{1}H NMR spectrum of TMS derivative **3a** in $CDCl_3$.

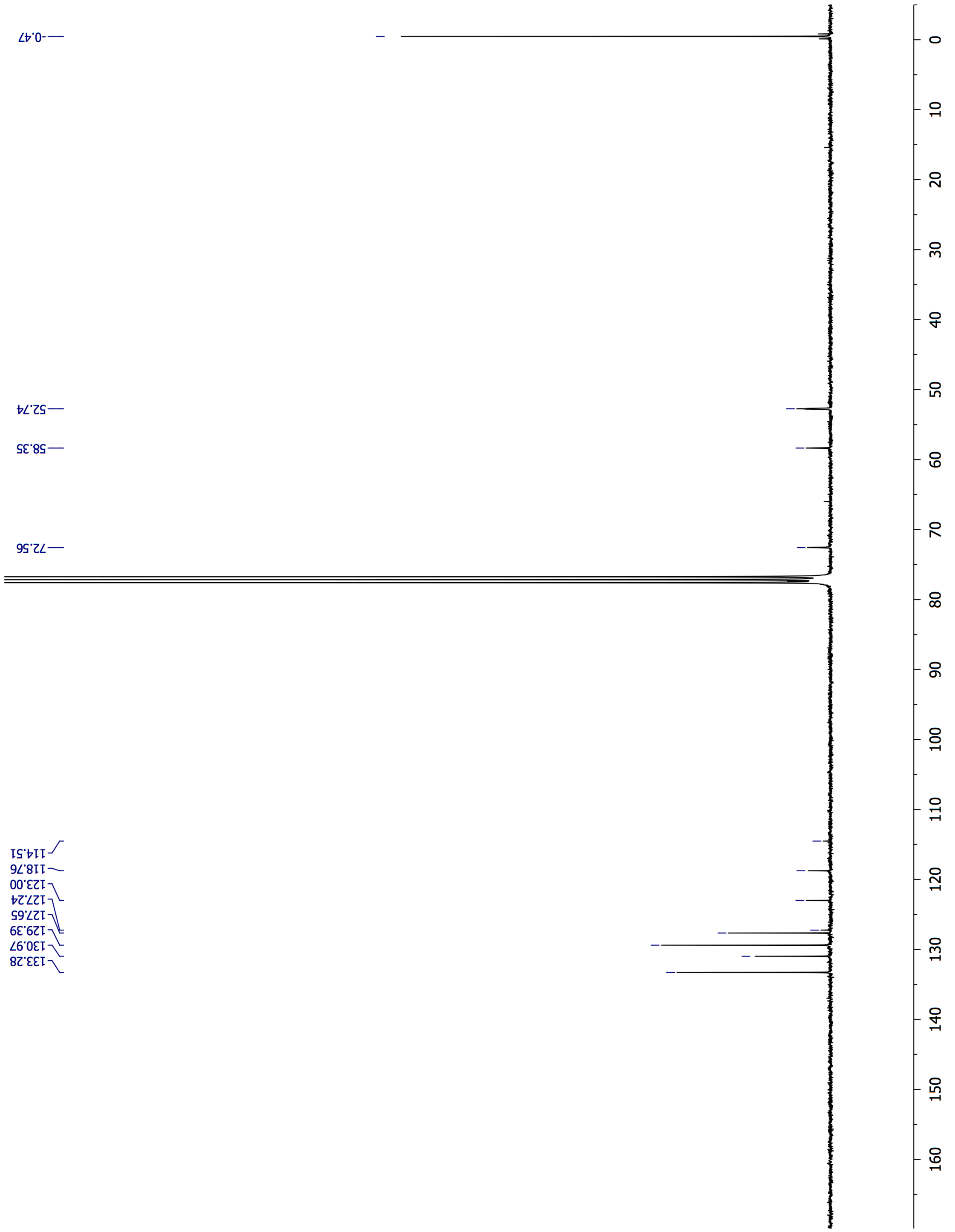

Figure 5. ^{13}C NMR spectrum of TMS derivative **3a** in $CDCl_3$.

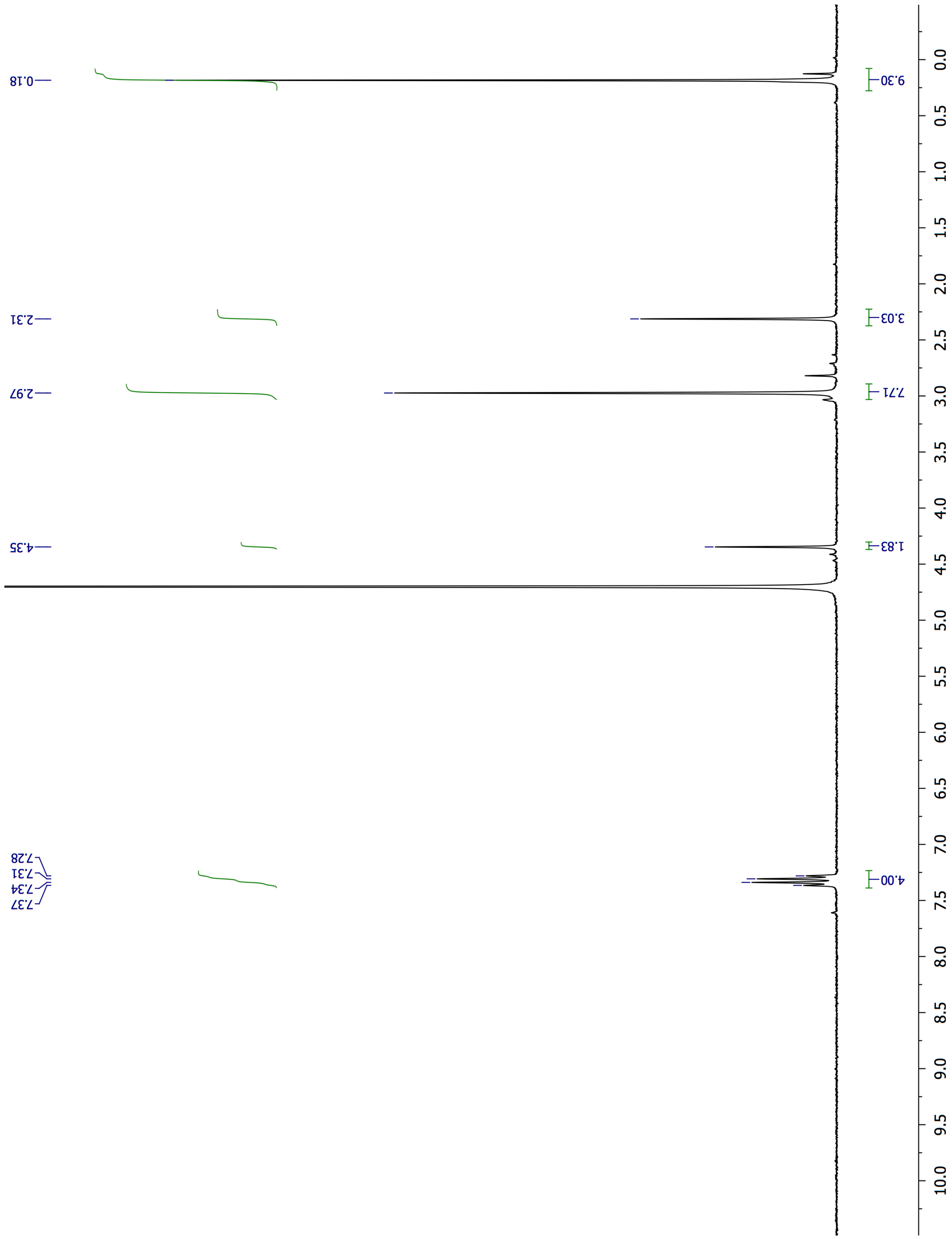

Figure 6. ^{1}H NMR spectrum of TMS derivative **3b** in D_2O.

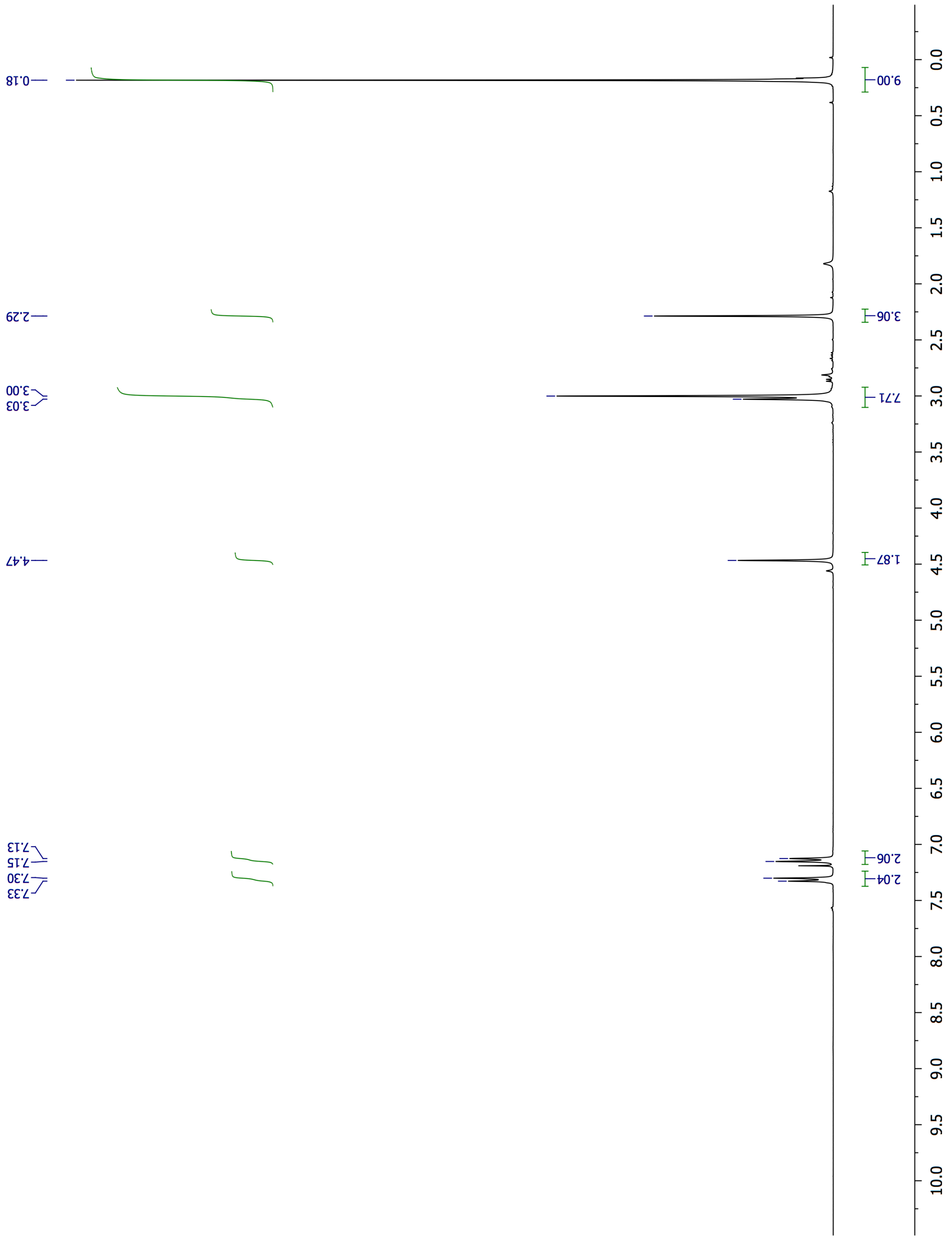

Figure 7. ^{1}H NMR spectrum of TMS derivative **3b** in $CDCl_3$.

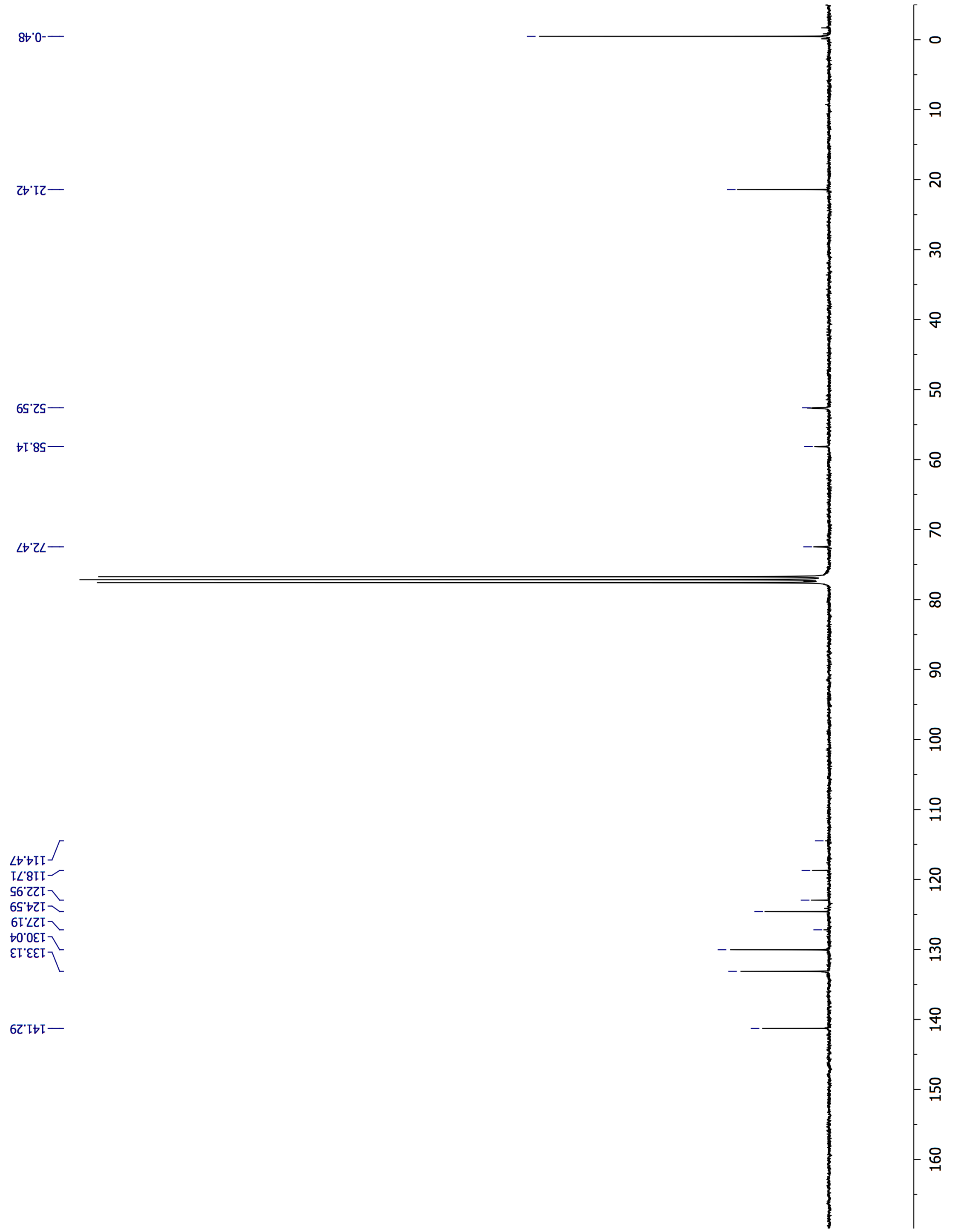

Figure 8. ^{13}C NMR spectrum of TMS derivative **3b** in $CDCl_3$.

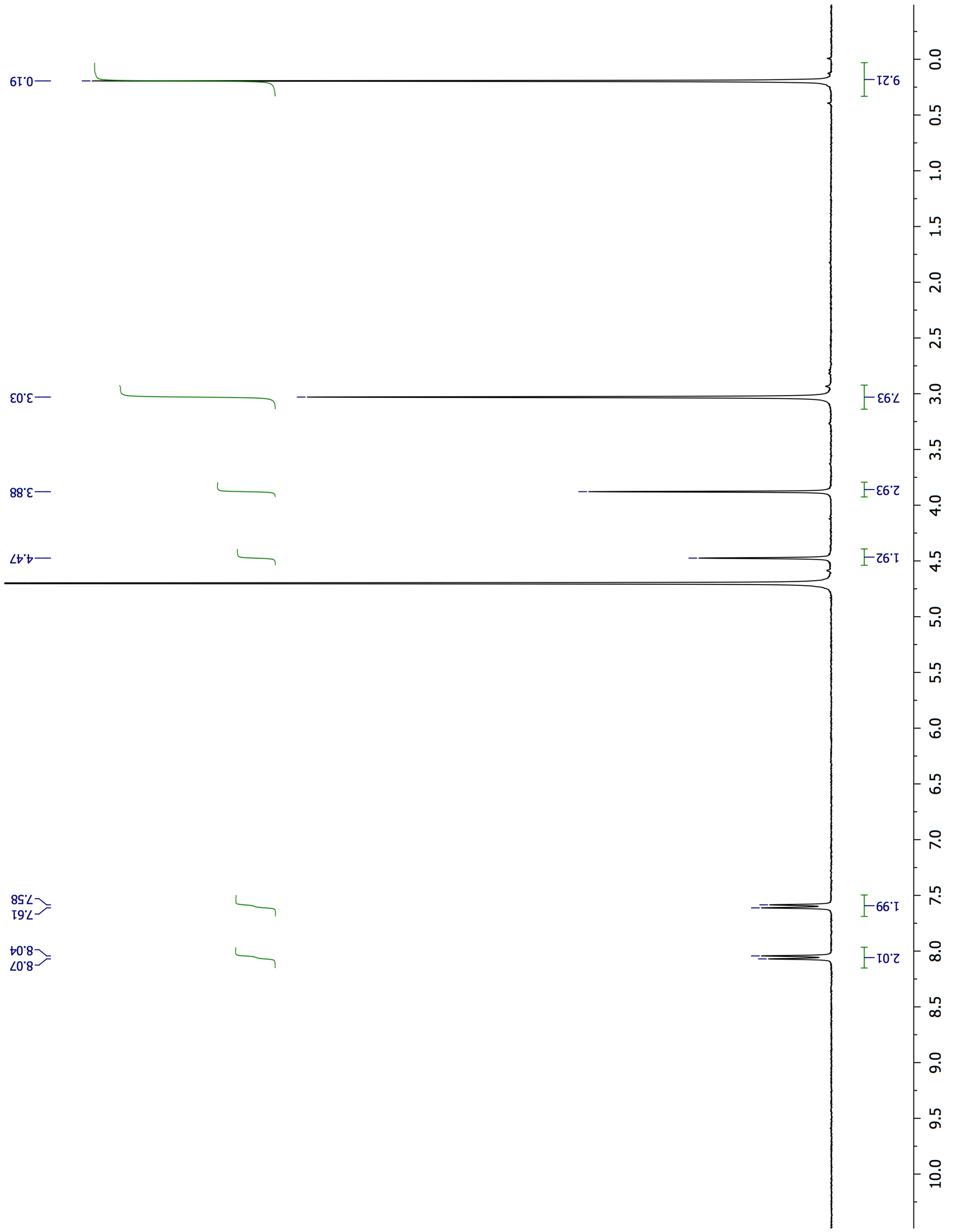

Figure 9. ^{1}H NMR spectrum of TMS derivative **3c** in D_2O.

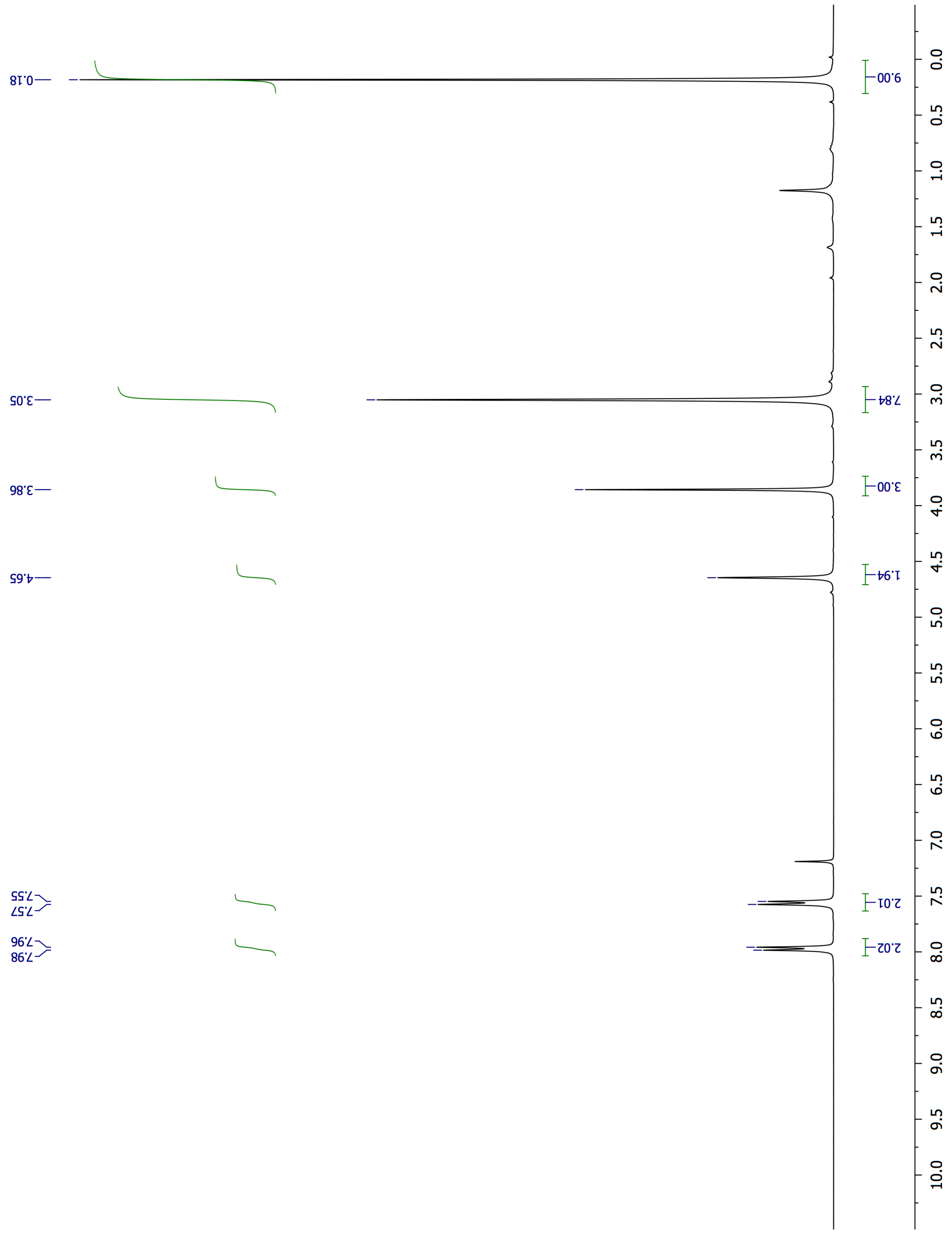

Figure 10. ^{1}H NMR spectrum of TMS derivative **3c** in $CDCl_3$.

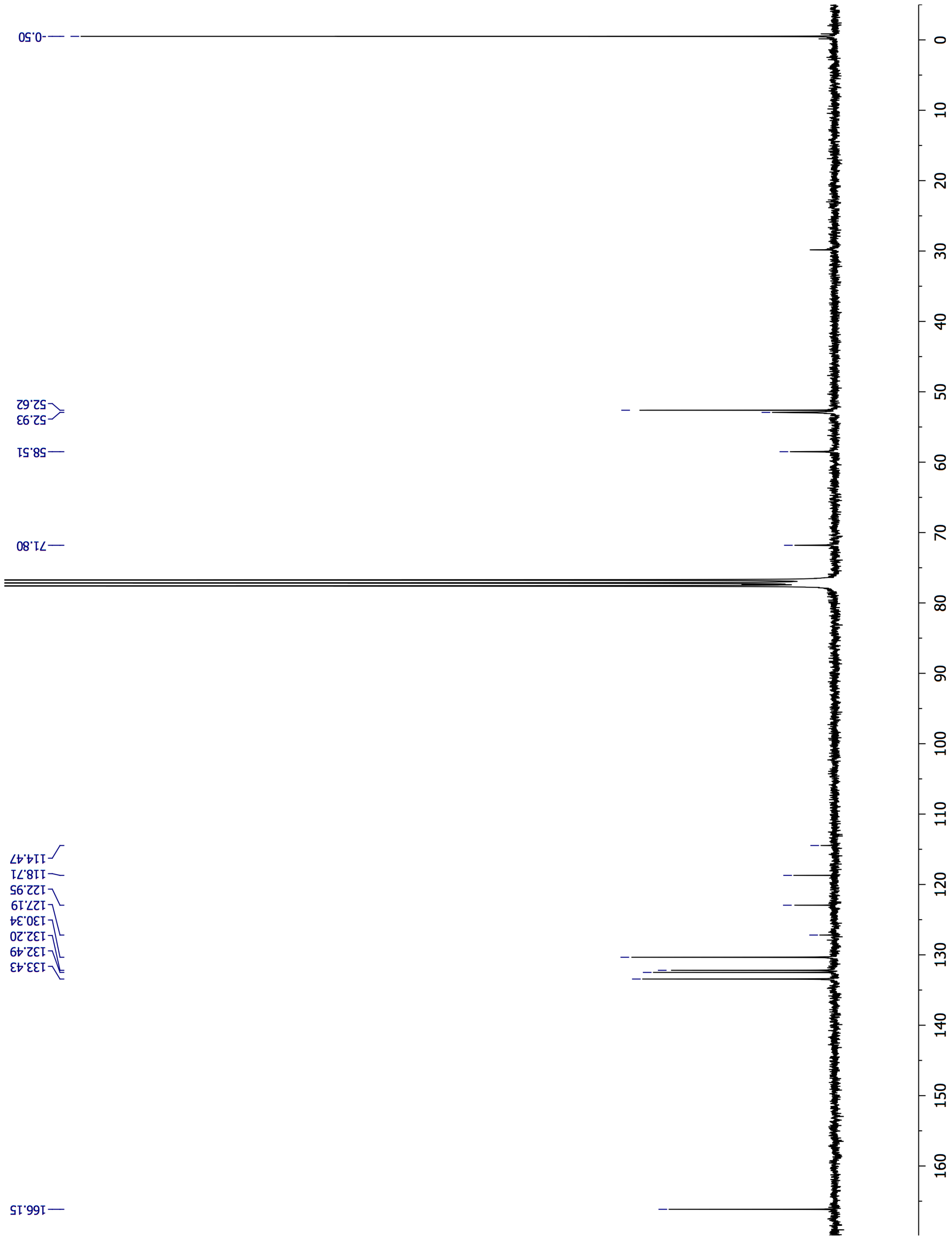

Figure 11. ^{13}C NMR spectrum of TMS derivative **3c** in $CDCl_3$.

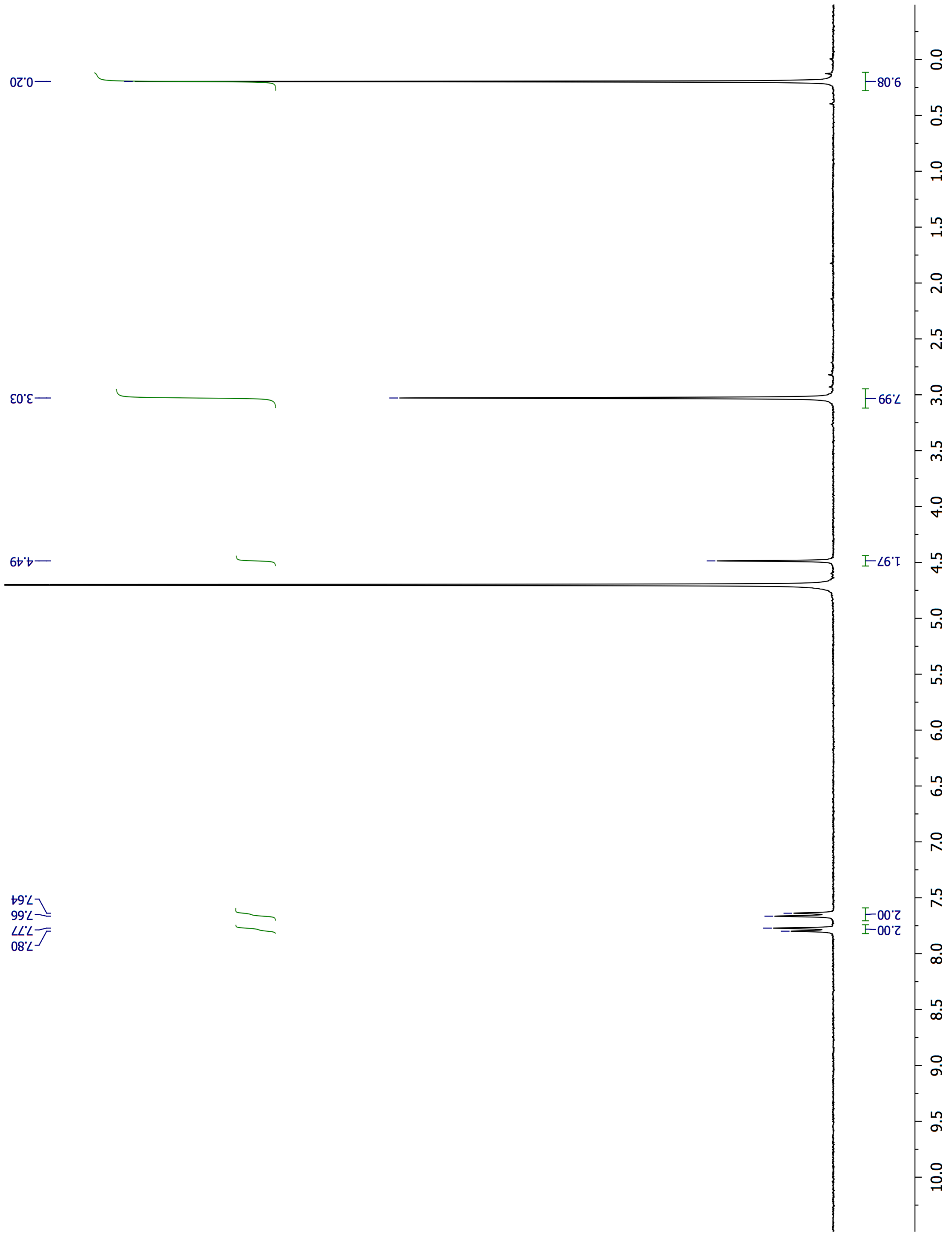

Figure 12. ^{1}H NMR spectrum of TMS derivative **3d** in D_2O.

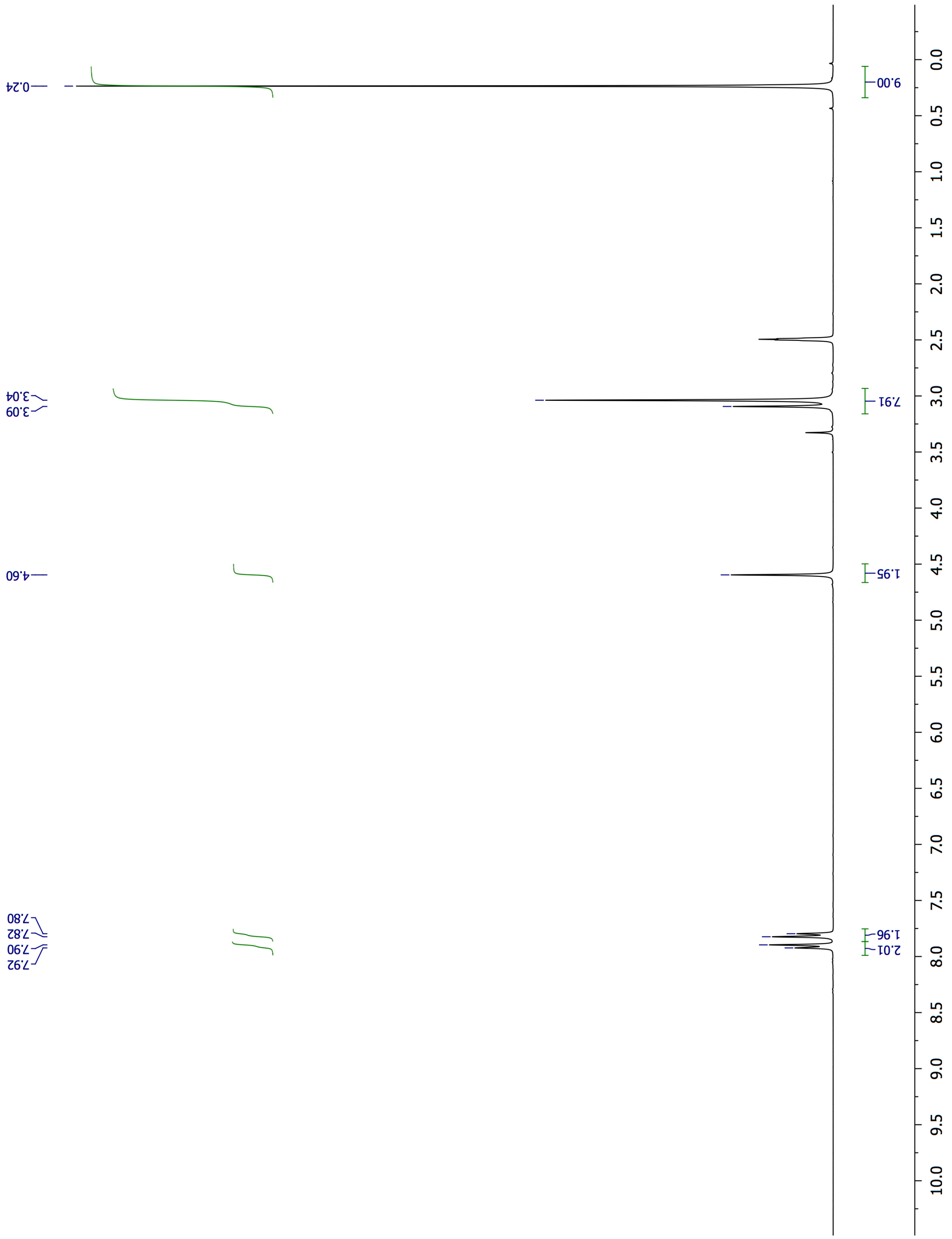

Figure 13. ^{1}H NMR spectrum of TMS derivative **3d** in DMSO-d^{6}.

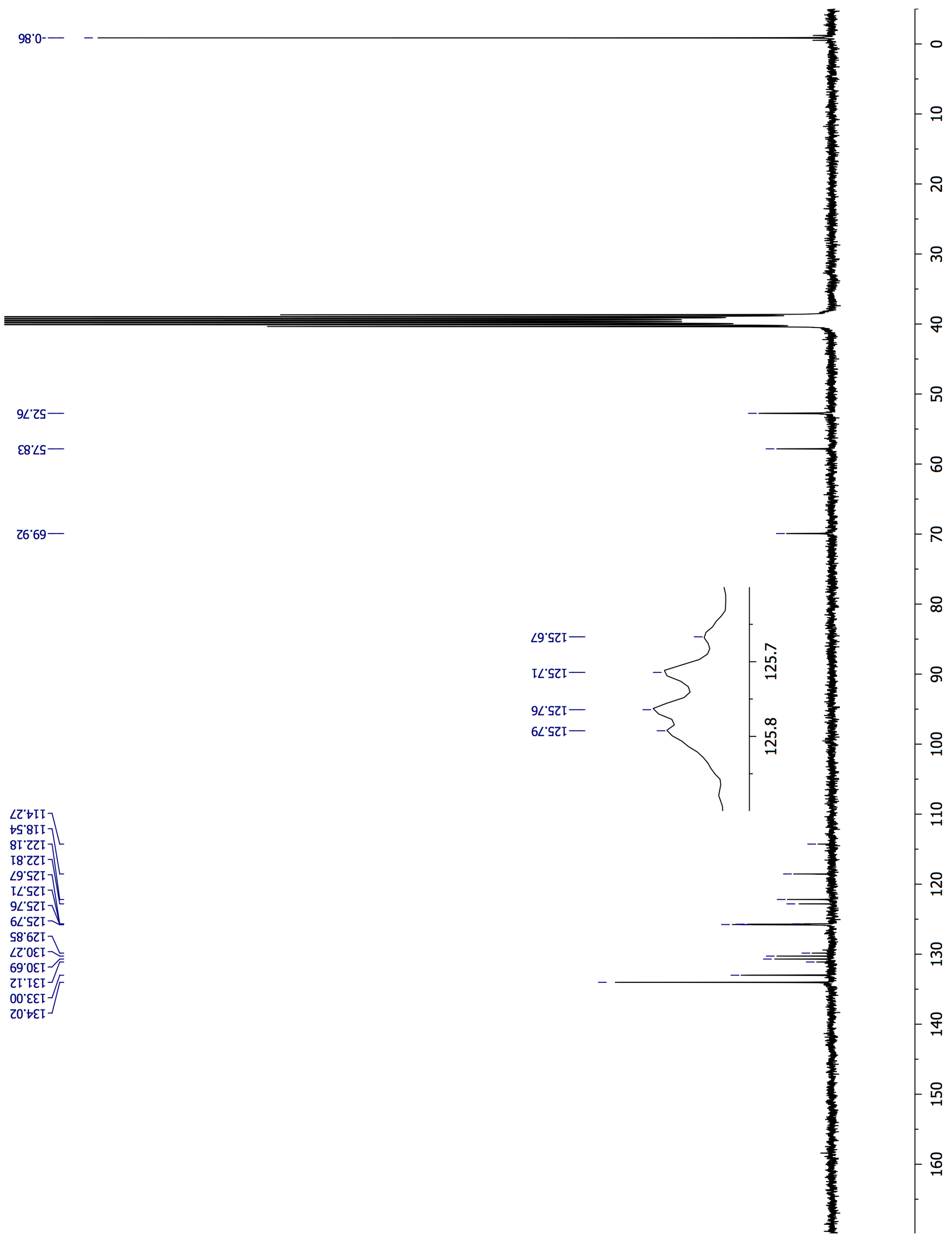

Figure 14. ^{13}C NMR spectrum of TMS derivative **3d** in DMSO-d^6.

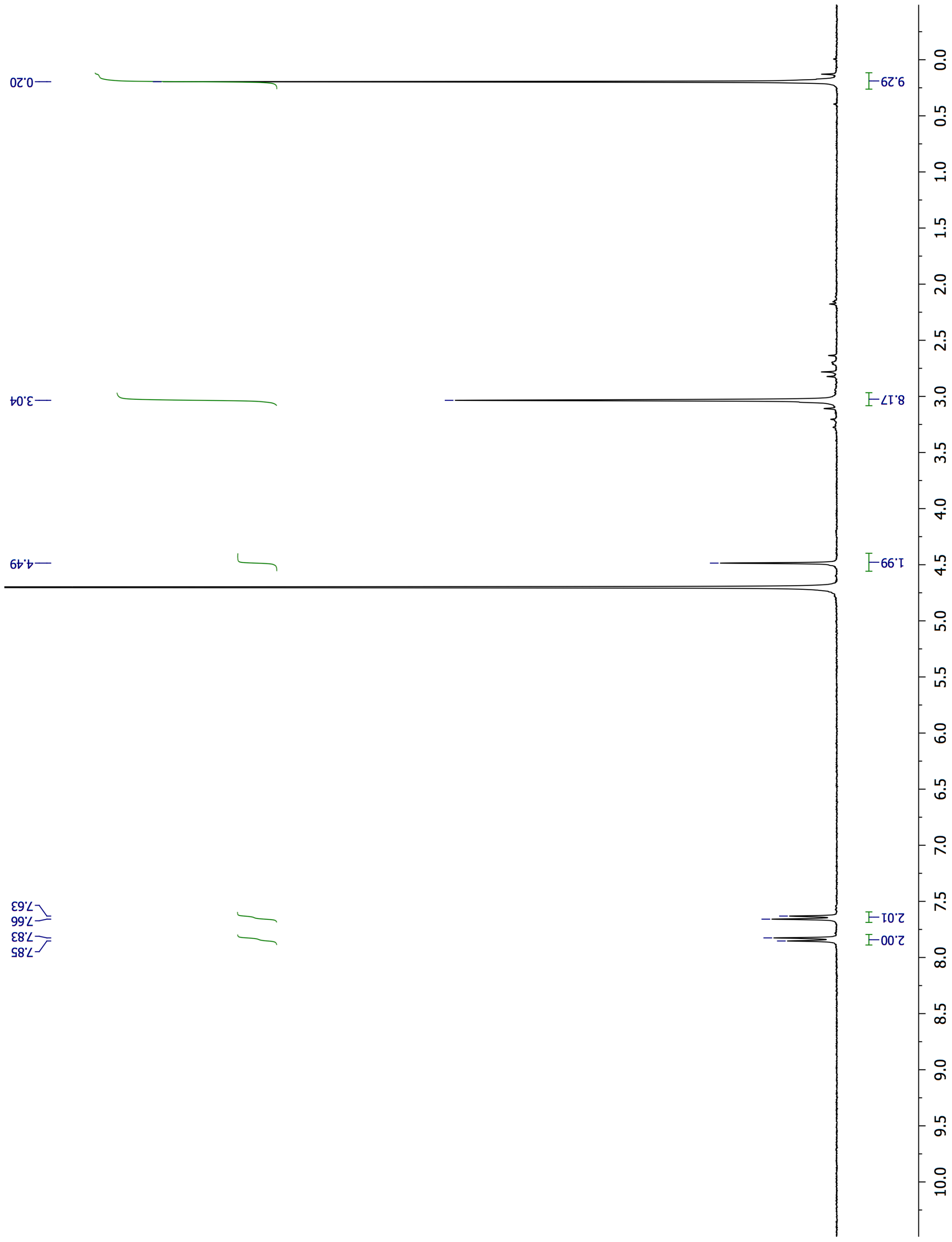

Figure 15. ^{1}H NMR spectrum of TMS derivative **3e** in D_2O.

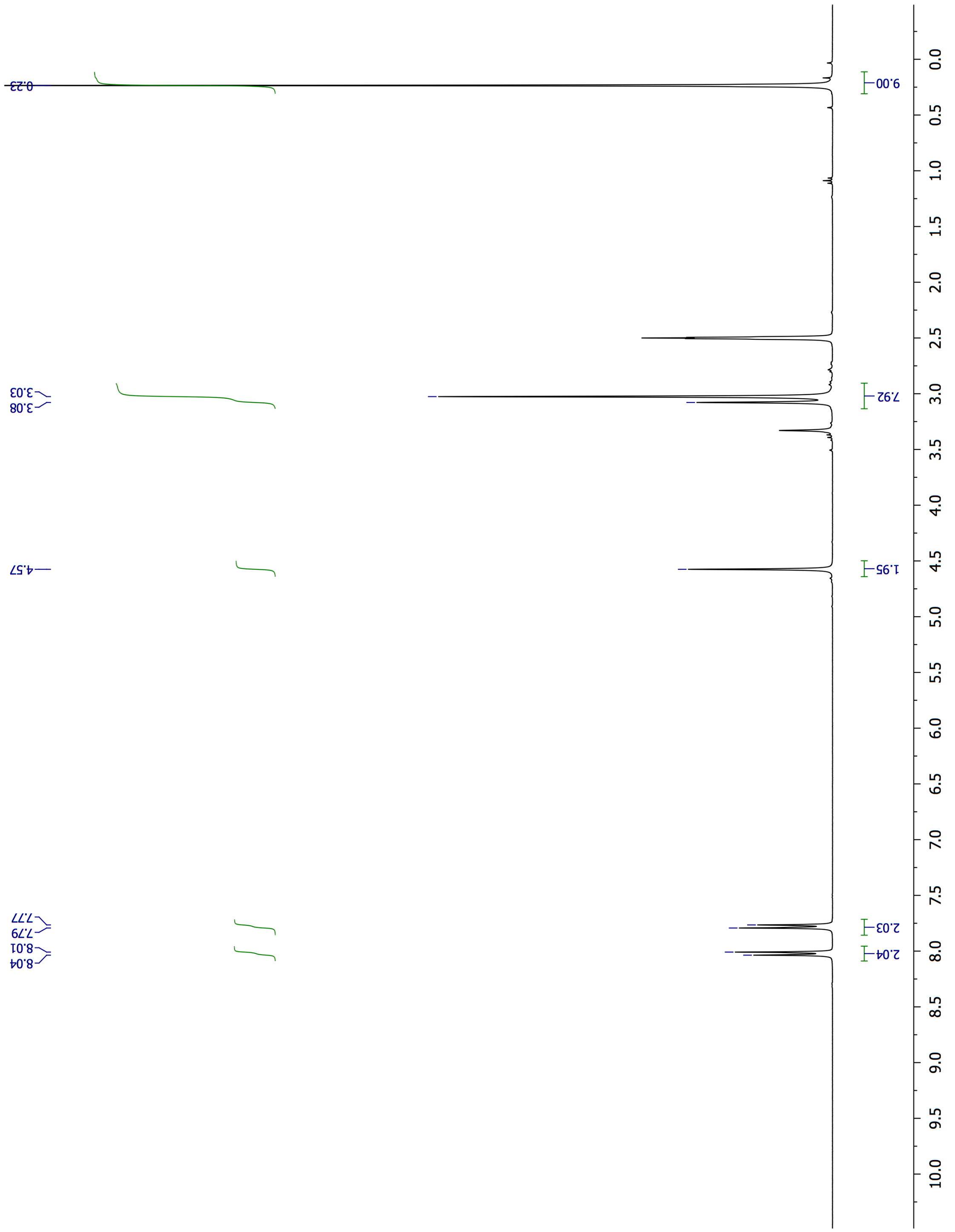

Figure 16. ^{1}H NMR spectrum of TMS derivative **3e** in DMSO-d^{6}.

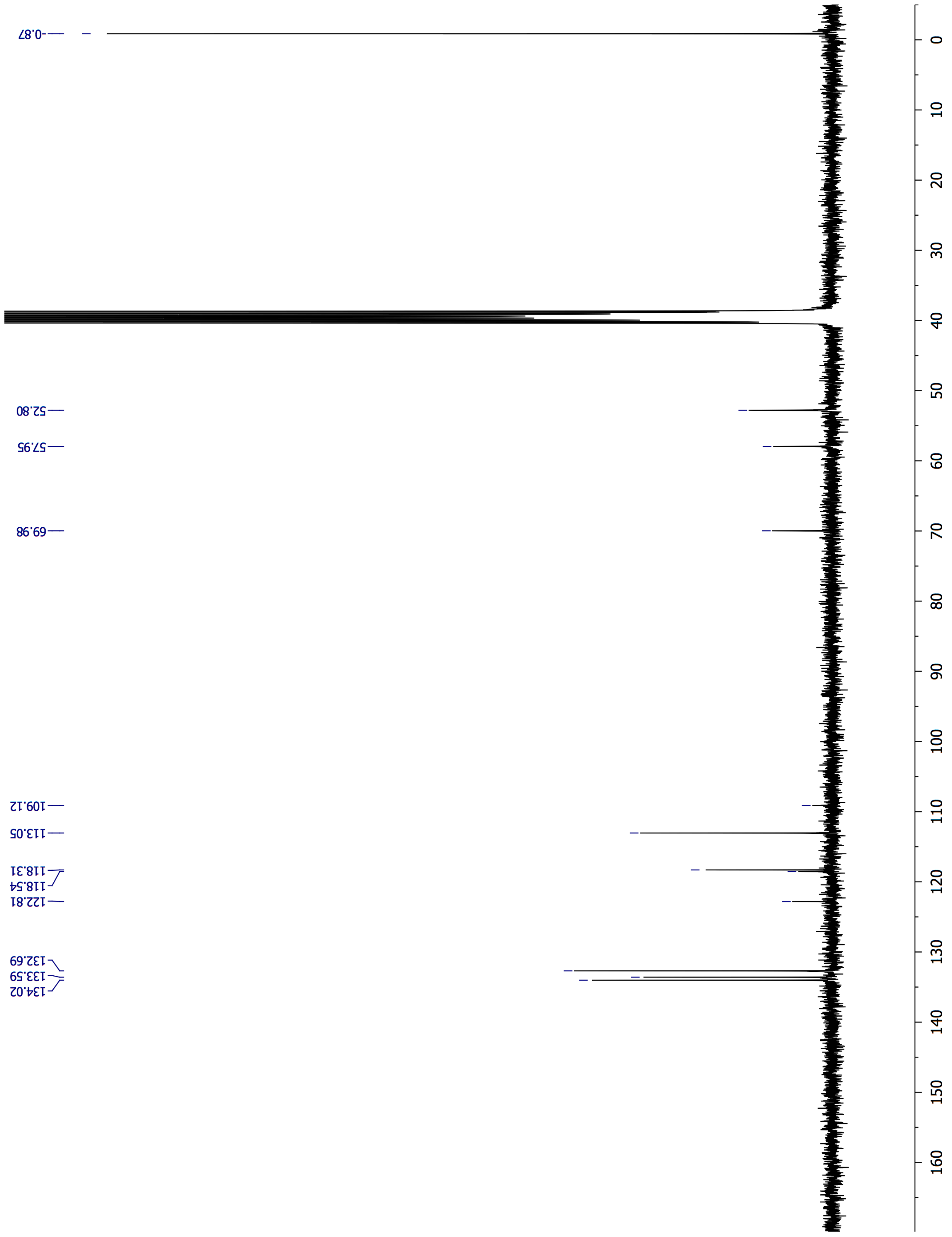

Figure 17. ^{13}C NMR spectrum of TMS derivative **3e** in DMSO-d^{6}.

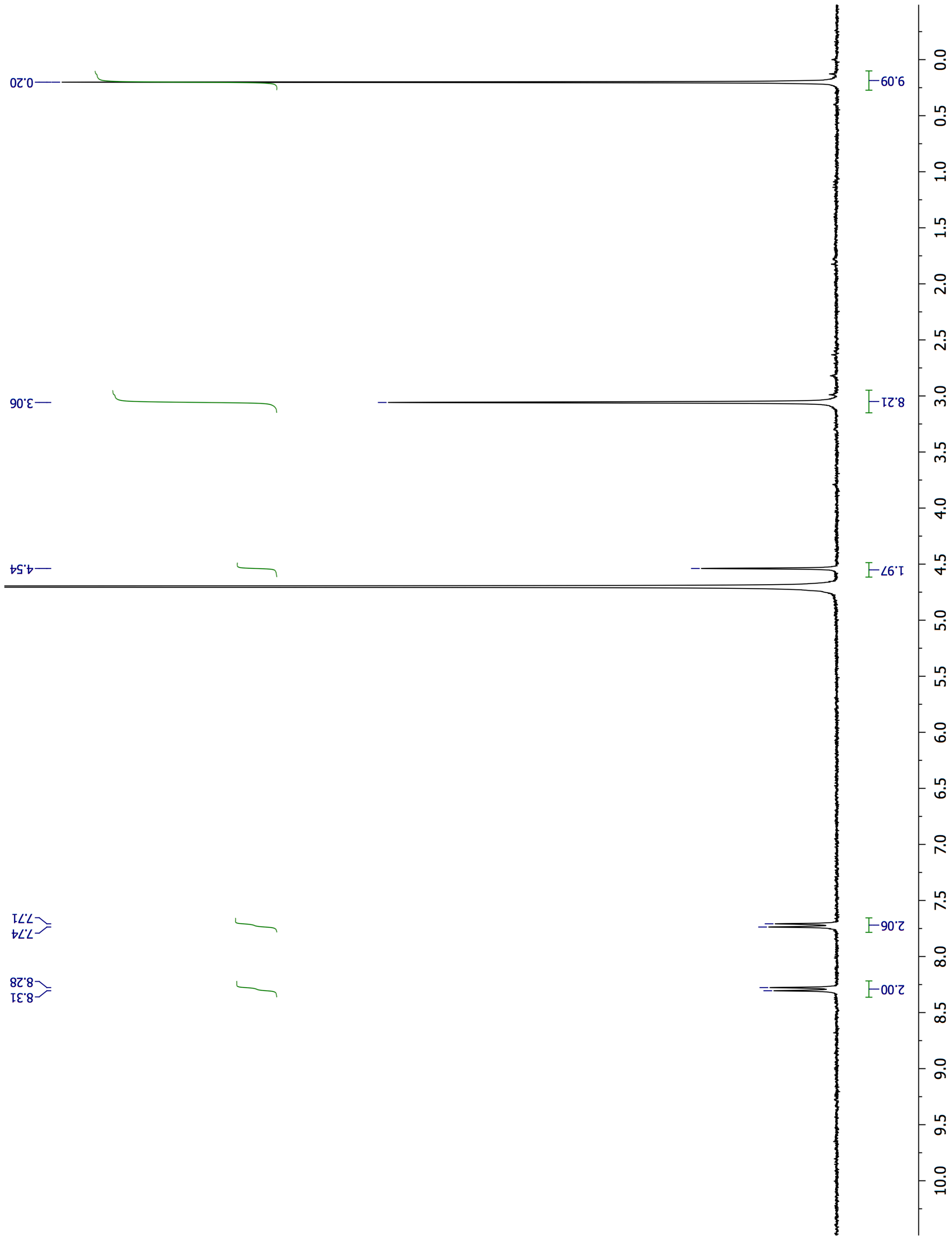

Figure 18. ^{1}H NMR spectrum of TMS derivative **3f** in D_2O.

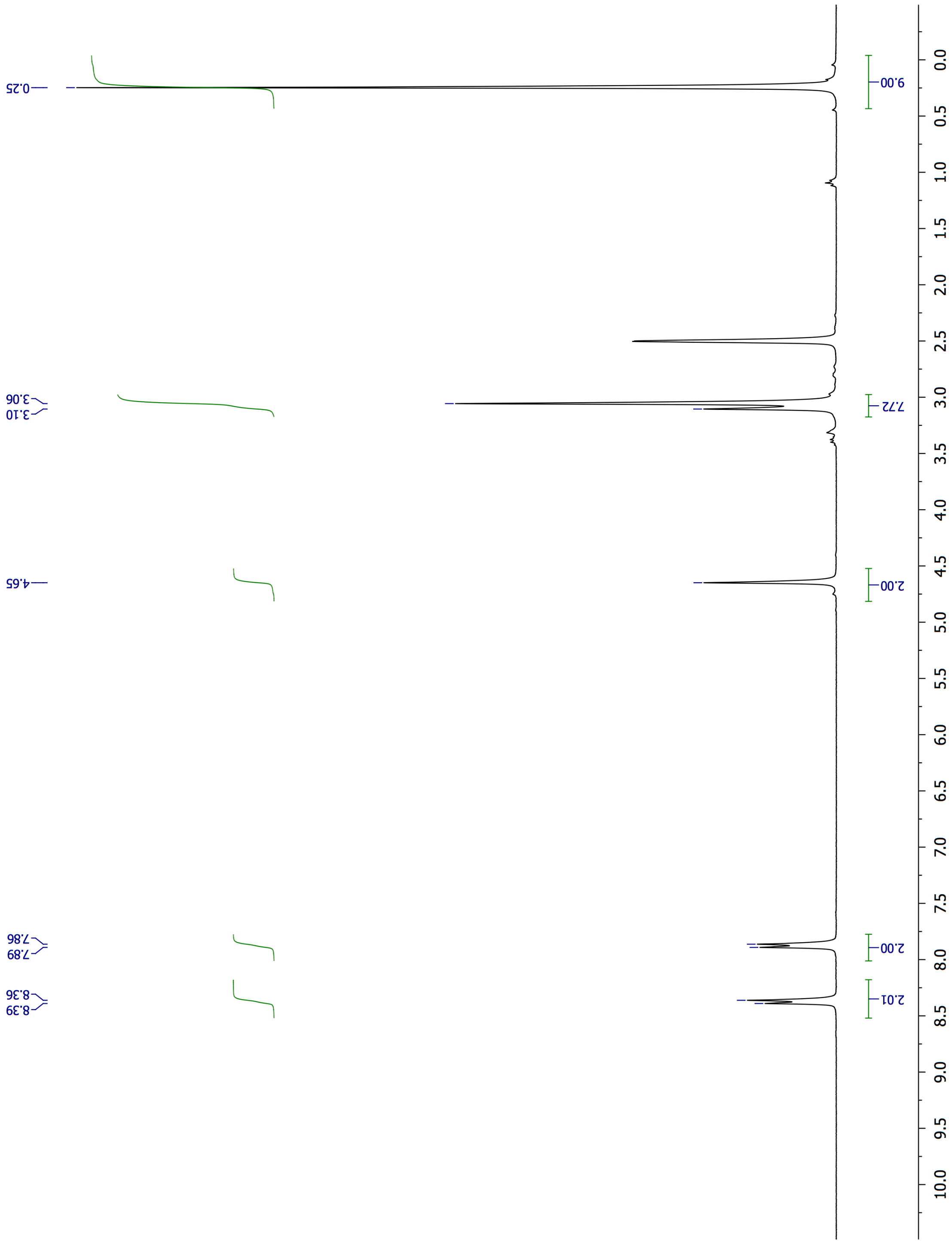

Figure 19. ^{1}H NMR spectrum of TMS derivative **3f** in DMSO-d^{6}.

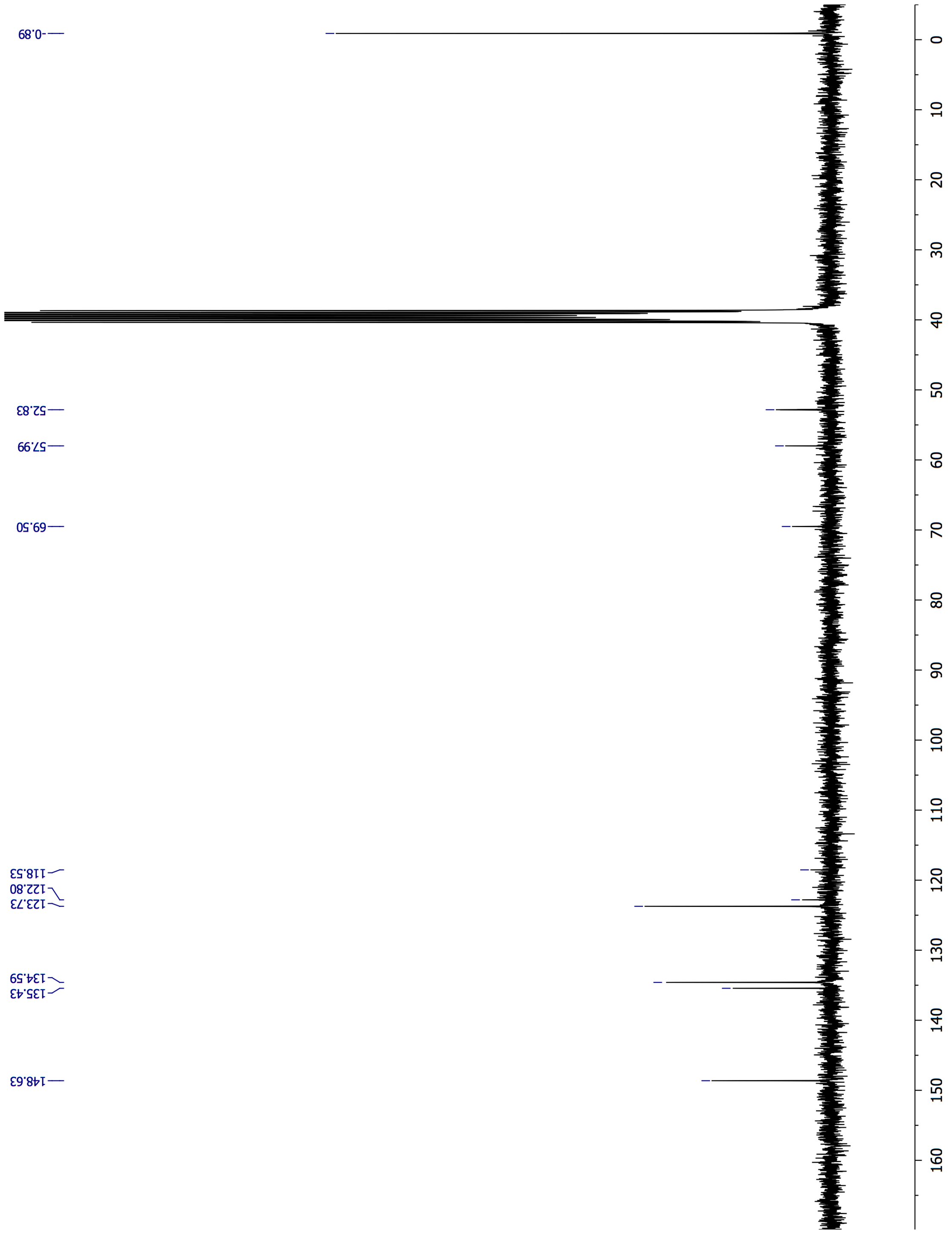

Figure 20. ^{13}C NMR spectrum of TMS derivative **3f** in DMSO-d^6.

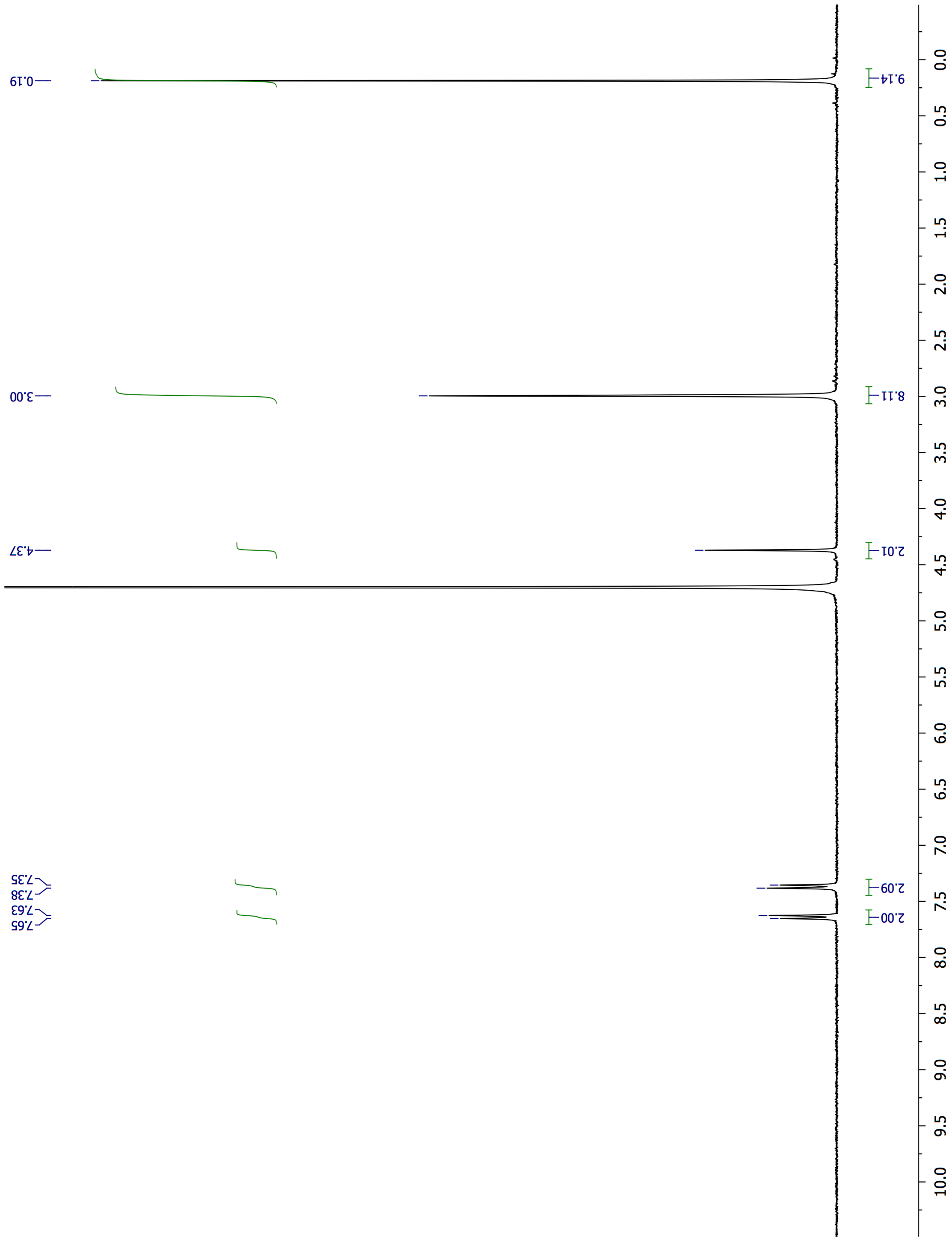

Figure 21. ^{1}H NMR spectrum of TMS derivative **3g** in D_2O.

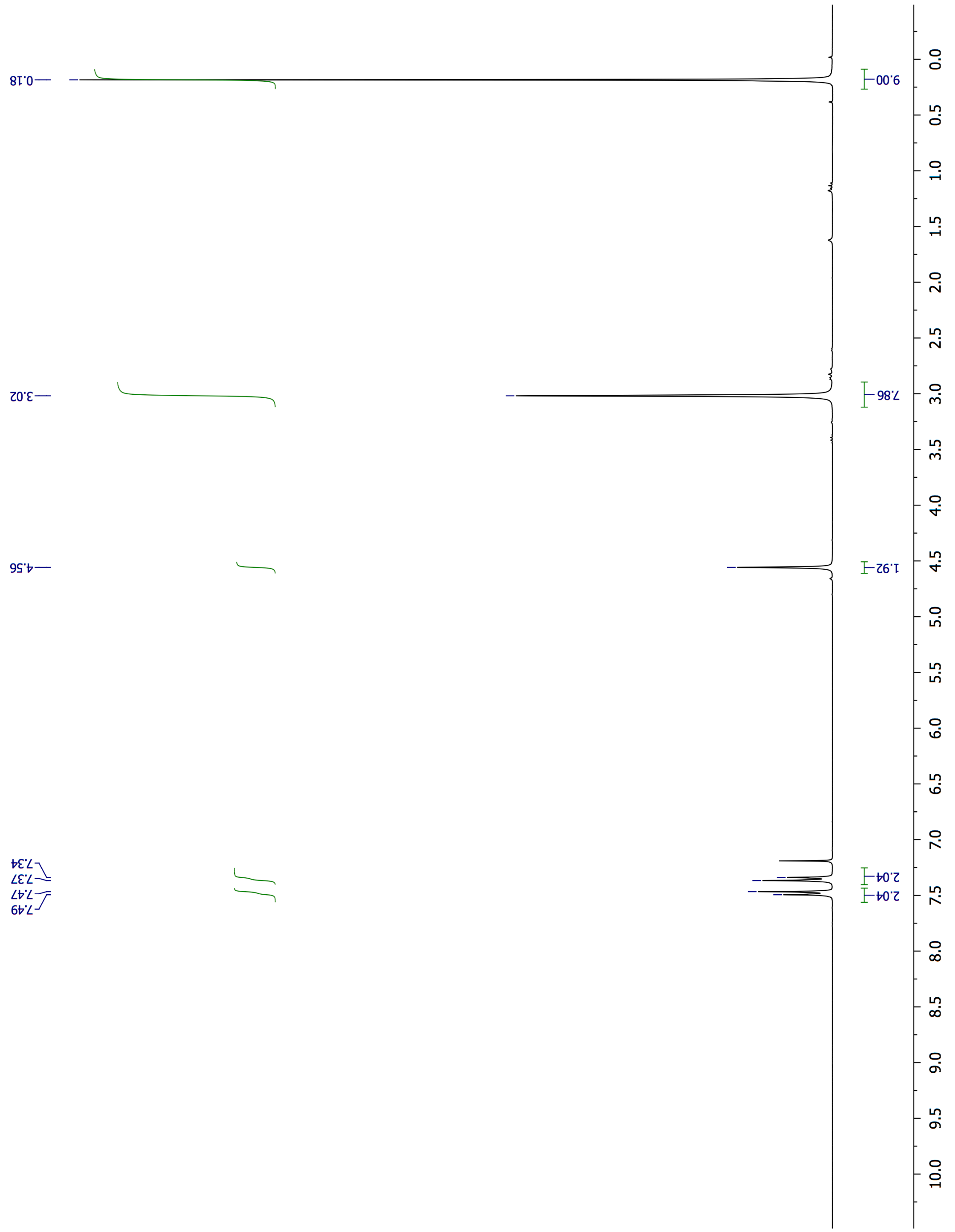

Figure 22. ^{1}H NMR spectrum of TMS derivative **3g** in $CDCl_3$.

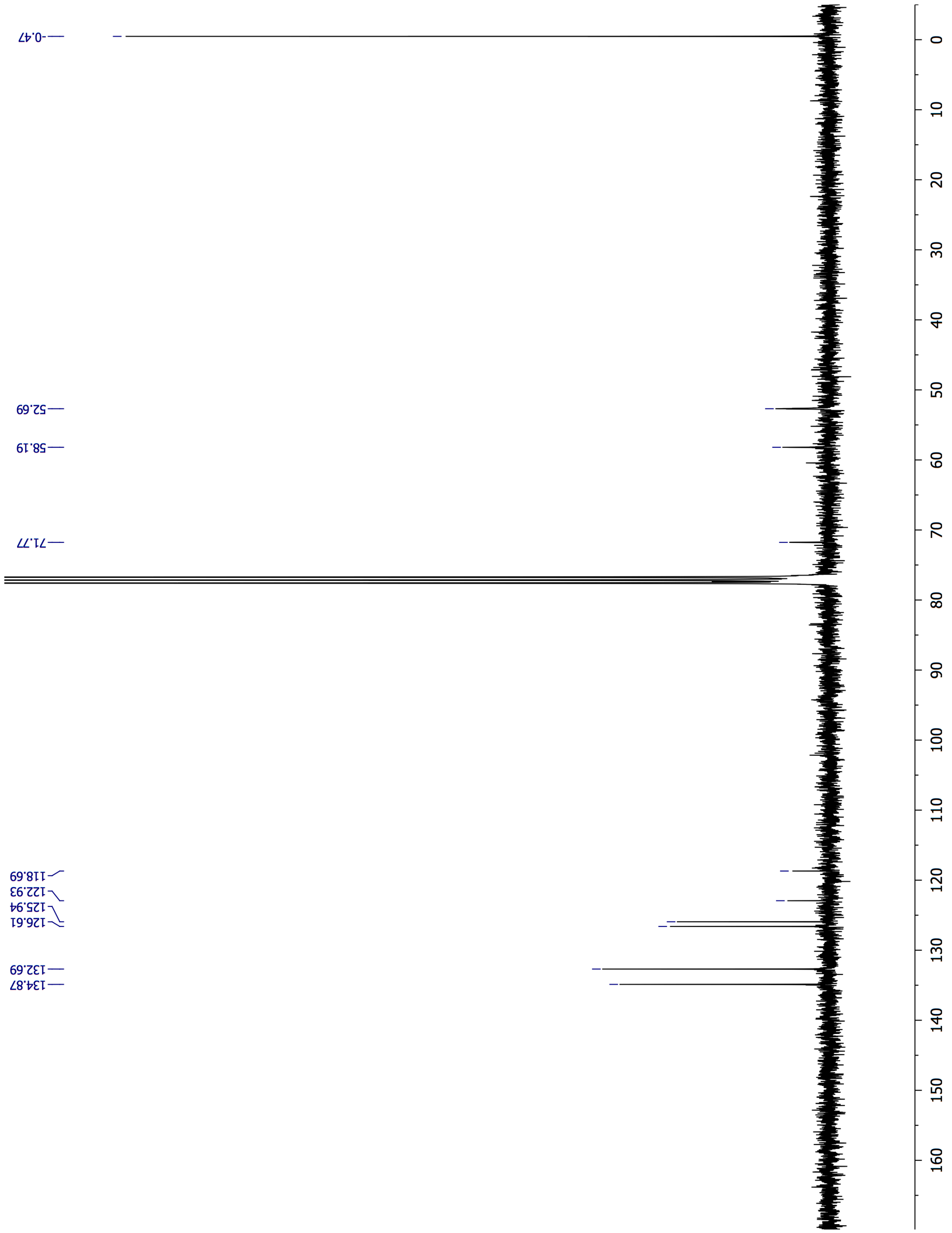

Figure 23. ^{13}C NMR spectrum of TMS derivative **3g** in $CDCl_3$.

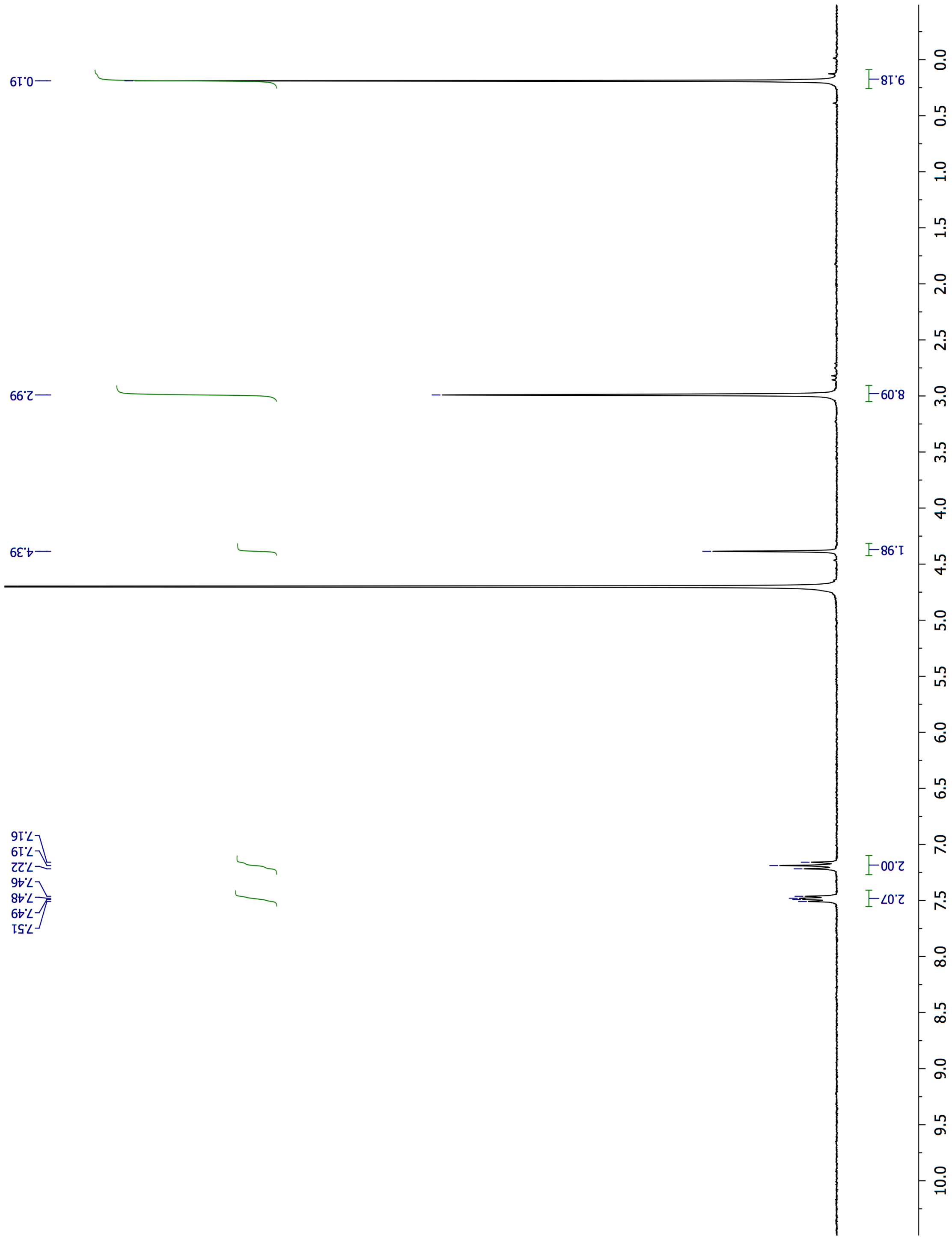

Figure 24. ^{1}H NMR spectrum of TMS derivative **3h** in D_2O.

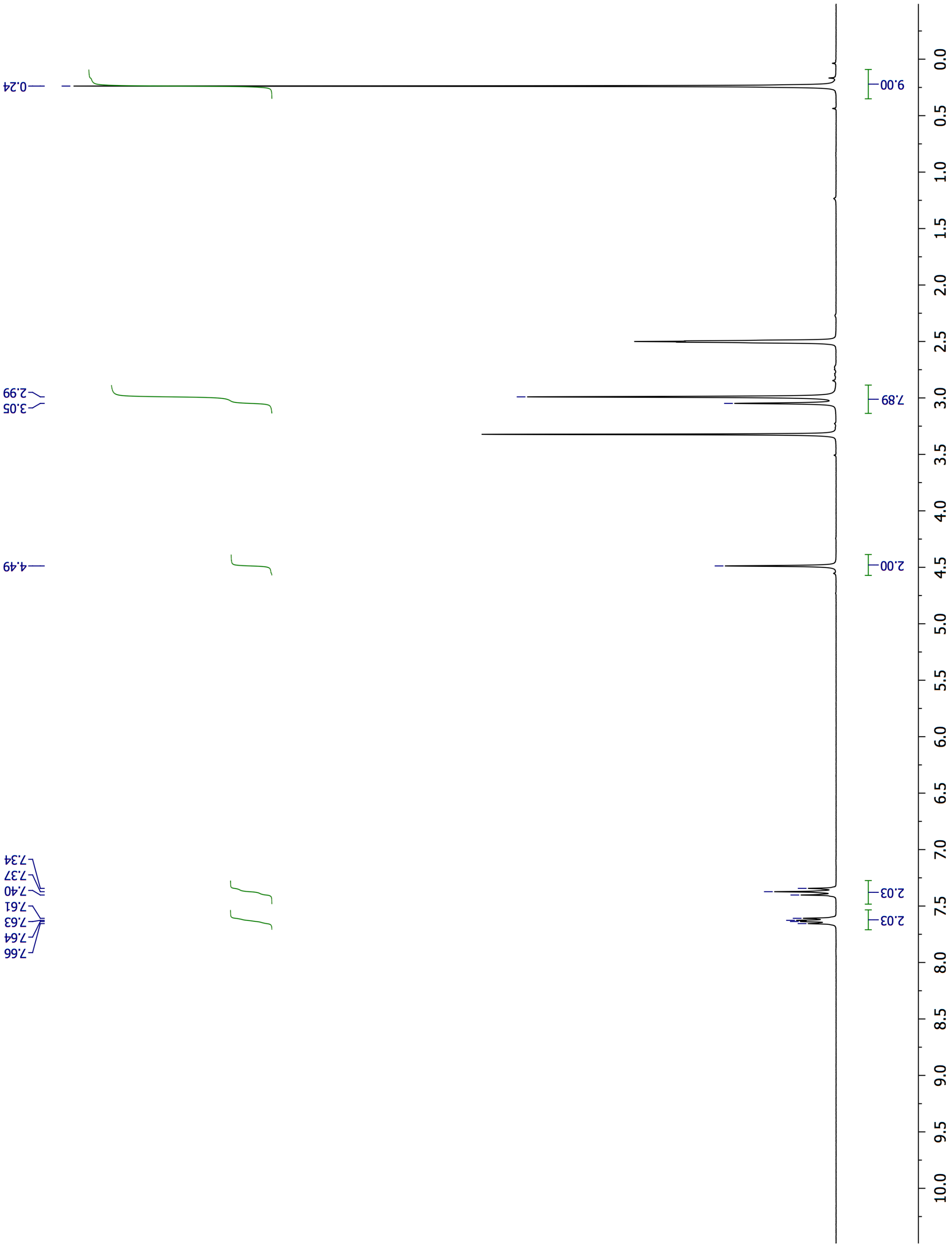

Figure 25. ^{1}H NMR spectrum of TMS derivative **3h** in DMSO-d^6.

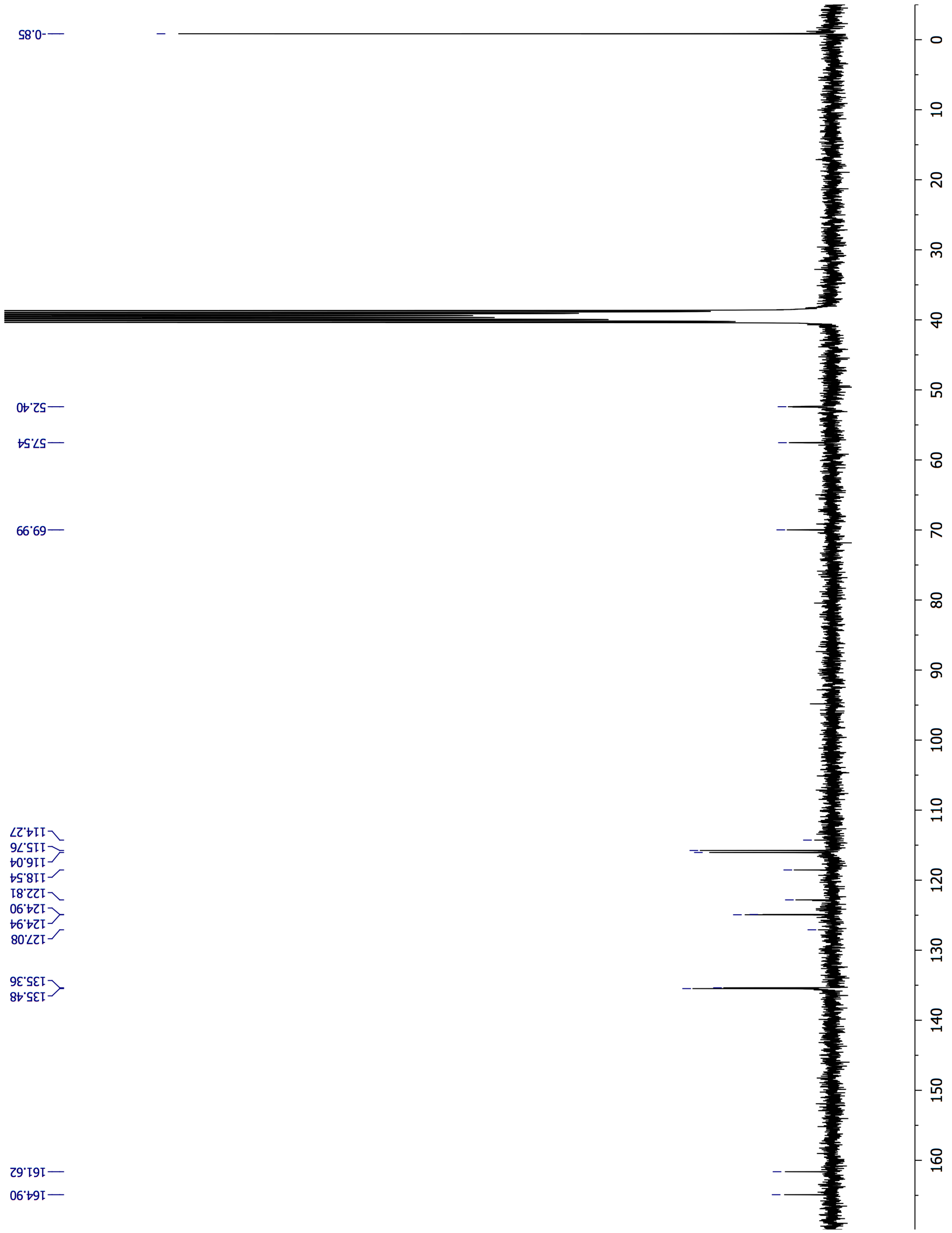

Figure 26. ^{13}C NMR spectrum of TMS derivative **3h** in DMSO-d^6.

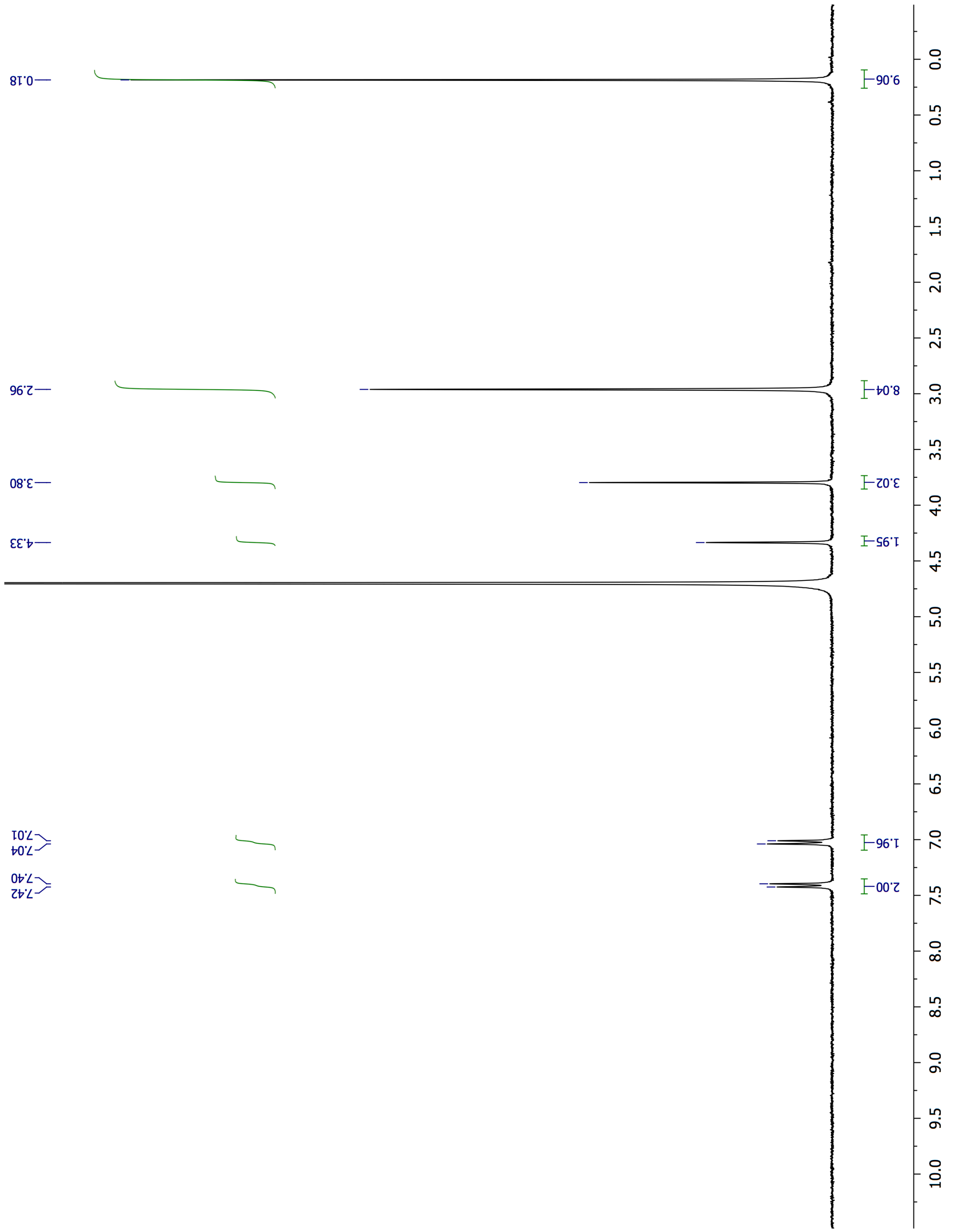

Figure 27. ^{1}H NMR spectrum of TMS derivative **3i** in D_2O.

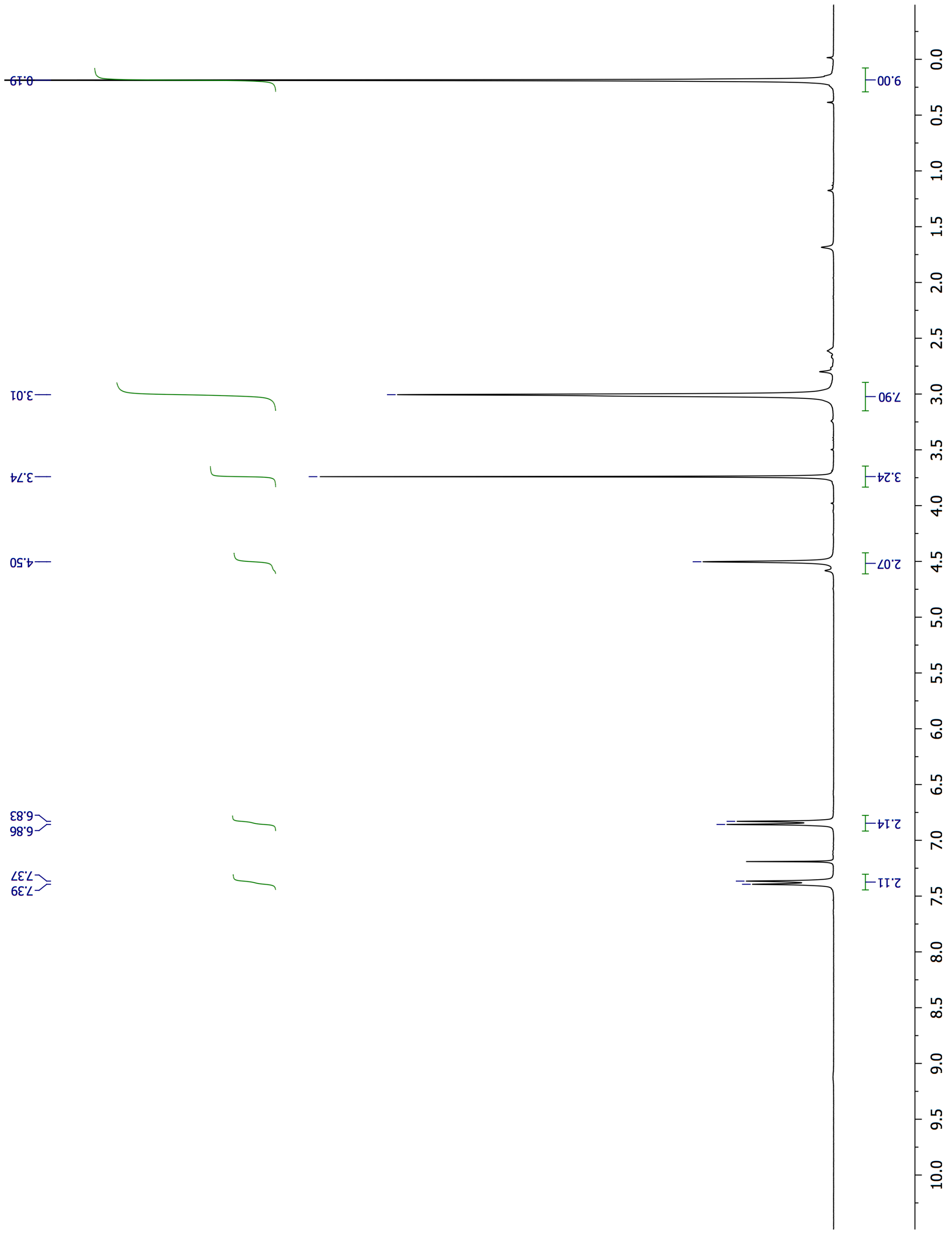

Figure 28. ^{1}H NMR spectrum of TMS derivative **3i** in $CDCl_3$.

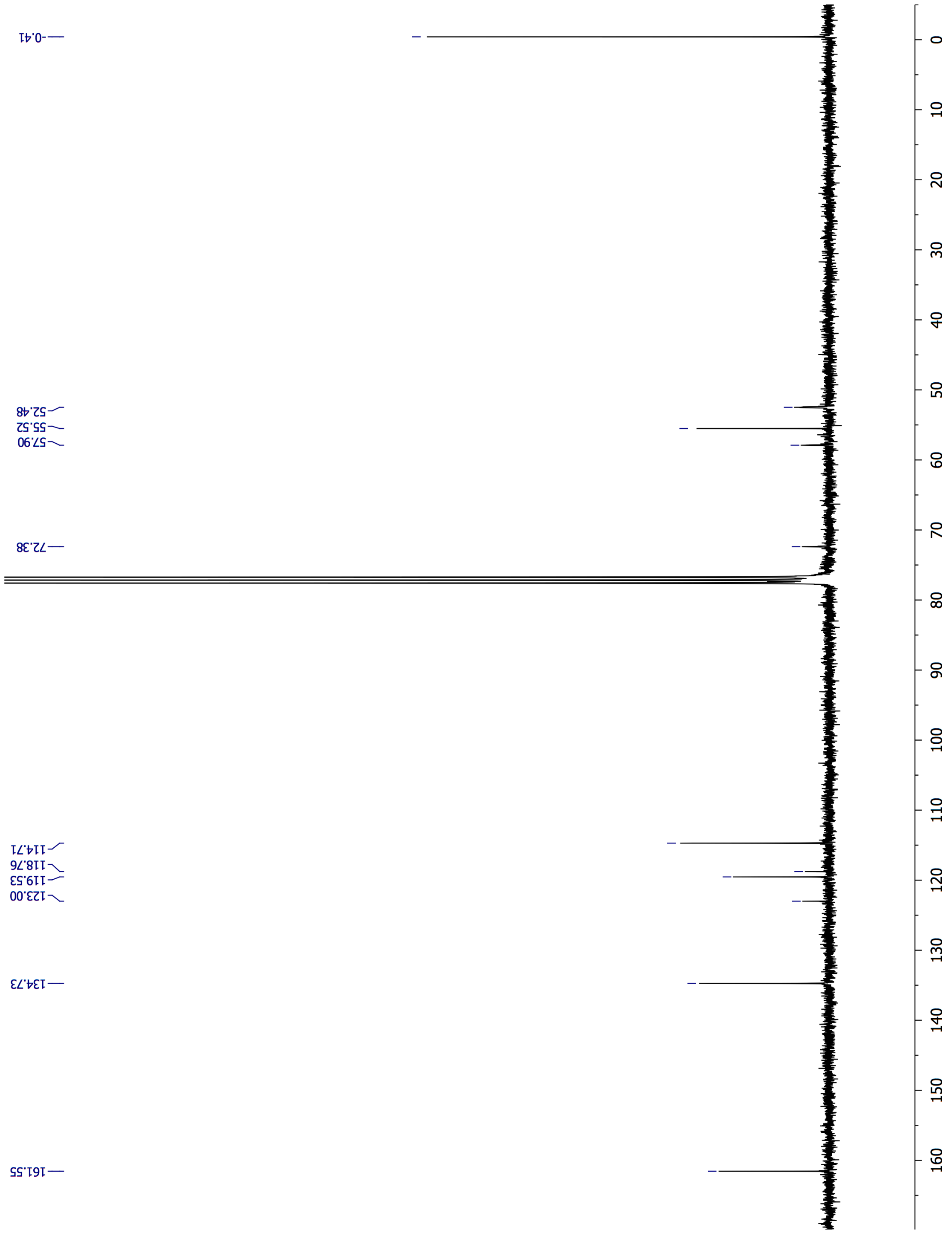

Figure 29. ^{13}C NMR spectrum of TMS derivative **3i** in $CDCl_3$.

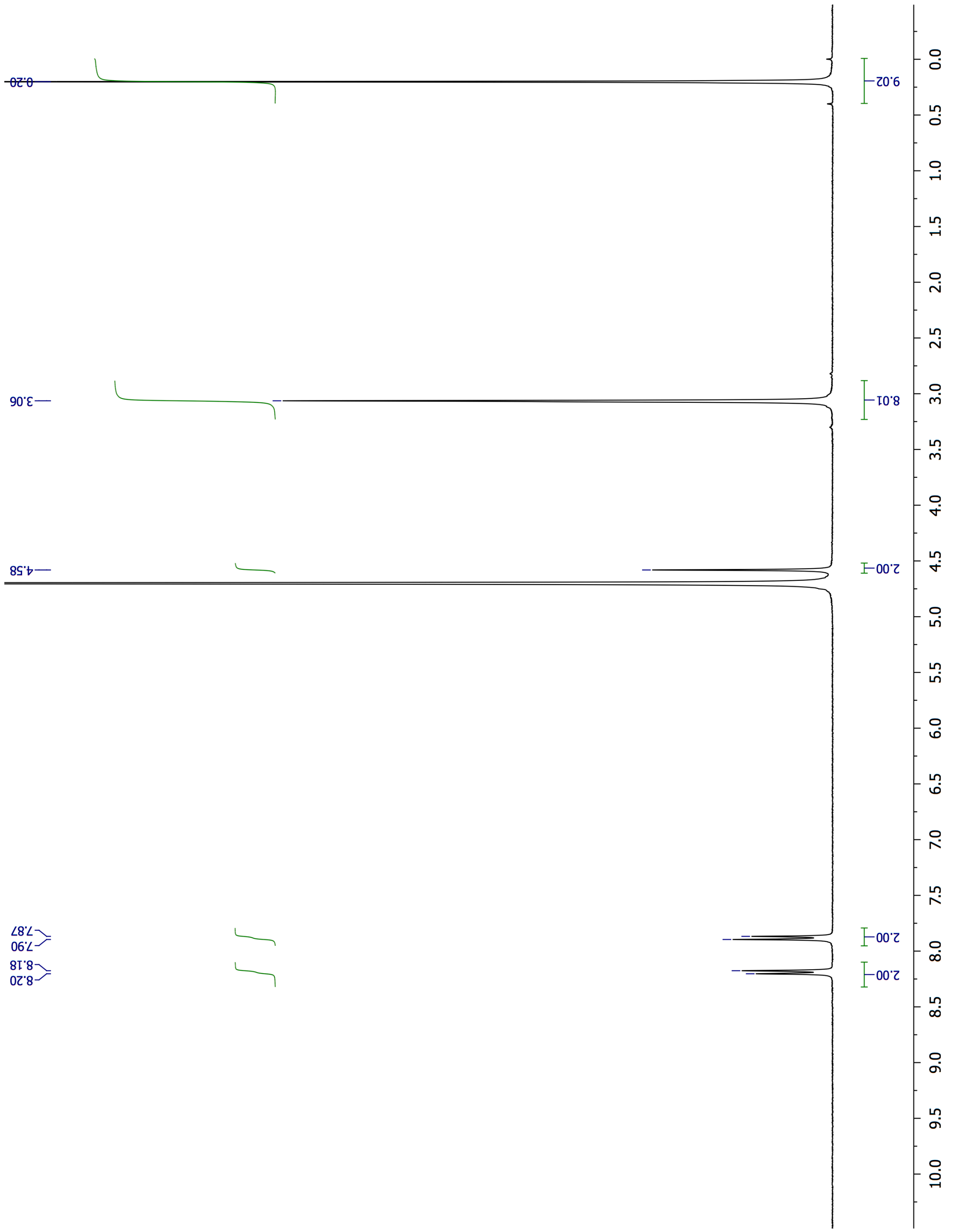

Figure 30. ^{1}H NMR spectrum of TMS derivative **3j** in D_2O.

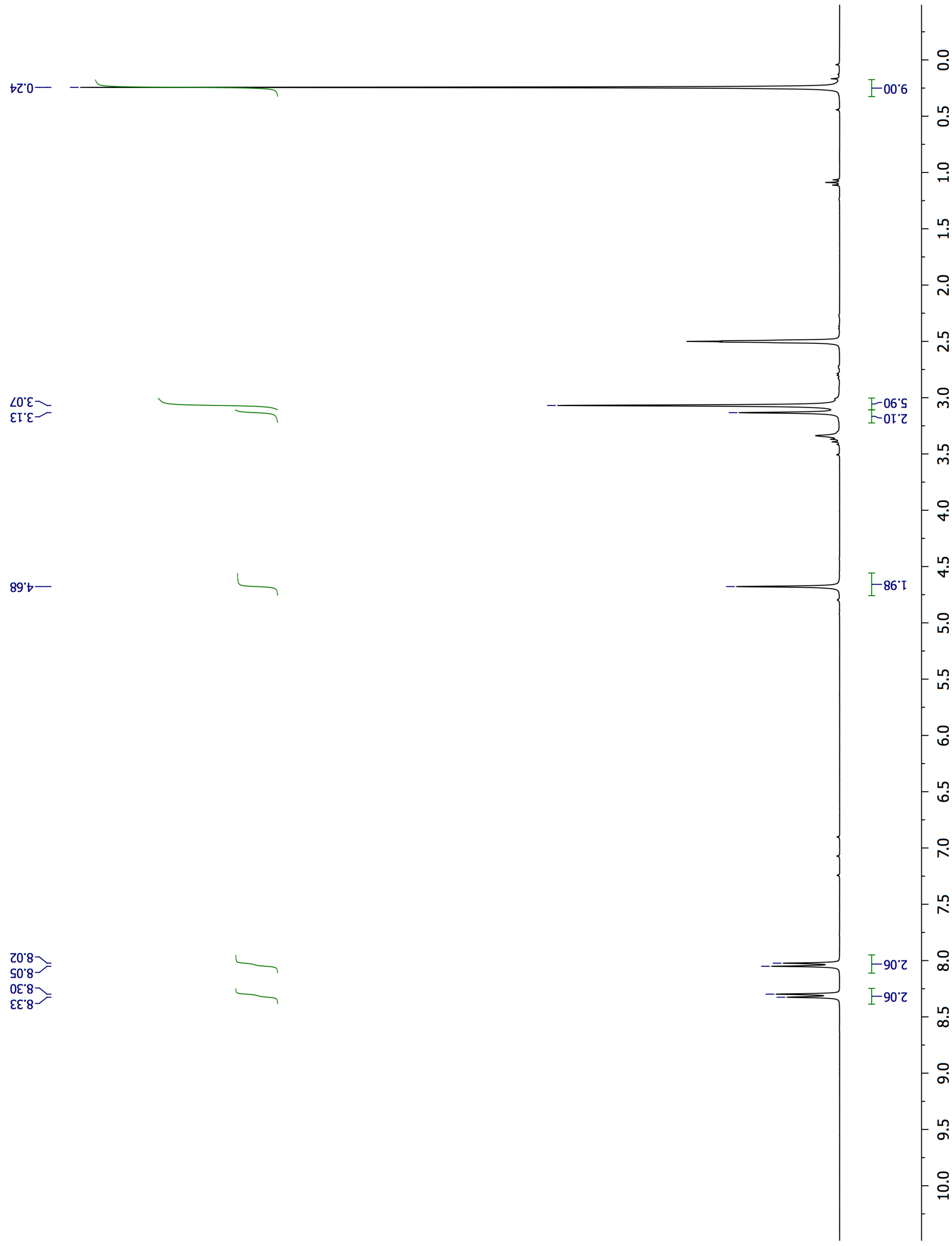

Figure 31. ^{1}H NMR spectrum of TMS derivative **3j** in DMSO-d^{6}.

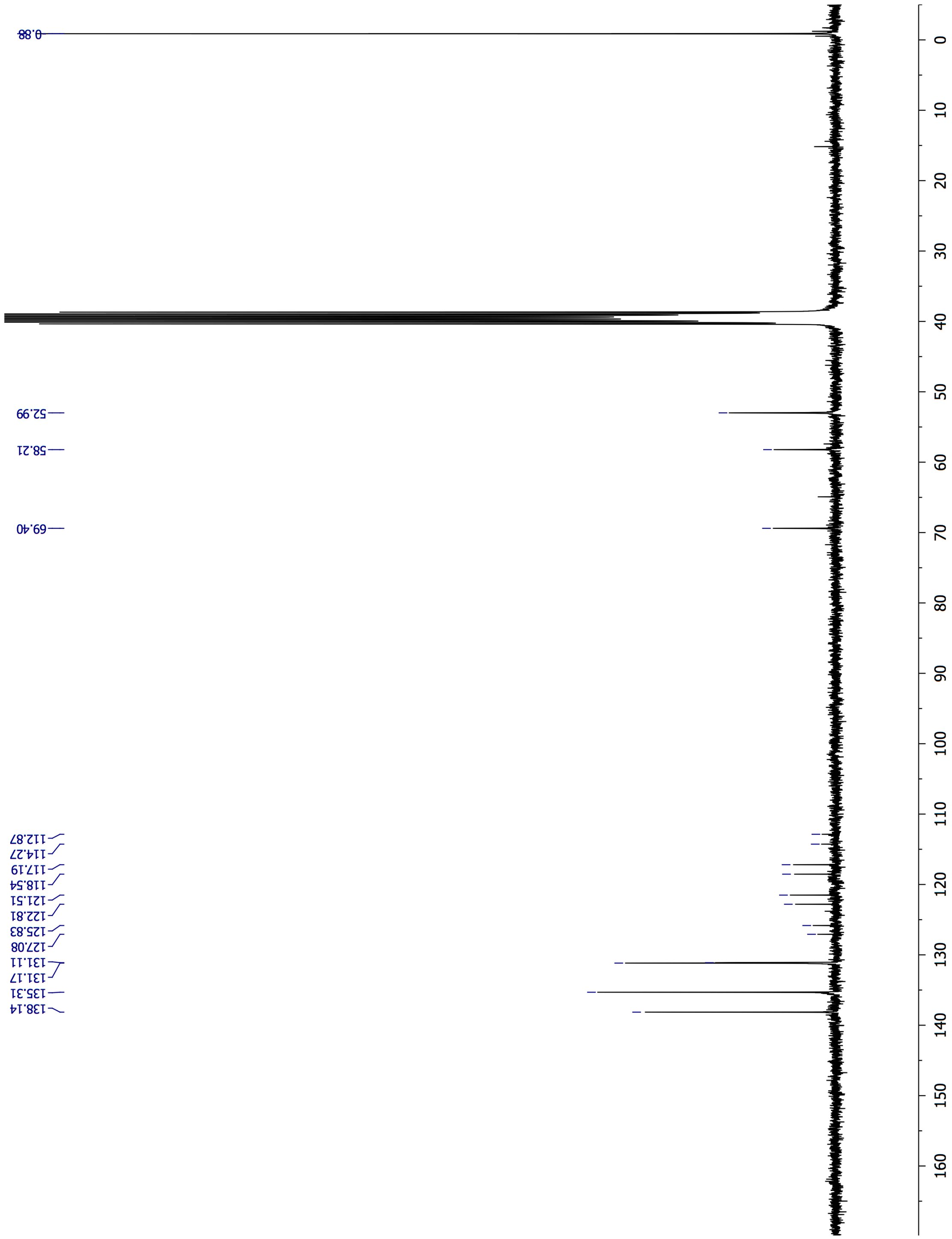

Figure 32. ^{13}C NMR spectrum of TMS derivative **3j** in DMSO-d^6.

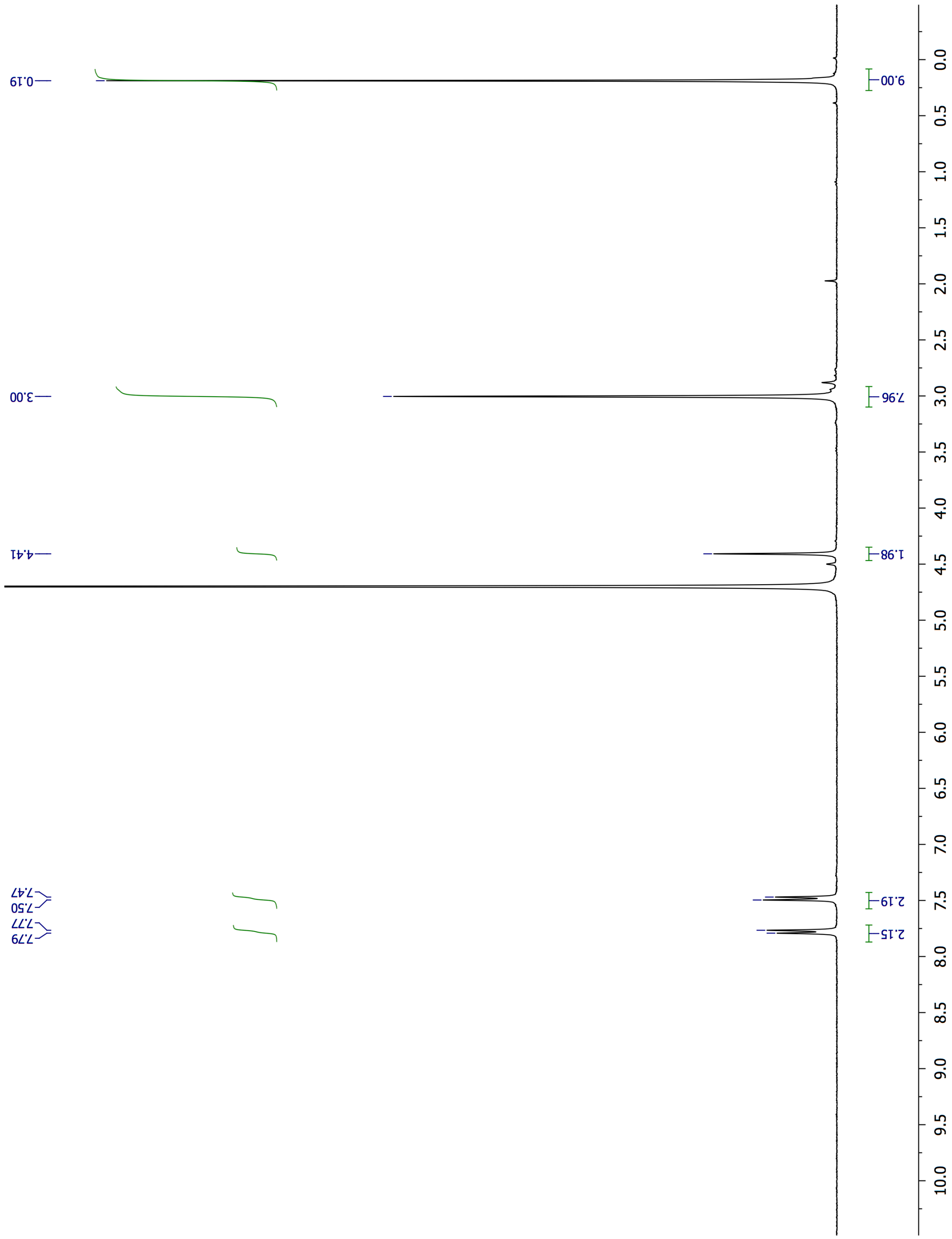

Figure 33. ^{1}H NMR spectrum of TMS derivative **3k** in D_2O.

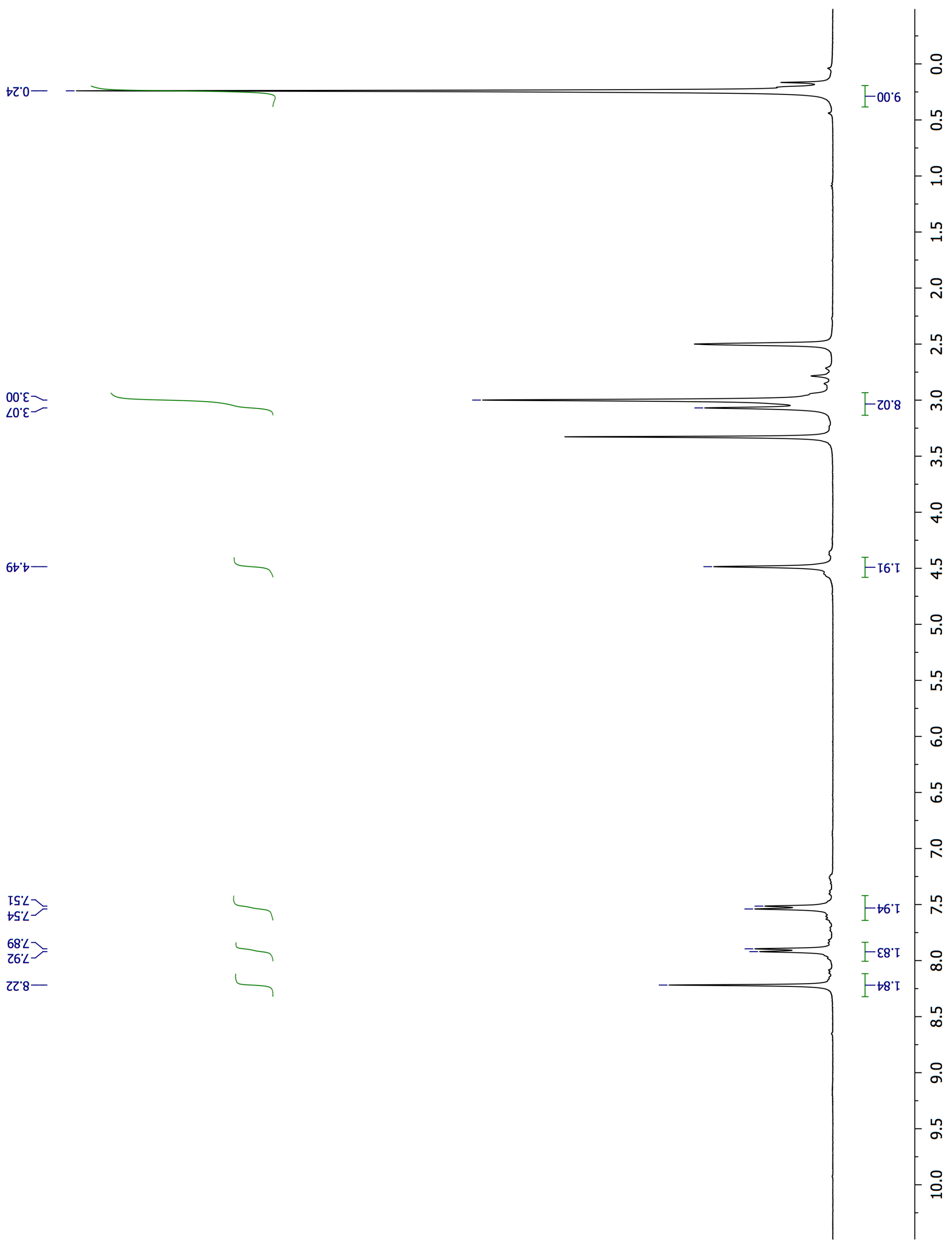

Figure 34. ^{1}H NMR spectrum of TMS derivative **3k** in DMSO-d^{6}.

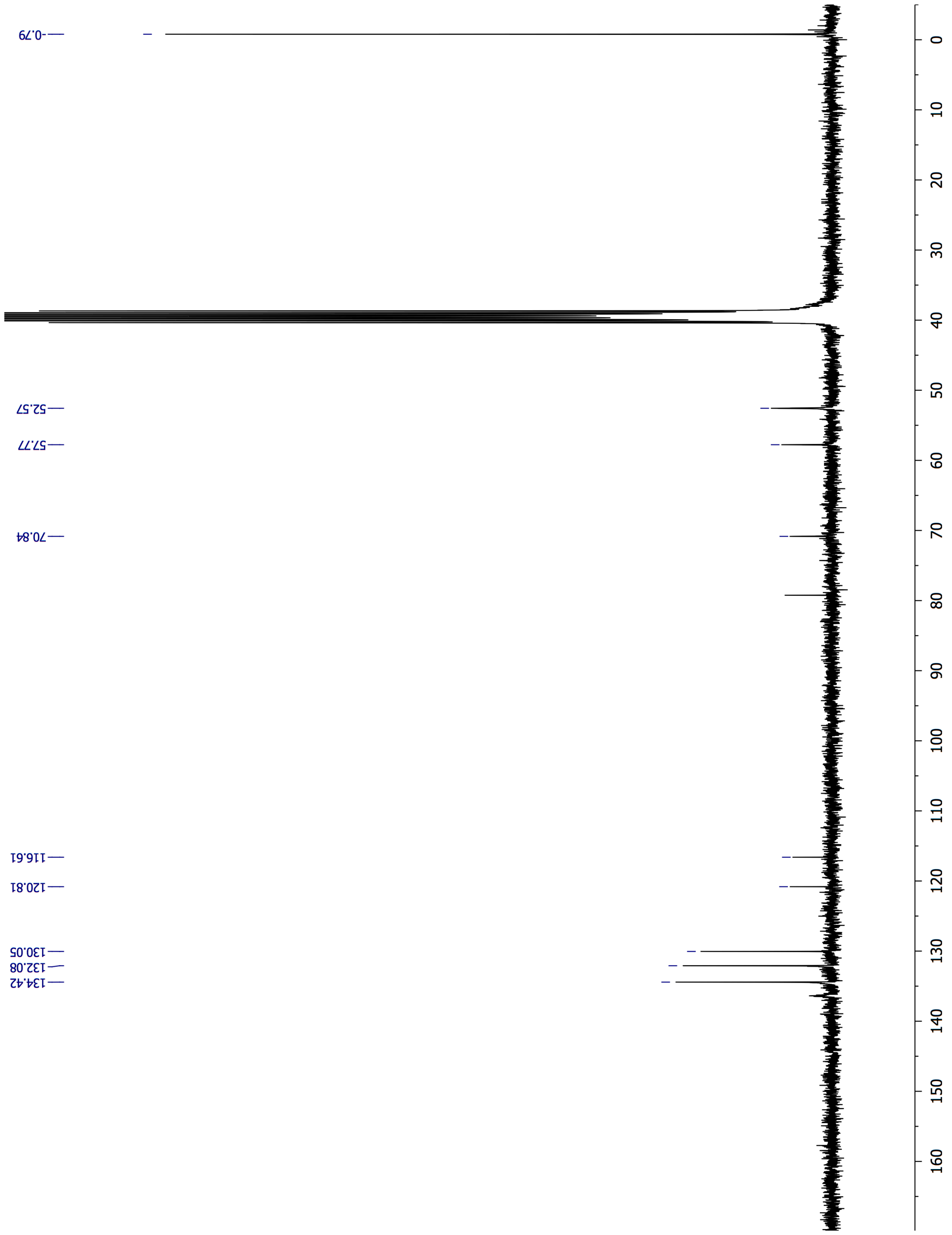

Figure 35. ^{13}C NMR spectrum of TMS derivative **3k** in DMSO-d^6.

7. Computational details

Calculations were carried out with the TURBOMOLE suite of programs (version 6.5),[7] and with the Gaussian 09 package.[8] For TURBOMOLE jobs, input files were first generated with the graphical interface TmoleX (version 4.0.1)[9] with default parameters, and corrected manually or with TURBOMOLE 6.5 to satisfy calculation needs; the m4 grid[10] was used throughout this study. Gaussian 09 jobs were generated using GaussView 5.0,[11] and adjusted manually; the ultrafine grid was employed. In all cases, convergence criteria were 10^{-7} Hartree and 10^{-4} atomic units as the maximum norm of the cartesian gradient.
Guests **3**, complexes **3**·CB[7] and substituted arenes were optimized at the TPSS-D3(BJ)/def2-SVP level. Energies were obtained with single-point calculations using def2-TZVP basis sets, and include the outlying charge correction. The COSMO solvation model was used with the following water specific parameters:

```
$cosmo
 epsilon=78.5
$cosmo_atoms
# radii in Angstrom units
o  1                                         \
  radius=  1.7200
h  2-3                                       \
  radius=  1.3000
```

COSMO solvation energies were obtained from the difference in the total energies calculated with and without the solvation term. IEFPCM solvation energies were obtained with the Gaussian 09 software and default parameters for water solvation, using the difference in the total energies obtained from single-point calculations at the M05-2X/6-31G(d) level with and without the solvation term.

Vibrational analysis was carried out with the NumForce program at the TPSS-D3(BJ)/def2-SVP level. Frequencies were calculated with a unity scaling factor. Entropies were corrected using a method developed by Grimme[12] as low vibrational frequencies are present in large systems, the vibrational entropic contributions S_v are significantly overestimated with the default quantum harmonic oscillator model (equation 7.1).

$$S_V = R\left(\frac{h\nu/kT}{e^{h\nu/kT}-1} - \ln\left(1 - e^{-h\nu/kT}\right)\right) \qquad (7.1)$$

The entropic contributions of the low-lying vibrations were calculated with a free-rotor model instead (S_R; see equations 7.2 and 7.3, where μ is the moment of inertia of the free rotor and B_{av} a limiting factor set to 10^{-44} kg/m^2, which dampens the moment of inertia for extremely low frequencies).

$$\mu = \frac{h}{8\pi^2\nu} \qquad (7.2)$$

$$S_R = \frac{R}{2}\left(1 + \ln\left(\frac{8\pi^3 kT}{h^2}\frac{\mu B_{\mathrm{av}}}{\mu + B_{\mathrm{av}}}\right)\right) \qquad (7.3)$$

The entropy S_{vib} for each vibrational mode was obtained from a linear combination of entropies calculated with both models, using coefficients w and (1-w), where w tends rapidly towards 0 when the frequencies are lower than a threshold ν_0 (100 cm^{-1}), and towards 1 when higher, according to equations 7.4 and 7.5.

$$S_{vib} = wS_V + (1 - w)S_R \qquad (7.4)$$

$$w = \frac{1}{1+\left(\frac{\nu_0}{\nu}\right)^4} \qquad (7.5)$$

8. Computational data

Table 2. Thermodynamic parameters for silanes **3** and para-substituted arenes, calculated using density functional theory and the COSMO solvation model.

	Silanes 3				Complexes 3·CB[7]			
**X**	*E*(COSMO)[a]	*E*(gas)[b]	Δ*E*solv[c]	ΔΔ*E*solv[d]	*E*(COSMO)[a]	*E*(gas)[b]	Δ*E*solv[c]	ΔΔ*E*solv[d]
BOH_2	-1030.482739	-1030.402713	-50.22	-0.05	-5245.479079	-5245.308394	-107.11	-0.17
Br	-3427.818511	-3427.744345	-46.54	-1.75	-7642.814573	-7642.652632	-101.62	-0.06
CF_3	-1191.577487	-1191.500165	-48.52	-3.66	-5406.573287	-5406.411089	-101.78	-0.15
CH_3	-893.6942159	-893.6253302	-43.23	1.07	-5108.690387	-5108.52913	-101.19	-0.12
CN	-946.652918	-946.5675842	-53.55	-5.16	-5161.648809	-5161.480688	-105.50	-0.34
$COOCH_3$	-1082.370171	-1082.29262	-48.66	-0.96	-5297.366182	-5297.199997	-104.28	0.19
F	-953.6426288	-953.5690312	-46.18	-1.59	-5168.638929	-5168.477027	-101.59	-0.23
H	-854.352747	-854.2821376	-44.31	0.00	-5069.348493	-5069.187417	-101.08	0.00
NO_2	-1058.988055	-1058.90231	-53.81	-5.61	-5273.984070	-5273.816427	-105.20	-0.23
OCH_3	-968.948163	-968.8781715	-43.92	2.11	-5183.943994	-5183.780824	-102.39	0.41
SO_2CF_3	-1740.310196	-1740.223002	-54.72	-6.29	-5955.306448	-5955.138153	-105.61	-0.42
CB[7]	-4214.960221	-4214.78659	-108.96					
**X**		**PhX**						
BOH_2	-408.5229444	-408.5091078	-8.68					
Br	-2805.859416	-2805.854157	-3.30					
CF_3	-569.6190484	-569.6136768	-3.37					
CH_3	-271.733268	-271.7287907	-2.81					
CN	-324.695514	-324.684525	-6.90					
$COOCH_3$	-460.4116053	-460.4016979	-6.22					
F	-331.6829681	-331.6780178	-3.11					
H	-232.3924842	-232.3879918	-2.82					
NO_2	-437.0310487	-437.0203617	-6.71					
OCH_3	-346.986745	-346.9795128	-4.54					
SO_2CF_3	-1118.355289	-1118.34424	-6.93					

[a] Total energy contribution calculated at the TPSS-D3(BJ)/def2-TZVP level with the COSMO solvation model [Hartree]. [b] Similar to (a) in the gas phase. [c] Solvation energies [kcal/mol]. [d] Solvation energy of the ammonium unit and its neighboring methyl and methylene groups, relative to silane **3a** (X = H) [kcal/mol].

Table 3. Thermodynamic parameters for silanes **3** and para-substituted arenes, calculated using density functional theory and the IEFPCM solvation model.

	Silanes 3				Complexes 3·CB[7]			
**X**	*E*(IEFPCM)[a]	*E*(gas)[b]	Δ*E*solv[c]	ΔΔ*E*solv[d]	*E*(IEFPCM)[a]	*E*(gas)[b]	Δ*E*solv[c]	ΔΔ*E*solv[d]
B(OH)$_2$	-1029.904156	-1029.831009	-45.90	0.06	-5242.109159	-5241.954713	-96.92	-0.12
Br	-3424.97658	-3424.905167	-44.81	-1.92	-7637.181881	-7637.032273	-93.88	-0.15
CF$_3$	-1190.907048	-1190.833069	-46.42	-3.38	-5403.112384	-5402.962417	-94.11	-0.23
CH$_3$	-893.2020898	-893.1358817	-41.55	1.03	-5105.406385	-5105.25765	-93.33	0.09
CN	-946.125813	-946.045622	-50.32	-4.88	-5158.331659	-5158.177295	-96.86	-0.58
COOCH$_3$	-1081.746003	-1081.673348	-45.59	-0.81	-5293.951315	-5293.799094	-95.52	0.11
F	-953.115108	-953.0445732	-44.26	-1.58	-5165.320222	-5165.171075	-93.59	-0.07
H	-853.8881925	-853.8203504	-42.57	0.00	-5066.092947	-5065.944085	-93.41	0.00
NO$_2$	-1058.360859	-1058.279919	-50.79	-5.55	-5270.566755	-5270.413058	-96.45	-0.36
OCH$_3$	-968.4013389	-968.3340812	-42.20	1.68	-5180.605697	-5180.455121	-94.49	0.24
SO$_2$CF$_3$	-1739.413051	-1739.3292	-52.62	-6.26	-5951.619457	-5951.463688	-97.75	-0.55
CB[7]	-4212.153908	-4211.998889	-97.28					
**X**	**PhX**							
B(OH)$_2$	-408.2464012	-408.2374836	-5.60					
Br	-2803.320015	-2803.315986	-2.53					
CF$_3$	-569.2506302	-569.2463653	-2.68					
CH$_3$	-271.5430642	-271.5395309	-2.22					
CN	-324.4713568	-324.4632661	-5.08					
COOCH$_3$	-460.0899809	-460.0829327	-4.42					
F	-331.457332	-331.4536323	-2.32					
H	-232.2299667	-232.2264472	-2.21					
NO$_2$	-436.7065744	-436.6987947	-4.88					
OCH$_3$	-346.7417915	-346.7361769	-3.52					
SO$_2$CF$_3$	-1117.760385	-1117.750834	-5.99					

[a] Total energy contribution calculated at the M05-2X/6-31G(d) level with the IEFPCM solvation model [Hartree]. [b] Similar to (a) in the gas phase. [c] Solvation energies [kcal/mol]. [d] Solvation energy of the ammonium unit and its neighboring methyl and methylene groups, relative to silane **3a** (X = H) [kcal/mol].

Table 4. Calculated gas phase binding affinities of CB[7] towards silanes **3**.[a]

X	ΔE_{gas} [b]	ΔE_{rel} [c]	$\Delta\Delta G$ [d]	$T\Delta\Delta S_{rotor}$ [e]	ΔG [f]	ΔG_{rel} [g]
$B(OH)_2$	-74.73	-0.25	19.20	0.95	-56.49	-0.60
Br	-76.37	-1.89	19.21	1.06	-58.22	-2.33
CF_3	-78.02	-3.54	19.14	1.12	-60.00	-4.11
CH_3	-73.55	0.93	19.22	0.71	-55.04	0.85
CN	-79.39	-4.91	19.06	0.99	-61.32	-5.43
$COOCH_3$	-75.80	-1.32	19.78	0.91	-56.92	-1.03
F	-76.18	-1.70	19.23	0.88	-57.83	-1.94
H	-74.48	0.00	19.27	0.68	-55.89	0.00
NO_2	-80.02	-5.55	18.68	1.21	-62.56	-6.67
OCH_3	-72.83	1.65	18.49	0.99	-55.33	0.56
SO_2CF_3	-80.67	-6.19	19.14	1.29	-62.82	-6.92

[a] All energies in kcal/mol. [b] Electronic contribution to the binding affinity [kcal/mol], calculated at the TPSS-D3(BJ)/def2-TZVP level. [c] Electronic contribution to the binding affinity relative to silane **3a** (X = H). [d] Combined enthalpic and entropic contributions obtained by vibrational analysis at the TPSS-D3(BJ)/def2-SVP level at 25 ^{o}C. [e] Correction to the entropy term using a free-rotor model for vibrations < 100 cm^{-1}. [f] Free binding energy in the gas phase. [g] Free binding energy relative to silane **3a** (X = H).

9. Optimized structure and coordinates of complex 3a·CB[7]

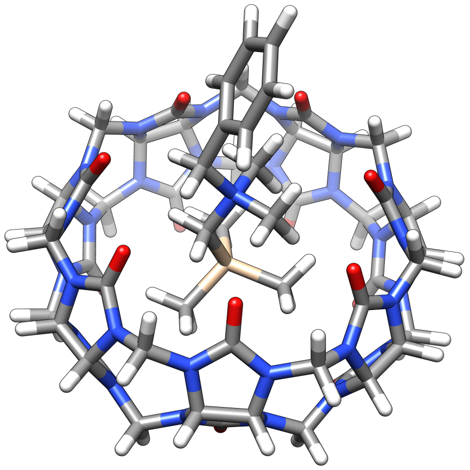

H	0.43223	-2.90054	-3.36323
C	0.52366	-3.19009	-2.30367
H	0.01885	-2.39808	-1.72384
Si	2.38402	-3.15538	-1.77892
C	3.39209	-4.60730	-2.44287
H	4.46212	-4.42791	-2.23289
H	3.28450	-4.70381	-3.53776
H	3.12859	-5.57468	-1.98116
C	2.50809	-3.01315	0.09886
H	3.53840	-2.75097	0.39726
H	2.23996	-3.94190	0.62964
H	1.84282	-2.21048	0.46516
C	2.91814	-1.54516	-2.59448
H	3.97330	-1.30117	-2.38665
H	2.30081	-0.70397	-2.23386
H	2.79617	-1.60151	-3.69021
N	-0.33442	-4.43164	-2.14707
C	-1.80218	-3.97583	-2.31088
H	-1.84124	-3.50611	-3.30487
H	-1.94308	-3.18890	-1.55468
C	-2.80674	-5.08385	-2.15332
C	-4.68732	-7.15993	-1.84176
C	-3.24646	-5.82276	-3.26993
C	-3.33290	-5.38532	-0.87958
C	-4.26551	-6.42012	-0.72534
C	-4.17973	-6.85740	-3.11520
H	-2.85997	-5.58147	-4.26638
H	-3.00543	-4.80684	-0.00856
H	-4.66704	-6.64733	0.26778
H	-4.51466	-7.42458	-3.98991
C	0.03190	-5.43184	-3.21116
H	1.08766	-5.68940	-3.08096
H	-0.13754	-4.98815	-4.20229
H	-0.57317	-6.33490	-3.06954
C	-0.12739	-5.06160	-0.79908
H	0.92943	-5.34633	-0.71811
H	-0.73902	-5.96718	-0.72678
H	-0.41428	-4.34871	-0.01437
N	0.23829	-0.38860	-5.62420
C	0.99796	0.85231	-5.62744
C	0.49625	1.58434	-4.33786
N	-0.50385	0.67539	-3.80325
C	-0.67272	-0.45677	-4.58260
H	0.80698	1.41253	-6.56008
H	0.04511	2.57330	-4.53434
N	2.42482	0.71435	-5.40265
N	1.70339	1.72637	-3.54201
C	2.83826	1.27757	-4.20399
O	-1.50512	-1.34251	-4.40526
O	3.99569	1.39477	-3.81858
N	2.15806	-4.25597	-7.09229
C	3.19553	-3.34289	-7.53876
C	2.51984	-1.93730	-7.42908
N	1.22037	-2.24180	-6.85298
C	1.00558	-3.60066	-6.69007
H	3.52066	-3.60468	-8.56136
H	2.39976	-1.42128	-8.39826
N	4.34174	-3.22955	-6.65295
N	3.41707	-1.19155	-6.56644
C	4.50091	-1.94874	-6.13945
O	-0.03301	-4.12718	-6.29546
O	5.44494	-1.54850	-5.46884
C	0.14356	-1.28075	-6.76064
H	0.11380	-0.68022	-7.68771
H	-0.78889	-1.85359	-6.64535
C	3.36886	0.24889	-6.39809
H	3.12842	0.71178	-7.37170
H	4.36585	0.56745	-6.05818
N	3.32637	-7.83995	-4.60077
C	4.65639	-7.60440	-5.14889
C	4.39821	-6.53661	-6.26313
N	2.95317	-6.41426	-6.28188
C	2.33261	-7.20903	-5.33207
H	5.08518	-8.54940	-5.52765
H	4.77061	-6.83415	-7.25967
N	5.59305	-6.94531	-4.26345
N	5.12099	-5.36835	-5.78082
C	5.87787	-5.64486	-4.64847
O	1.12182	-7.35545	-5.18948
O	6.68325	-4.89028	-4.11473
N	2.90247	-8.40169	-0.08239
C	4.33881	-8.60696	-0.14654
C	4.61707	-8.77335	-1.67648
N	3.30662	-8.57674	-2.27774
C	2.30304	-8.40178	-1.33406
H	4.62754	-9.48478	0.45870
H	5.01941	-9.76411	-1.95323
N	5.14268	-7.45229	0.21603
N	5.59010	-7.73548	-1.95554
C	5.90366	-6.97368	-0.84063
O	1.10115	-8.29933	-1.56270
O	6.73461	-6.07352	-0.78700
C	2.20428	-5.69180	-7.28705
H	2.63786	-5.90032	-8.28090
H	1.16844	-6.05924	-7.23628
C	5.40798	-4.20749	-6.60100
H	6.27853	-3.70641	-6.15065
H	5.64474	-4.53995	-7.62873
N	1.23006	-5.62624	3.13355
C	2.57394	-5.73659	3.67435
C	3.19675	-6.91031	2.84750
N	2.12094	-7.30016	1.94957
C	0.95440	-6.58489	2.17149
H	2.53408	-5.92881	4.76147
H	3.51137	-7.77143	3.46397
N	3.45687	-4.61931	3.39219
N	4.34720	-6.29869	2.20885
C	4.51712	-4.96863	2.56735
O	-0.13341	-6.78583	1.63926
O	5.45696	-4.24799	2.25081
N	-0.45669	-1.44197	2.47271
C	0.58487	-1.03452	3.39893
C	1.09105	-2.39240	3.99093
N	0.28834	-3.38513	3.29107
C	-0.63338	-2.81478	2.42851
H	0.17600	-0.34205	4.15605
H	0.94548	-2.48633	5.08167
N	1.77420	-0.46984	2.78246
N	2.50454	-2.38976	3.66655
C	2.90386	-1.25062	2.98647
O	-1.48096	-3.41418	1.76985
O	4.05099	-0.96219	2.66247
C	0.19753	-4.77778	3.68672
H	0.22811	-4.83674	4.78926
H	-0.76224	-5.16065	3.30786
C	3.45282	-3.37555	4.13755
H	3.24615	-3.59684	5.19966
H	4.45307	-2.93086	4.02561
C	5.41066	-7.05529	1.58157
H	6.29633	-6.40258	1.55369
H	5.61460	-7.95649	2.18859
C	-1.40448	-0.53050	1.86872
H	-1.70872	0.21920	2.62063
H	-2.27107	-1.13016	1.55215
C	1.87347	0.89395	2.30338
H	2.94653	1.13182	2.24636
H	1.36758	1.56723	3.01902
C	3.01155	-8.89597	-3.66038
H	3.56411	-9.81162	-3.93953
H	1.92682	-9.06819	-3.73127
C	6.31803	-7.60901	-3.20010
H	7.20687	-6.99666	-2.98670
H	6.61634	-8.61394	-3.54651
C	2.13369	-8.50290	1.14220
H	2.53390	-9.33989	1.74247
H	1.08987	-8.70222	0.85659
N	1.32190	1.11555	0.98082
C	-0.08880	1.35013	0.73043
C	-0.11577	1.85683	-0.74925
N	1.28379	1.84357	-1.13914
C	2.12375	1.45425	-0.10185
H	-0.49548	2.07417	1.45858
H	-0.53878	2.87016	-0.86724
N	-0.91457	0.15512	0.69182
N	-0.95288	0.87830	-1.42071
C	-1.42559	-0.10423	-0.56838
O	3.34880	1.45041	-0.12556
O	-2.19055	-1.01863	-0.86746
C	-1.45274	1.03103	-2.76931
H	-2.31161	0.35086	-2.87105
H	-1.77102	2.07794	-2.91961
C	1.79079	2.50842	-2.32601
H	2.86028	2.70374	-2.15471
H	1.24327	3.45858	-2.46196
H	-5.41741	-7.96729	-1.72066

10. Optimized structure and coordinates of complex 3e·CB[7]

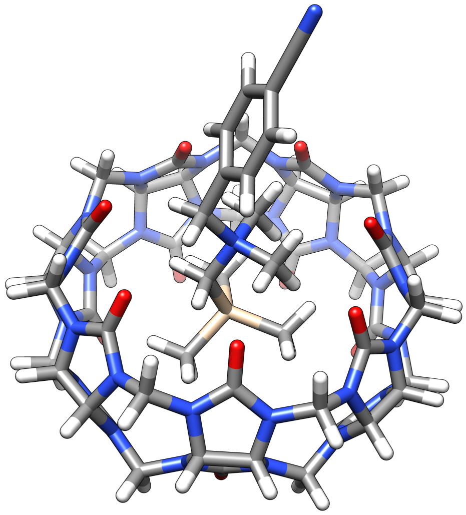

H	0.47436	-2.89641	-3.38952
C	0.56982	-3.17321	-2.32708
H	0.06965	-2.37363	-1.75396
Si	2.43169	-3.14393	-1.80215
C	3.42424	-4.61207	-2.45170
H	4.49576	-4.44117	-2.24302
H	3.31609	-4.71618	-3.54578
H	3.15178	-5.57325	-1.98232
C	2.54781	-2.98270	0.07437
H	3.57675	-2.71946	0.37589
H	2.27461	-3.90400	0.61547
H	1.88269	-2.17397	0.42771
C	2.97813	-1.54623	-2.63120
H	4.04478	-1.32967	-2.45434
H	2.39263	-0.69186	-2.24985
H	2.82313	-1.59672	-3.72287
N	-0.28898	-4.41365	-2.15115
C	-1.75575	-3.95778	-2.28862
H	-1.82828	-3.52548	-3.29741
H	-1.88090	-3.14434	-1.55802
C	-2.75402	-5.05979	-2.05549
C	-4.60542	-7.12521	-1.59454
C	-3.22714	-5.84343	-3.12651
C	-3.23278	-5.30755	-0.75149
C	-4.14960	-6.33396	-0.51524
C	-4.14445	-6.87388	-2.90654
H	-2.88048	-5.64341	-4.14535
H	-2.87683	-4.69593	0.08425
H	-4.51674	-6.52708	0.49605
H	-4.50730	-7.48048	-3.74022
C	0.05866	-5.41731	-3.22022
H	1.11778	-5.67012	-3.10954
H	-0.12962	-4.97724	-4.20970
H	-0.53733	-6.32389	-3.06253
C	-0.05623	-5.04134	-0.80561
H	1.00519	-5.31215	-0.73943
H	-0.65321	-5.95543	-0.72190
H	-0.34120	-4.33077	-0.01840
N	0.24674	-0.39268	-5.62995
C	1.00092	0.85172	-5.63229
C	0.49432	1.58076	-4.34310
N	-0.50747	0.67066	-3.81414
C	-0.66690	-0.46418	-4.59105
H	0.80821	1.41123	-6.56496
H	0.04387	2.57013	-4.53895
N	2.42818	0.71986	-5.40615
N	1.69864	1.72068	-3.54211
C	2.83721	1.28292	-4.20579
O	-1.49397	-1.35524	-4.41362
O	3.99354	1.40706	-3.81952
N	2.17525	-4.24955	-7.11497
C	3.21362	-3.33210	-7.54943
C	2.53411	-1.92866	-7.43685
N	1.23523	-2.23880	-6.86197
C	1.02314	-3.59867	-6.70586
H	3.54667	-3.58776	-8.57098
H	2.41306	-1.41056	-8.40473
N	4.35321	-3.22086	-6.65424
N	3.42810	-1.18242	-6.57131
C	4.51056	-1.93968	-6.14040
O	-0.01356	-4.12889	-6.31069
O	5.45177	-1.53966	-5.46574
C	0.15508	-1.28208	-6.76859
H	0.12380	-0.67927	-7.69413
H	-0.77547	-1.85840	-6.65539
C	3.37596	0.25785	-6.40018
H	3.13705	0.72173	-7.37363
H	4.37121	0.57837	-6.05708
N	3.32638	-7.83044	-4.61914
C	4.65996	-7.60080	-5.16211
C	4.41078	-6.53077	-6.27605
N	2.96615	-6.40977	-6.30695
C	2.33835	-7.19770	-5.35659
H	5.08564	-8.54744	-5.54012
H	4.79230	-6.82546	-7.26993
N	5.59584	-6.94679	-4.27232
N	5.12815	-5.36257	-5.78397
C	5.88262	-5.64492	-4.65110
O	1.12624	-7.33744	-5.21737
O	6.68674	-4.89297	-4.11182
N	2.87543	-8.41360	-0.10576
C	4.31252	-8.61518	-0.16428
C	4.59796	-8.77733	-1.69322
N	3.29051	-8.57579	-2.29973
C	2.28247	-8.40463	-1.36032
H	4.60095	-9.49340	0.44043
H	4.99941	-9.76803	-1.97131
N	5.11141	-7.45865	0.20381
N	5.57403	-7.74037	-1.96495
C	5.87955	-6.97912	-0.84736
O	1.08181	-8.29763	-1.59438
O	6.70913	-6.07811	-0.78755
C	2.22461	-5.68431	-7.31512
H	2.66666	-5.88822	-8.30602
H	1.18890	-6.05317	-7.27440
C	5.41987	-4.19853	-6.59860
H	6.28707	-3.69850	-6.14067
H	5.66384	-4.52730	-7.62584
N	1.18114	-5.64414	3.09744
C	2.51903	-5.75833	3.65323
C	3.15000	-6.92826	2.82736
N	2.07910	-7.32251	1.92585
C	0.91189	-6.60533	2.13603
H	2.46663	-5.95618	4.73874
H	3.46673	-7.78843	3.44403
N	3.40534	-4.64022	3.38684
N	4.29994	-6.31022	2.19289
C	4.46973	-4.98349	2.56470
O	-0.17205	-6.80549	1.59517
O	5.41194	-4.26121	2.25952
N	-0.50256	-1.45240	2.45597
C	0.53739	-1.05331	3.38740
C	1.03642	-2.41577	3.97454
N	0.24172	-3.40302	3.25657
C	-0.67484	-2.82442	2.39446
H	0.12848	-0.36283	4.14622
H	0.87731	-2.51872	5.06252
N	1.73122	-0.49062	2.77714
N	2.45331	-2.41118	3.66711
C	2.85936	-1.27099	2.99264
O	-1.51575	-3.41864	1.72177
O	4.00955	-0.98176	2.68101
C	0.14357	-4.79787	3.64388
H	0.16142	-4.86230	4.74643
H	-0.81247	-5.17741	3.25228
C	3.39601	-3.40134	4.14046
H	3.17912	-3.62953	5.19896
H	4.39774	-2.95709	4.04090
C	5.37006	-7.06272	1.57141
H	6.25366	-6.40694	1.54982
H	5.57344	-7.96405	2.17848
C	-1.44491	-0.53483	1.85259
H	-1.74654	0.21374	2.60639
H	-2.31410	-1.12877	1.53260
C	1.83624	0.87414	2.30151
H	2.91027	1.10897	2.25023
H	1.32883	1.54769	3.01583
C	3.00232	-8.88913	-3.68491
H	3.55349	-9.80553	-3.96438
H	1.91748	-9.05763	-3.76273
C	6.31126	-7.61364	-3.20433
H	7.19998	-7.00370	-2.98374
H	6.60968	-8.61878	-3.54982
C	2.10069	-8.52226	1.11453
H	2.50151	-9.35951	1.71379
H	1.05917	-8.72519	0.82324
N	1.29131	1.09942	0.97661
C	-0.11724	1.34268	0.72135
C	-0.13581	1.84966	-0.75841
N	1.26559	1.83516	-1.14125
C	2.09932	1.43838	-0.10168
H	-0.52250	2.06868	1.44822
H	-0.55785	2.86304	-0.87881
N	-0.94929	0.15219	0.67918
N	-0.96958	0.87101	-1.43446
C	-1.44845	-0.11013	-0.58460
O	3.32435	1.42831	-0.12057
O	-2.20954	-1.02669	-0.88865
C	-1.46241	1.02446	-2.78549
H	-2.32036	0.34392	-2.89229
H	-1.78049	2.07123	-2.93685
C	1.78015	2.50128	-2.32435
H	2.84894	2.69528	-2.14726
H	1.23436	3.45225	-2.46148
C	-5.54579	-8.18217	-1.35824
N	-6.31279	-9.04598	-1.16442

11. References

1. W.-H. Huang, S. Liu and L. Isaacs, in *Modern Supramolecular Chemistry*, Wiley-VCH Verlag GmbH & Co. KGaA, 2008, DOI: 10.1002/9783527621484.ch4, pp. 113-142.
2. J. E. Noll, J. L. Speier and B. F. Daubert, *J. Am. Chem. Soc.*, 1951, **73**, 3867-3871.
3. *G.B. Pat.,* 686,068, 1953.
4. *U.S. Pat.,* 6,489,338, 2002.
5. R. Goumont, N. Faucher, G. Moutiers, M. Tordeux and C. Wakselman, *Synthesis*, 1997, **1997**, 691-695.
6. C. Hansch, A. Leo and R. W. Taft, *Chem. Rev.*, 1991, **91**, 165-195.
7. A development of the University of Karlsruhe and Forschungszentrum Karlsruhe GmbH, 1989-2007; TURBOMOLE GmbH since 2007; available from www.turbomole.com and distributed by COSMOlogic GmbH & Co. KG, D-51381 Leverkusen (http://www.cosmologic.de); see also (a) R. Ahlrichs, M. Bär, M. Häser, H. Horn and C. Kölmel, *Chem. Phys. Lett.* 1989, **162**, 165-169. (b) M. Sierka, A. Hogekamp and R. Ahlrichs, *J. Chem. Phys.* 2003, **118**, 9136-9148. (c) P. Deglmann, K. May, F. Furche and R. Ahlrichs, *Chem. Phys. Lett.* 2004, **384**, 103-107.
8. Gaussian 09, Revision D.01, M. J. Frisch, G. W. Trucks, H. B. Schlegel, G. E. Scuseria, M. A. Robb, J. R. Cheeseman, G. Scalmani, V. Barone, B. Mennucci, G. A. Petersson, H. Nakatsuji, M. Caricato, X. Li, H. P. Hratchian, A. F. Izmaylov, J. Bloino, G. Zheng, J. L. Sonnenberg, M. Hada, M. Ehara, K. Toyota, R. Fukuda, J. Hasegawa, M. Ishida, T. Nakajima, Y. Honda, O. Kitao, H. Nakai, T. Vreven, J. A. Montgomery Jr., J. E. Peralta, F. Ogliaro, M. Bearpark, J. J. Heyd, E. Brothers, K. N. Kudin, V. N. Staroverov, R. Kobayashi, J. Normand, K. Raghavachari, A. Rendell, J. C. Burant, S. S. Iyengar, J. Tomasi, M. Cossi, N. Rega, J. M. Millam, M. Klene, J. E. Knox, J. B. Cross, V. Bakken, C. Adamo, J. Jaramillo, R. Gomperts, R. E. Stratmann, O. Yazyev, A. J. Austin, R. Cammi, C. Pomelli, J. W. Ochterski, R. L. Martin, K. Morokuma, V. G. Zakrzewski, G. A. Voth, P. Salvador, J. J. Dannenberg, S. Dapprich, A. D. Daniels, Ö. Farkas, J. B. Foresman, J. V. Ortiz, J. Cioslowski and D. J. Fox, Gaussian, Inc., Wallingford CT, 2009.
9. COSMOlogic GmbH & Co. KG, D-51381 Leverkusen (http://www.cosmologic.de).
10. K. Eichkorn, F. Weigend, O. Treutler and R. Ahlrichs, *Theor. Chem. Acta*, 1997, **97**, 119-124.
11. GaussView, Version 5, R. Dennington, T. Keith and J. Millam, Semichem Inc., Shawnee Mission, KS, 2009.
12. S. Grimme, *Chem. Eur. J.*, 2012, **18**, 9955-9964.
